# Evidence of separate subgroups of juvenile southern bluefin tuna

**DOI:** 10.1002/ece3.3500

**Published:** 2017-11-02

**Authors:** Mark S. Chambers, Leesa A. Sidhu, Ben O'Neill, Nokuthaba Sibanda

**Affiliations:** ^1^ School of Physical, Environmental and Mathematical Sciences University of New South Wales (Canberra) at the Australian Defence Force Academy Canberra BC Australia; ^2^ School of Mathematics and Statistics Victoria University of Wellington Wellington New Zealand

**Keywords:** archival tags, contingent theory, migration, mixing, southern bluefin tuna, tagging data

## Abstract

Archival tagging studies of southern bluefin tuna (SBT *, Thunnus maccoyii*) have revealed that juveniles residing in the Great Australian Bight (GAB) over the austral summer undertake seasonal cyclic migrations to the southeast Indian Ocean and the Tasman Sea during winter. However, there remains disagreement about the extent of mixing between juvenile SBT regularly caught by longline fleets south of Africa and those observed in the GAB. Some researchers have argued that archival tag recoveries indicate most juveniles reside in the GAB over the austral summer. Others have suggested that recoveries of conventional and archival tags are better explained by a juvenile population consisting of separate groups on the eastern and western sides of the Indian Ocean with limited intermixing. We present analyses of catch and tag recovery data and re‐examine archival tagging studies. The evidence provided strongly favors the hypothesis of separate juvenile subgroups, or contingents, with limited intermixing. We draw some tentative conclusions about the nature of the putative contingents and discuss some implications of these findings for the interpretation of existing datasets and future research priorities. We also provide the first evidence that the migration choices of juveniles that summer in the GAB are influenced by fidelity to winter feeding grounds and suggest this helps explain the collapse of the surface fishery off New South Wales in the 1980s.

## 1 INTRODUCTION

**Figure 1:**
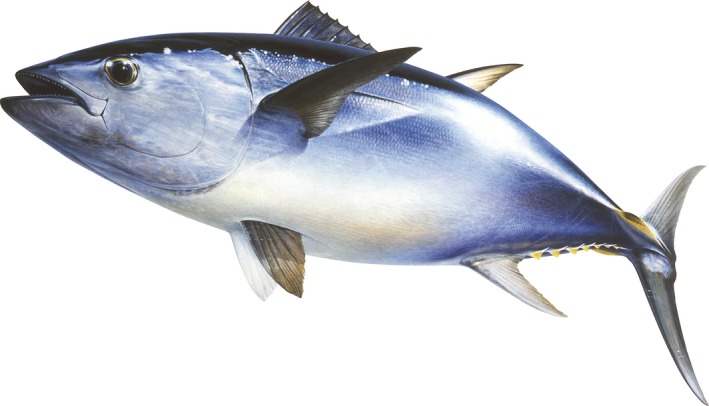
Southern bluefin tuna (*Thunnus maccoyii*) (Illustration © R. Swainston/anima.net)

Southern bluefin tuna (SBT, *Thunnus maccoyii,* Figure 1) is a large, highly migratory tuna species with wide distribution throughout the temperate oceans of the Southern Hemisphere (Hobday et al., 2015). Genetic analysis (Grewe, Elliott, Innes, & Ward, 1997) has found no evidence of heterogeneity between groups sampled from disparate feeding grounds across the distribution of the species. Adults spawn between August and March on a single spawning ground in the eastern Indian Ocean. The species has been the subject of considerable research, but aspects of its population dynamics remain contested or poorly understood.

One subject of historical debate concerns the origin of juvenile SBT regularly observed in the catch of longline fleets operating south of Africa. The first models of SBT migration suggested juveniles were present on known nursery grounds in nearshore waters off the southern coast of Australia for the first few years of life. In this case, the African juveniles would be presumed to have migrated to the southwestern Indian Ocean after surviving fisheries that have historically operated off southern and eastern Australia. An alternative explanation, provided by Murphy (1977), argued that the presence of young juveniles in the Japanese longline catch off southern Africa, as well as characteristics of tag recovery data, was more consistent with a considerable proportion of juveniles migrating to the waters south of Africa without entering the Australian fishing grounds.

Since this time, recoveries of electronic archival tags have provided a more detailed understanding of the movements of juvenile SBT off southern Australia. However, there remains disagreement about the extent of mixing between juveniles caught by longline fleets south of Africa and those observed in the Great Australian Bight (GAB) over the austral summer (hereafter summer).

Based on information from archival tagging studies, Gunn and Block (2001, p. 182) asserted that juvenile SBT make cyclical migrations over ocean‐basin scales with summers spent in the continental shelf waters of the GAB and in winters disperse to feeding grounds in the southern Indian, the southeast Atlantic and Southern oceans as well as the southwest Pacific Ocean. Basson, Hobday, Eveson, and Patterson (2012) report on a more recent archival tagging study and, while acknowledging a degree of uncertainty, stated it was “unlikely that a large proportion of juvenile SBT remain off South Africa over summer” (Basson et al., 2012, p. 2, p. 178, p. 225, p. 256). According to Eveson, Basson, and Hobday (2012, p. 872), archival tag recovery data suggest “close to 100%” of 2‐ to 4‐year‐old SBT spend their summers in the coastal waters south of Australia. Japanese scientists, on the other hand, concluded from conventional and archival tag recoveries there was possibly a tendency for SBT to stay either on the eastern or western sides of the southern Indian Ocean and that, overall, the tag recovery data “strongly indicate” a need to reconsider the assumed complete mixing hypothesis (Takahashi, Tsuji, & Kurota, 2004, p. 9‐10).

The consequences of the true extent of mixing in juvenile SBT and the proportion of juveniles resident in the GAB over summer have been discussed by Gunn, Farley, and Hearn (2003, p. 2) and Basson et al. (2012, p. 177). Much of the research carried out on juveniles has been based on observations or tagging of juveniles in the GAB and off the southern Australian coast more generally. Typically inferences from these observations and tag recoveries require an assumption that the behavior of the observed population is generalizable to all individuals from the same cohorts. Some of the research that might potentially be affected by incomplete mixing is described below.

Large‐scale tagging studies incorporating a Brownie design (Brownie, Anderson, Burnham, & Robson, 1985) were run off southern Australia (WA and SA) during the 1990s and 2000s to enable estimates of juvenile mortality rates (see Polacheck, Laslett, & Eveson, 2006). A key assumption of these studies is that all individuals of an identifiable class have the same survival and recovery probabilities (Pollock & Ravelling, 1982). Simulation studies (Kurota, Hiramatsu, & Tsuji, 2002) have shown that estimates of mortality of SBT based on the tag recovery data are likely to be biased if tags are released from only part of the juvenile distribution of SBT and individuals do not mix thoroughly between regions. Also, an aerial survey has been run in the GAB most summers since 1993. The survey is assumed to provide an index of the relative abundance of 2‐ to 4‐year‐old SBT (Hillary et al., 2016). If the proportion of juveniles that are resident in the GAB over summer varies substantially among years, the relationship between the index and juvenile abundance will be unclear. More recently, gene‐tagging studies of SBT have been suggested (Preece et al., 2013, 2015) to estimate the absolute population size of individual cohorts. These studies propose establishing a tagged population by taking biopsies from 2‐year‐olds caught and released in the GAB. According to the proposed design, the gene‐tagging “recaptures” would subsequently be obtained by genotyping a random sample of 3‐year‐olds from the surface fishery catch in the GAB the following year, and then, a Lincoln–Petersen estimator (see, e.g., Seber, 1973) would be used to estimate the absolute abundance of the cohort at age 2. This means the gene‐tagging‐based estimators of cohort abundance will be negatively biased if some juveniles never enter the GAB (Preece et al., 2015). Bias of the gene‐tagging‐based estimators could also arise from other types of incomplete mixing.

The distinction between a juvenile population in which individuals disperse with independent migration probabilities from a common summer feeding ground in the GAB and one that consists of separate groups, some of which do not migrate to the GAB is also of interest from an evolutionary point of view. Cadrin and Secor (2009, p. 414) use the term “contingent” to refer to “a cohesive group of individuals within a population that share a common migrational pattern.” The existence of multiple contingents within a population may provide resilience to environmental changes and fishing pressure (Secor, 1999). The existence of contingents within the western population of Atlantic bluefin tuna (*T. thynnus*), northern Atlantic cod (*Gadus morhua*), Chesapeake Bay striped bass (*Morone saxatilis*), and American eels (*Anguilla rostrata*) have been suggested previously (Secor, 2015, Chapter 7). The demonstrated existence of multiple contingents within the juvenile SBT population would support the view that this trait may sometimes be important for the establishment and persistence of populations.

The remainder of the article is written in four parts. First, we provide some background information on the fisheries that have historically harvested SBT and the development of theories of juvenile migration. Secondly, we describe the major data sources that provide information on the movement and distribution of juvenile SBT. These include a range of data that have not been previously considered in detail. We use a novel hierarchical Bayesian modeling approach to assess evidence of incomplete mixing in observed tag recoveries. The new modeling approach addresses some of the limitations of methods previously used to assess mixing in SBT. We also consider major tagging studies run during the 1990s and 2000s that have been largely overlooked in terms of information on juvenile movement. We provide summaries of recoveries of tags released in disparate oceanic regions that indicate the nature of spatial structuring of juveniles and subadults more clearly than previously published work. We also critically review recent analyses of electronic archival tagging studies. We argue the proportion of juveniles that summer in the GAB cannot be inferred from archival tagging data alone. Thirdly, we propose an updated understanding of juvenile SBT migration based on simultaneous consideration of all of the information presented. We argue that the combined evidence strongly favors the hypothesis of subgroups of juveniles either side of the Indian Ocean that exhibit limited intermixing. We argue that the nature of juvenile distribution and migration in SBT is well described by the concept of contingents that has been proposed for other fish species. We suggest our summaries of recoveries from tags released in oceanic waters provide the first evidence that migration choices in juvenile SBT reflect fidelity to winter feeding grounds. Finally, in the Discussion, we propose a mechanism for the migration of juvenile SBT based on social learning. We explain how this mechanism is consistent with the collapse of the NSW SBT fishery in the 1980s and also discuss implications of our findings for future research.

## 2 HISTORICAL BACKGROUND

**Figure 2:**
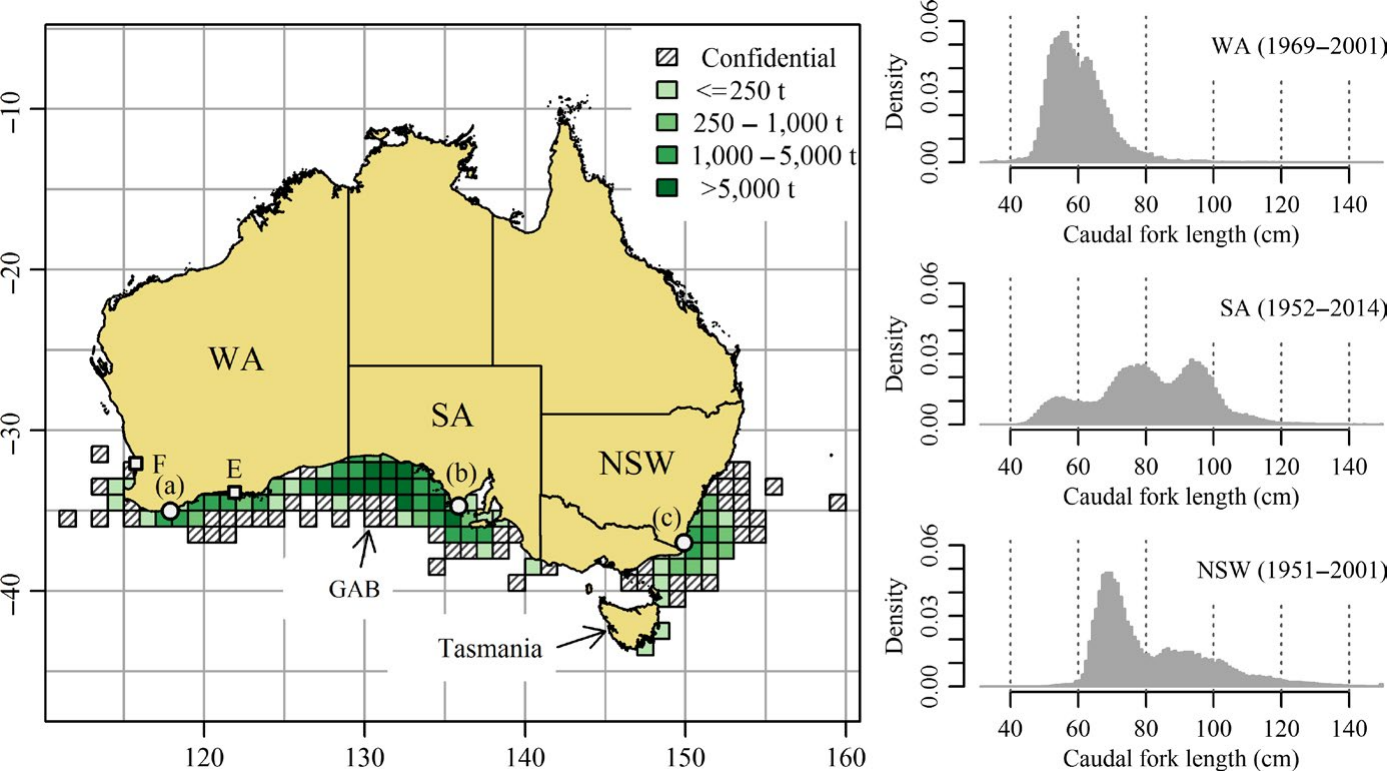
Map showing historical catch of southern bluefin tuna by 1‐degree square of latitude and longitude reported by Australian surface fisheries that were centered in (a) Albany, Western Australia, (b) Port Lincoln, South Australia, and (c) Eden, New South Wales, and (right) density histograms of the lengths of sampled catch from each fishery. The Great Australian Bight is the high catch region to the west of Port Lincoln. Locations labeled F and E are Fremantle and Esperance respectively. Catch in 1‐degree squares considered confidential if fished by fewer than three vessels in all months or <5 months fished in total. (Catch data source: Commission for the Conservation of Southern Bluefin Tuna database)

Commercial fisheries for SBT using pole and live bait were first established in the early 1950s based in Eden, New South Wales (NSW), and Port Lincoln, South Australia (SA; Figure 2). A third surface fishery developed off Albany in Western Australia (WA) in the late 1960s (Caton, McLaughlin, & Williams, 1990). The SA and NSW fisheries expanded steadily during the 1960s and 1970s. In keeping with previous literature, we refer to Australian pole and line and purse seine fisheries collectively as the Australian “surface fisheries”. Purse seining, which had been attempted earlier, was applied with greater success from the mid‐1970s. However, the abundance of juveniles on the NSW fishing grounds declined sharply in the early 1980s until the 1985 fishing season failed completely (Caton, 1991, p. 249). Individual transferable quotas were introduced in 1984 to limit the Australian commercial catch (Caton et al., 1990). The new management arrangements coupled with spatial changes in the availability of juveniles resulted in a restructure of the Australian industry, which became increasingly centered in the GAB (Campbell, Brown, & Battaglene, 2000). The present‐day surface fishery, based at Port Lincoln, uses predominantly purse seine to capture schools of juveniles that are then transferred to tuna ranches for fattening prior to harvest and export (Ellis & Kiessling, 2016).

**Figure 3:**
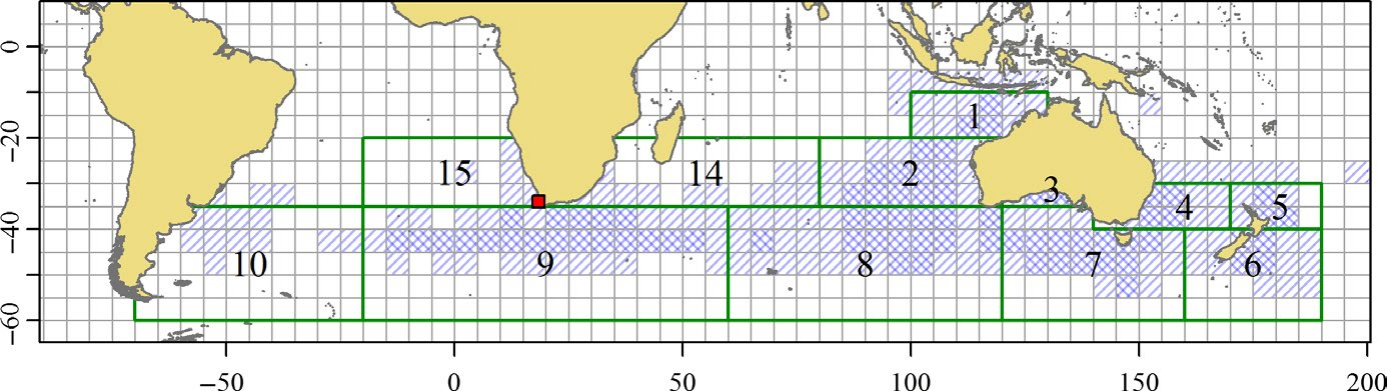
The Commission for the Conservation of Southern Bluefin Tuna (CCSBT) statistical areas bounded by the solid green lines and numbered as shown. Region in which capture of southern bluefin tuna was recorded by the Japanese longline fleet up to 1975 shown as hatched 5‐degree squares. Cross‐hatched squares indicate locations where catch to 1975 exceeded 10,000 individuals. The location of Cape Town is indicated by the red square. (Catch data source: Commission for the Conservation of Southern Bluefin Tuna database)

Japanese longline fleets began harvesting adult SBT on their spawning ground (Figure 3, Area 1) in 1952 (Shingu, 1970; Suda, 1971). Longline fishing on the spawning grounds and the staging grounds (Figure 3, Area 2), immediately south of the spawning grounds, allowed Japanese scientists to conduct important research into the timing of spawning, length at sexual maturity, fecundity, and oocyte development (Shingu, 1978). While adults were caught on the spawning grounds between August and March (Shingu, 1978), separate peaks in catch rates on the spawning grounds were regularly observed in September–October and then later in February.

Poor meat quality of spawning adults led the Japanese longliners to shift their attention to feeding grounds in the “West Wind Drift”. also called the Antarctic Circumpolar Current, about the 40°S parallel. Adult SBT with better quality meat are found on the feeding grounds of the West Wind Drift, especially in late winter (Shingu, 1978). The areas fished by the Japanese longline fleet expanded rapidly during the 1960s as new fishing grounds were discovered. Notable longline fishing grounds were identified in the southeast Indian Ocean (SEIO; Figure 3, Area 8), south of Australia (Figure 3, Area 7), in the Tasman Sea (Figure 3, Area 4), in the waters around New Zealand (Figure 3, areas 5 and 6), and south of Africa (Figure 3, Area 9), with the spatial extent of longline fishing for SBT peaking around 1970 (Shingu, 1978). The spatial distribution of early longline catch, shown in Figure 3, is likely to define essentially the entire global distribution of the species.

Large‐scale tagging studies were run during the 1960s with intermittent tagging programs during the 1970s and 1980s. More than 60,000 juveniles were tagged and released off the coasts of WA, SA, and NSW between 1959 and 1984 (Hampton, 1991). Around 12,000 of these tags were later recovered via fishery recapture. The majority of tag recoveries were obtained from recaptures by the Australian commercial surface fisheries. According to Caton (1991, p. 235–240), these recoveries showed that young SBT moved quickly east out of WA. Large numbers remained in SA for 2–4 years, but at least part of the juvenile population moved seasonally between SA and NSW. Some juveniles resided on the east coast of Australia over summer (Robins, 1963, p. 573), while movement from SA to WA appeared to be uncommon. Tags were also recovered from Japanese longline vessels. The longline recoveries of tags released from Australia were recaptured as far west as the southeast Atlantic Ocean and as far east as off eastern New Zealand, demonstrating connectivity among the individuals found in these areas.

**Figure 4:**
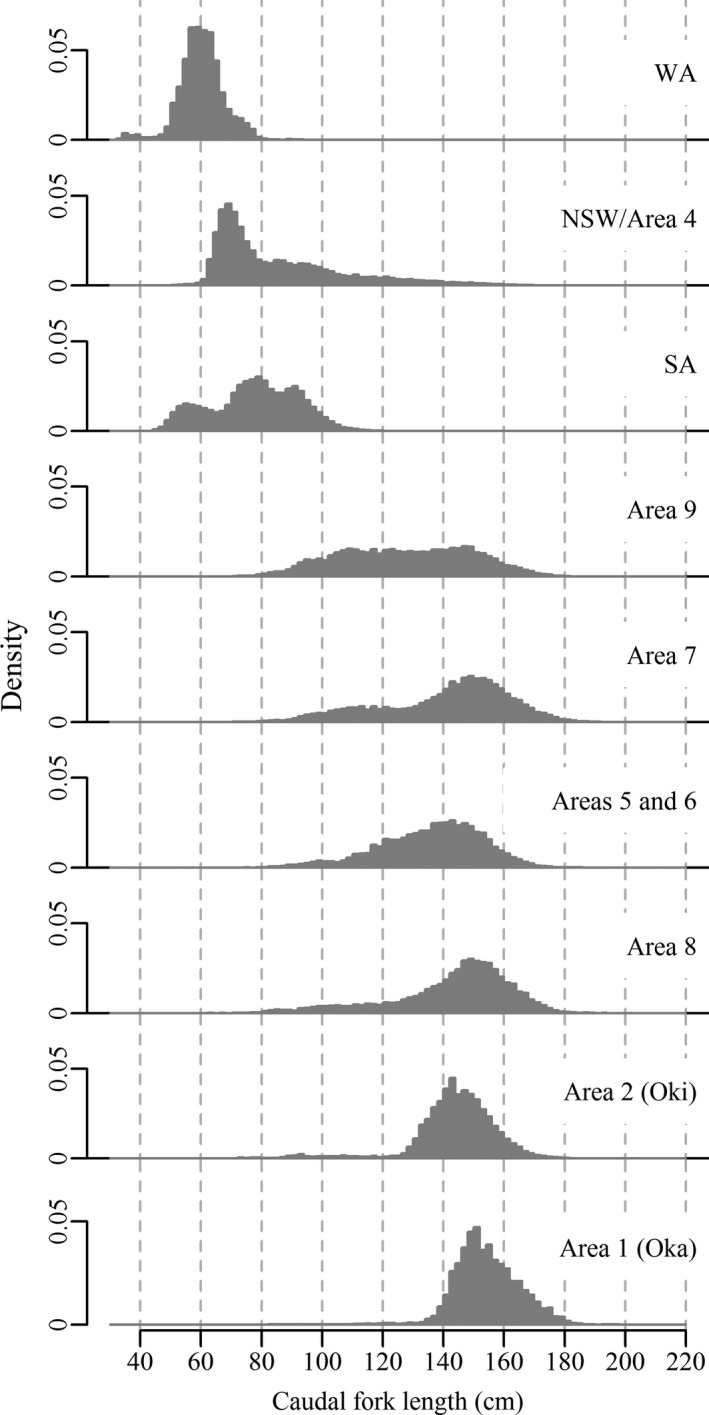
Density histograms of distribution of caudal fork lengths of southern bluefin tuna sampled from catch on various fishing grounds up to 1975 (Adapted from Shingu, 1978). Labeled regions correspond to those shown in Figures 2 and 3. (Data source: Commission for the Conservation of Southern Bluefin Tuna)

Observed differences in the age distribution of SBT catch on the different fishing grounds were central to the development of the first theories of SBT migration and movement. Shingu (1970, 1978) compared histograms of estimated ages of SBT sampled from the catch on the major fishing grounds (see Fig.S1). He arranged the histograms similarly to the length sample density histograms included in this study (Figure 4), implying an approximate ordering based upon the inferred ages of the individuals caught on each of the fishing grounds. Aside from differences in the age distributions of SBT caught on the different fishing grounds, the theories of SBT movement proposed by Shingu (1967, 1970, 1978) and Nakamura (1969) were also informed by seasonal changes in catch rates, tag recoveries, and observations of ovaries and flesh quality. The presence and movements of SBT were hypothesized to be related to observations of oceanographic quantities such as surface currents, sea surface temperature, and salinity (Shingu, 1970; p. 17). The basic understanding of migration proposed by the Japanese scientists has since been referred to as the “traditional model” of SBT migration (see, e.g., Caton, 1991; Hampton, 1989; Olson, 1980).

Murphy (1977) suggested large numbers of small SBT (of length 61–80 cm) caught south of Africa meant that many juveniles must head west without ever entering the Australian fishing grounds. He reasoned that the number of juveniles caught south of Africa indicated the juvenile population in this region might be similar in abundance to that off southern Australia. The dispersion of juveniles from Australia proposed by Murphy and Majkowski (1981) is similar to Fig.S3 provided in the Supporting information. Hampton (1989) noted average Japanese longline catch rates of 2‐ to 4‐year‐olds between 1969 and 1985 were 6.5 individuals per thousand hooks off South Africa compared with 9.4 per thousand hooks in the Tasman Sea. He suggested “the simultaneous presence of large numbers of the same cohort in fishing grounds separated by more than 10,000 km must indicate a major divergence of migratory path” (Hampton, 1989; p. 13). Importantly, in this comment, Hampton (1989) seems to imply that not only are large numbers of juveniles from the same cohort present in disparate regions, but also that the distance between these regions would be expected to inhibit their intermixing.

**Figure 5:**
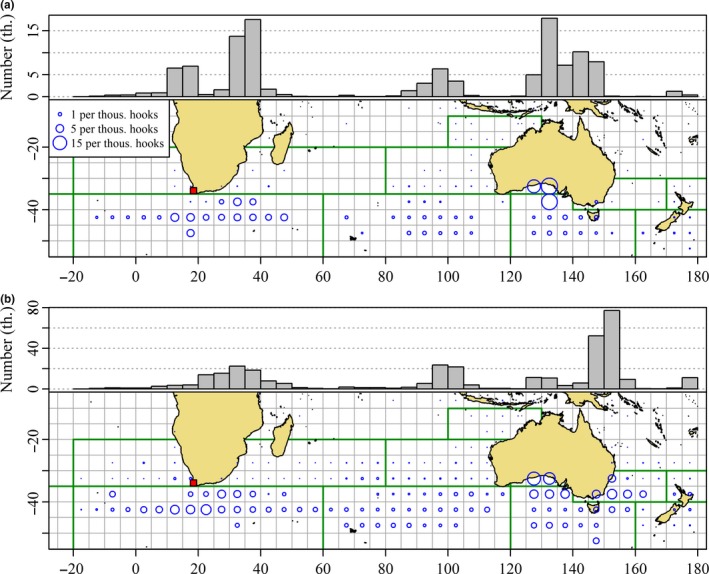
Distribution of average catch per unit effort (CPUE) of juvenile southern bluefin tuna (SBT, number of SBT 4 years old and younger per thousand hooks) reported by the Japanese longline fleet between 1967 and 1970 in (a) December–March and (b) May–August. Area of plot character proportional to average CPUE, winter and summer CPUE, is comparable. Cells with less than 50,000 hooks set are excluded. The histograms in the top margins of each panel show total reported catch of SBT 4 years of age and younger between 1967 and 1970 by 5° of longitude. Note the differences in the scales of the histograms. The location of Cape Town is indicated by the red squares. (Source: Commission for the Conservation of Southern Bluefin Tuna)

A sense of the habitat of juvenile SBT can be gauged from the spatial distribution of catch rates of juveniles by Japanese longline fleets between 1967 and 1970 (Figure 5). During this period, when the distribution of Japanese longline fishing for SBT was at its peak, juveniles were frequently caught across a broad stretch of the southeast Atlantic and southwest Indian Oceans both in summer (Figure 5a) and winter (Figure 5b).

Differences in the location of longline recaptures of tags released from the different Australian states, Murphy (1977) argued, were further evidence that the traditional model was inadequate. Hynd and Lucas (1975) showed that juvenile SBT populations tagged off WA appeared to experience much lower fishing mortality on the surface fishery grounds in NSW and SA than did populations that were tagged and released in NSW and SA. Murphy (1977) concluded it was a “near certainty” that some juveniles that were available to the WA fishery did not later become available to the SA and NSW fisheries. Murphy and Majkowski (1981, their figure 1) suggested that groups of juveniles moved away from coastal waters into the Indian Ocean at various points off the southern Australian coast (see Fig.S3).

The “alternative model” proposed by Murphy (1977) received considerable support during the 1980s and early 1990s (e.g., Hampton, 1989; Ishizuka, 1987; Murphy & Majkowski, 1981). Ishizuka (1987) summarized recoveries of tags released from the vicinities of Fremantle, Albany, and Esperance in WA (see Figure 2). The proportion of tags recovered from Esperance releases was higher than from Albany releases which was higher than from Fremantle releases. The apparent leakage of the 1‐year‐olds to the west into the Indian Ocean as the fish moved south and then east off the Western Australian coast led Ishizuka (1987) to conclude the tag recovery data were consistent with the alternative model proposed by Murphy. Some authors (e.g., Caton, 1991; Caton et al., 1990; Hunter et al., 1986) merely noted that the proportion of juveniles that headed west, bypassing the Australian fisheries, was unknown.

## 3 EVIDENCE OF SEPARATE JUVENILE SUBGROUPS

### 3.1 Catch at age of longline fleets

**Figure 6:**
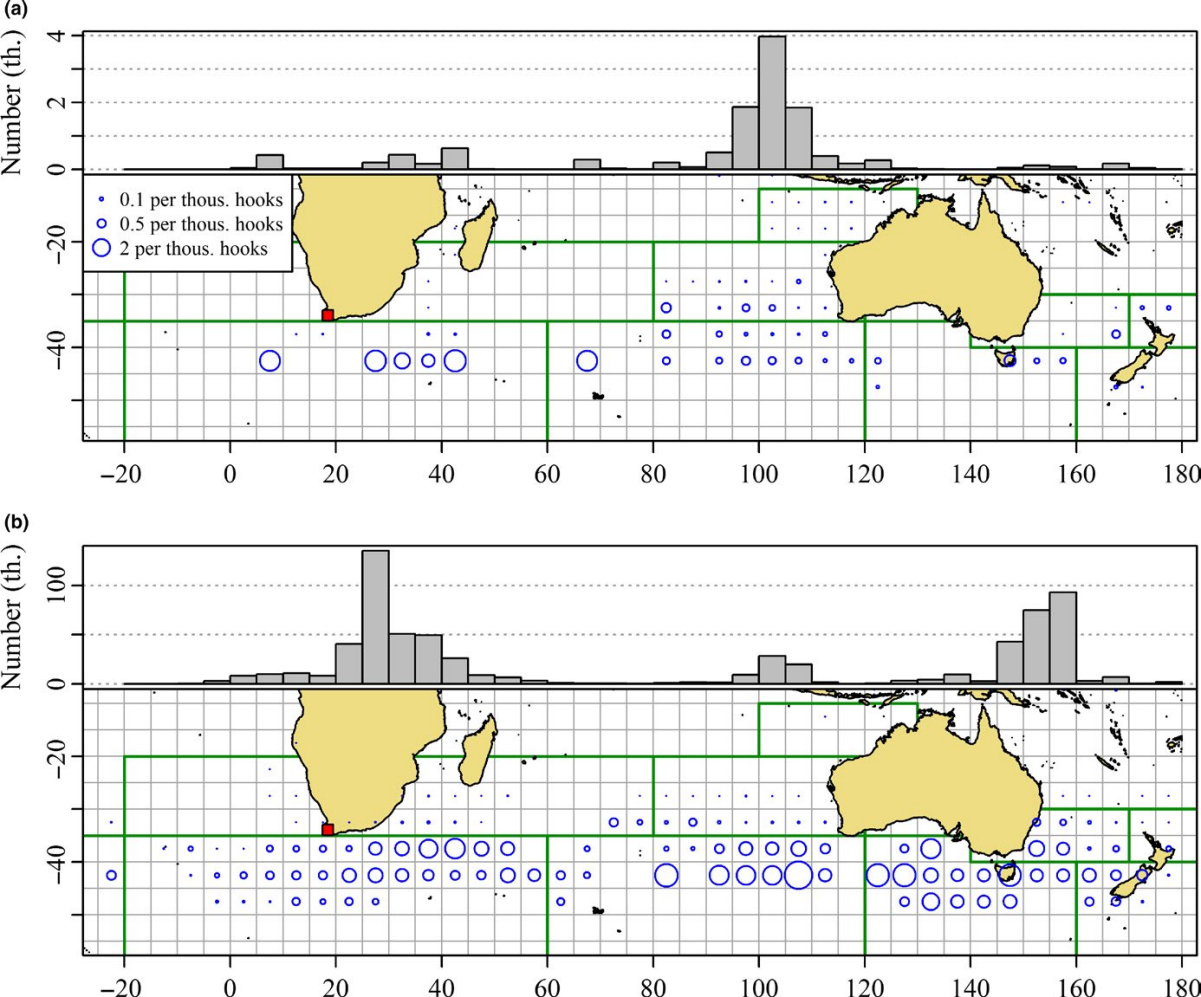
Distribution of average catch per unit effort (CPUE) of juvenile southern bluefin tuna (SBT, number of SBT 4 years old and under per thousand hooks) by the Japanese longline fleet between 1990 and 2014 in (a) December–March and (b) May–August. Area of plot character proportional to CPUE, winter and summer CPUE, is comparable. Cells with less than 50,000 hooks set are excluded. The histograms in the top margins of each panel show total reported catch of SBT 4 years of age and younger between 1990 and 2014 by 5° of longitude. Note the differences in the scales of the histograms. The location of Cape Town is indicated by the red squares. (Source: Commission for the Conservation of Southern Bluefin Tuna)

In this section, we examine catch‐at‐age data from the CPUE_INPUTS table of the CCSBT database. We assume the age assignments in the database are unbiased. Determination of catch‐ at ‐age in SBT is described by Kolody, Eveson, and Hillary (2016). Catch rates of SBT 4 years and younger reported by Japanese longline fleets from 1990 to 2014 demonstrate that juvenile abundance off southern Africa in winter is likely to be similar or perhaps larger than abundance in the SEIO (Figure 6b). Similarly, total catches of juveniles between 1990 and 2014 demonstrate that a major proportion of the total catch of juveniles by the Japanese longline fleet during winter has occurred in the southwestern Indian Ocean and southeastern Atlantic Ocean (Figure 6b). The 5‐degree band of longitude accounting for the highest number of juveniles captured by the Japanese longline fleet is that between 25°E and 30°E, south of the African continent (Figure 6b).

Of course, the relationship between catch and abundance is completely confounded by fishing mortality (see, e.g., Quinn & Deriso, 1999, chapter 1). The Australian purse seine fleet catches more juveniles in the GAB than the entire Japanese catch. This occurs because the Australian fleet has been allocated a larger proportion of the quota than the Japanese fleet by the CCSBT in recent years and because it targets smaller individuals. The presence of large numbers of juveniles in the GAB in summer is beyond doubt and has been fundamental to all models of juvenile SBT migration. By contrast, the catch of juveniles south of Africa is consequential because this is potentially inconsistent with the contention that there is unlikely to be a large population of juveniles off South Africa in summer.

Inferences from Figures 5 and 6 about spatial trends in relative juvenile abundance should be based on catch rates rather than total catch. Even so the fishery dependent nature of catch‐at‐age data does not permit precise inferences about the proportion of juveniles off southern Africa. Moreover, this proportion is unlikely to be constant. It is sufficient for our purposes to conclude it is unlikely to be a negligible proportion of the total population of juveniles. We note at this point that the distribution of SBT 4 years and under extends west of Cape Town (longitude 18°E, Figure 6). The frequent capture of SBT <5 years of age off southern Africa is evidence that juveniles are commonly present in this area. Therefore, any acceptable model of SBT migration must accommodate the presence of juvenile SBT south of Africa.

**Figure 7:**
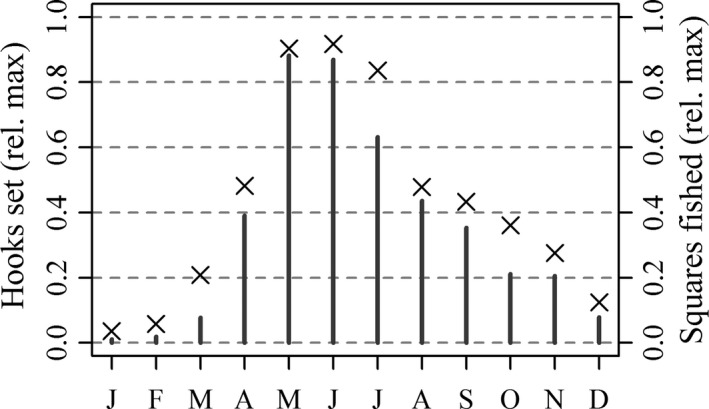
Longline hooks set (vertical bars) and 5‐degree squares fished (crosses) by the Japanese longline fleet by month in Commission for the Conservation of Southern Bluefin Tuna (CCSBT) statistical areas 4–9 (see Figure 2), expressed as a proportion of the annual monthly maximum averaged between 1990 and 2014. Squares with fewer than 10,000 hooks set were excluded for the 5‐degree square comparison. (Source: CCSBT)

In recent years, there has been minimal longline catch of juvenile SBT during summer (Figure 6a) compared with historic levels (Figure 5a). Although this would be consistent with the view that most juveniles off South Africa in winter now migrate to the GAB in summer, the Japanese longline fleet is less active during summer, because adult SBT leave the temperate feeding grounds to migrate to the spawning ground (Figure 3, Area 1). The exodus of large SBT makes fishing south of Africa in summer less attractive to the longline fishers (Warashina, Nishikawa, Tsuji, Ishizuka, & Suzuki, 1989). Minimal fishing during summer is evidenced by negligible catches of juveniles in summer even where CPUE is relatively high (Figure 6a). A lesser interest of longline fishers in small SBT also explains a tendency, beginning perhaps in the 1990s, to release individuals captured at less than about 25 kg or 5 years of age (Butterworth, Ianelli, & Hilborn, 2003). The Japanese fishers target neither spawning adults nor juvenile SBT presumably because it is not economically rational for them to target low‐value individuals in a quota limited fishery. Instead, they target subadults and adults with high‐fat content available on the feeding grounds of the West Wind Drift over the austral winter. As we show in Figure 7, Japanese longline fishing targeted at SBT is much reduced in the summer months over the main longline fishing grounds. Nevertheless, average juvenile catch rates in squares fished south of Africa over summer are comparable with or higher than those observed during winter (Figure 6). As mentioned earlier, catch of juveniles south of Africa during summer was widespread when Japanese longline fishing at this time of year was more extensive (Figure 5a).

Further evidence of the presence of juveniles south of Africa during summer comes from the location of voluntary spatial closures introduced by the Japanese longline fleet. The spatial closures were introduced in 1971 by the Japanese longline fleet to protect young SBT and the spawning stock. These included a closure aimed at protecting juveniles located south of Africa, extending between longitudes 15°E and 35°E, and between latitudes 38°S and 45°S. The designated region south of Africa was closed seasonally over most of spring and summer from the beginning of October to the end of January (Caton et al., 1990; Shingu, 1978; Warashina & Hisada, 1974).

**Figure 8:**
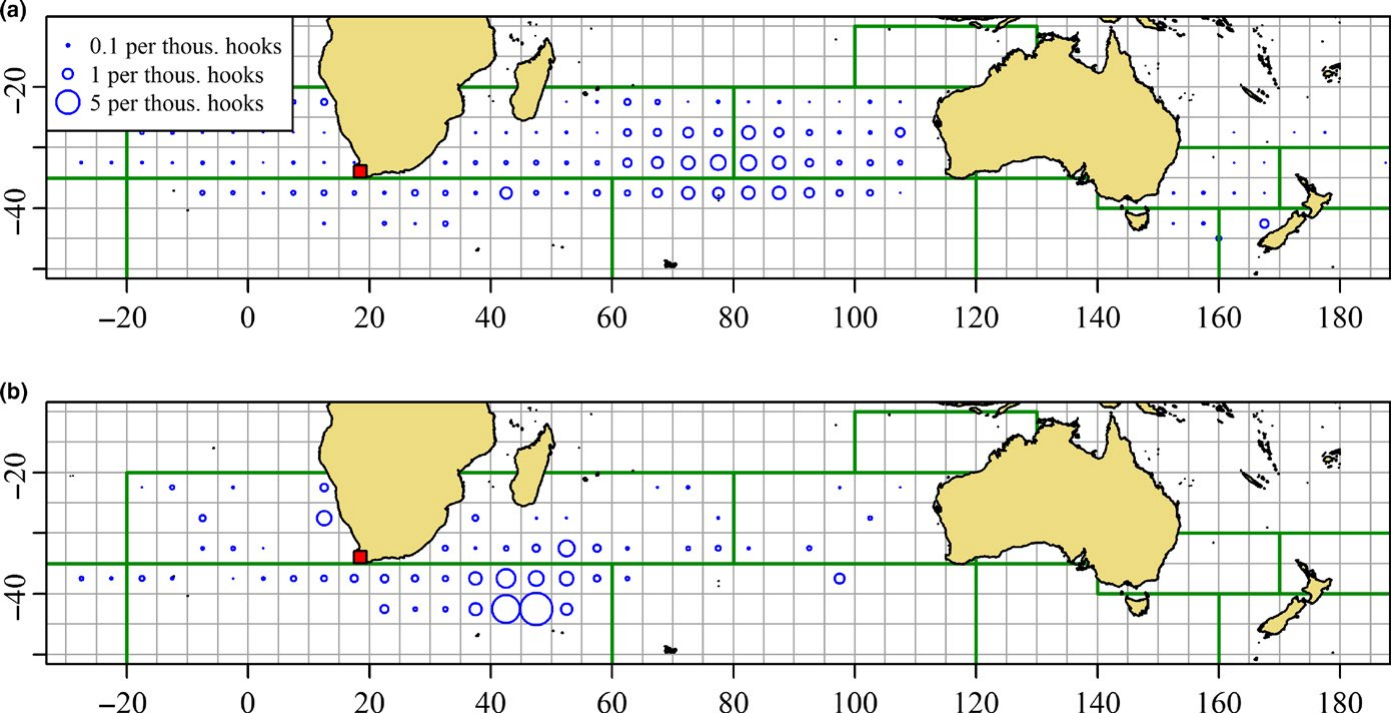
Nominal catch per unit effort (CPUE) of southern bluefin tuna by the Taiwanese fleet (all ages) aggregated to 5‐degree square during May to October (top) and November to February (bottom). Area of plot character proportional to CPUE, winter and summer CPUE, is comparable. (Source: CCSBT)

Information describing the catch of SBT by Taiwanese fleets provides further evidence of juvenile SBT outside the GAB during summer. Historically, catch of SBT has been dominated by Japan and Australia with much smaller catches reported by New Zealand fleets. The catch of The Fishing Entity of Taiwan (hereafter Taiwan), Korea, and Indonesia all increased during the 1990s (Farley, Davis, Gunn, Clear, & Preece, 2007), and all three countries are now members of the Commission for the Conservation of Southern Bluefin Tuna (CCSBT). The catch of the Taiwanese fleet is of particular interest because the Taiwanese longline fishers are known to catch smaller, and therefore younger, SBT than the Japanese and Korean fleets (Farley et al., 2007; Shiao, Chang, Lin, & Tzeng, 2008). Catch of SBT by Taiwanese flagged vessels occurs mostly in two seasons (Shiao et al., 2008). The main fishing season runs from May to October (Figure 8a) in the central Indian Ocean (CIO), while a second, less substantial fishing season runs from November to February in the western Indian Ocean, southeast of South Africa (Figure 8b).

**Figure 9:**
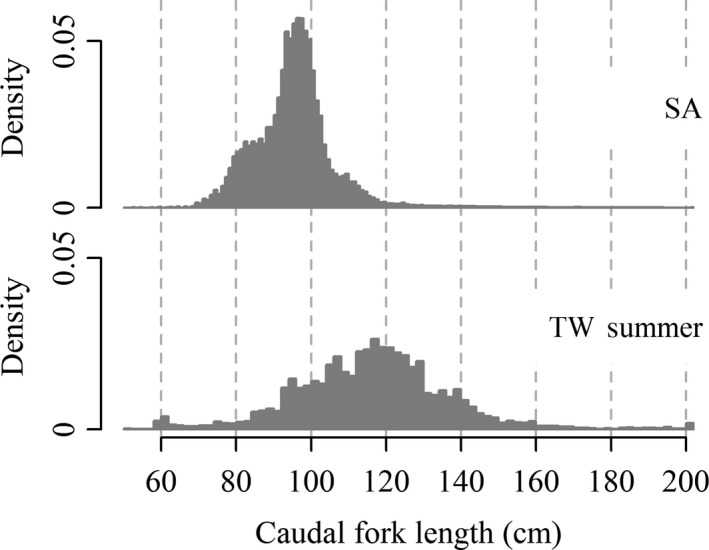
Lengths of southern bluefin tuna sampled from the catch of the surface fishery off South Australia (top) and by Taiwanese fleets in the summer fishery off southern Africa (bottom) since 1993. The Taiwanese length frequency data are censored at 50 cm and 202 cm on the Commission for the Conservation of Southern Bluefin Tuna (CCSBT) database. (Source: Commission for the Conservation of Southern Bluefin Tuna)

Gunn et al. (2003) described interviews with skippers and crew of Taiwanese longline vessels that were operating in the Indian Ocean. Catch of SBT by the Taiwanese fleet in the CIO is thought to be largely a byproduct of vessels targeting albacore (*T. alalunga*). Three‐year‐olds were the most frequent age class of SBT captured by this fishery. Catch of SBT on the summer Taiwanese fishing ground was dominated by 2‐ to 4‐year‐olds (Gunn et al., 2003, p. 47‐48). The Taiwanese fishers described observations of schools of SBT in the western Indian Ocean both in summer and in winter (Gunn et al., 2003, p. 49) and suggested that longline catch of SBT in the area was dependent upon a line of hooks being encountered by a school. The Taiwanese fishers also mention “South African fish” which were described as small SBT found close to South Africa, as opposed to Australia, during summer (Gunn et al., 2003, p. 45). Although the Taiwanese fleet catches a wider range of sizes of SBT than the SA purse seine fishery, there is considerable overlap in the length frequency distributions of their catch (Figure 9).

Overall, the catch data are consistent with the contention of Farley et al. (2007, p. 151) who, based on their own analysis of catch‐at‐age data, stated “Occurrence of 2‐ to 4‐year‐old SBT either side of the Indian Ocean confirms juveniles are not restricted to the southern coastal waters of Australia and a divergent migration path must exist possibly near the southwest coast of Australia”.

### 3.2 Recoveries of conventional tags released off the Australian coast

We provide a summary of juvenile SBT tagged and released off southern Australia by decade, tagging location and age in Table 1. The CCSBT and CSIRO databases include estimates of the age‐at‐tagging of each tagged fish. Tagging studies run before 1990 are described in Caton (1991), and studies run during the 1990s and 2000s are as described by Polacheck et al. (2006). Following Murphy (1977), we consider three separate tagging locations, WA (west of 129°E), SA (between 129°E and 141°E), and EA (east of 141°E). The first two tagging locations coincide with Australian state boundaries (Figure 2). Releases from EA were predominantly made off the state of NSW, but also include some releases made further south off the island state of Tasmania. For the purpose of classifying the recapture locations of tagged SBT, the full set of CCSBT statistical regions shown in Figure 3 is more detailed than required, so we define three longline fishing regions, Western LL (west of the 60°E meridian), Central LL (between the 60°E and 120°E meridians), and Eastern LL (east of 120°E). These regions (see Figure 11) are the same as those used by Caton (1991, his table 23) to classify longline recovery locations of conventional tags released before 1990.

**Table 1:** Summary of recoveries of southern bluefin tuna tagged off the southern and eastern coasts of Australia cross‐classified by decade and tagging location

	Releases	Recoveries		Recovered by longline		
		Surface	Longline	Western LL	Central LL	Eastern LL
Releases of 1‐year‐olds 1960s						
WA	21,909	557 {0.025}	127 {0.006}	48 (0.38)	20 (0.16)	59 (0.46)
SA	2,131	229 {0.11}	18 {0.008}	10 (0.56)	1 (0.06)	7 (0.39)
EA	9,653	2,956 {0.31}	56 {0.006}	7 (0.13)	3 (0.05)	46 (0.82)
Releases of 1‐year‐olds 1970s and 1980s						
WA	9,812	1,592 {0.16}	33 {0.003}	13 (0.39)	11 (0.33)	9 (0.27)
SA	2,756	831 {0.30}	17 {0.006}	6 (0.35)	4 (0.24)	7 (0.41)
EA	733	76 {0.10}	8 {0.011}	0 (0.00)	0 (0.00)	8 (1.00)
Releases of 1‐year‐olds 1990s						
WA	26,601	2039 {0.076}	649 {0.024}	110 (0.17)	288 (0.44)	251 (0.39)
SA	3,065	221 {0.072}	100 {0.032}	12 (0.12)	25 (0.25)	63 (0.63)
Releases of 1‐year‐olds 2000s						
WA	30,656	1,037 {0.034}	197 {0.006}	49 (0.25)	118 (0.60)	30 (0.15)
SA	1,390	178 {0.13}	27 {0.019}	12 (0.44)	15 (0.56)	0 (0.00)
Releases of 2+ year olds 1960s						
WA	564	13 {0.023}	1 {0.002}	0 (0.00)	0 (0.00)	1 (1.00)
SA	5,837	134 {0.023}	126 {0.022}	21 (0.17)	31 (0.25)	74 (0.59)
EA	1,466	49 {0.033}	22 {0.015}	0 (0.00)	2 (0.09)	20 (0.91)
Releases of 2+ year olds 1970s and 1980s						
WA	538	95 {0.18}	6 {0.011}	2 (0.33)	2 (0.33)	2 (0.33)
SA	716	185 {0.26}	15 {0.021}	9 (0.60)	3 (0.20)	3 (0.20)
EA	19	5 {0.26}	0 {0.00}	0	0	0
Releases of 2+ year olds 1990s						
WA	3,481	242 {0.070}	125 {0.036}	29 (0.23)	43 (0.34)	53 (0.42)
SA	32,897	2,275 {0.069}	1,429 {0.043}	197 (0.14)	403 (0.28)	829 (0.58)
Releases of 2+ year olds 2000s						
WA	10,992	1,629 {0.15}	316 {0.029}	76 (0.24)	223 (0.71)	17 (0.05)
SA	36,414	6,066 {0.17}	548 {0.015}	99 (0.18)	303 (0.55)	146 (0.27)

Tag release locations are WA, Western Australia; SA, South Australia; EA, Eastern Australia. Numbers in braces are proportions of releases recovered from the Australian surface fishery and combined longline fisheries. Numbers in parentheses are proportions of longline recaptures by tagging group. Tags recaptured in the same season as tagging have been excluded.

As higher proportions of recoveries would be expected to occur immediately after release in states where surface fishery fleets are operating, we excluded recoveries that occurred in the surface fishing season the tag was released. Consistent with the definition of the Australian surface fishery season used by Polacheck et al. (2006), we define tagging year as the 12‐month period beginning on 1 November. If juveniles are mixing thoroughly and are all resident in the GAB over summer, then the tags released from different locations at the same time should be recovered with similar probabilities in subsequent fishing seasons.

Recoveries of conventional tags released from southern Australia (WA, SA, and EA) are suggestive of incomplete juvenile mixing in at least two respects. Firstly, the proportions of tags released as 1‐year‐olds that are recovered by the Australian surface fishery tend to differ among tagging locations. Secondly, the spatial distributions of longline recoveries also differ by tagging location. Basson et al. (2012, their table 8.1) highlight a marked difference in the proportion of recoveries of 1‐year‐olds tagged off WA in the 2000s compared with those tagged off SA during the same period. During the 2000s, ~13% of 1‐year‐olds tagged and released from SA were recovered from the Australian surface fishery compared with only about 3% from WA (Table 1). It can be seen from Table 1 that this anomaly is not unique to the 2000s. Higher proportions of 1‐year‐olds tagged in SA (11% and 30%) were also recovered from the surface fishery during the 1960s and the 1970s–1980s than 1‐year‐olds tagged in WA (3% and 16%). Only in the 1990s were the proportions similar.

Tagging off EA occurred mostly during the 1960s. At this time, a live bait and pole surface fishery operated off Eden in NSW. The proportion of individuals tagged as 1‐year‐olds off EA that were recaptured by the surface fishery during the 1960s was very much higher than that of 1‐year‐olds tagged off WA and SA (Table 1). The suggestion here is that the 1‐year‐olds off EA during the 1960s did not fully mix with the SA group nor with the WA group. The proportion of tags released as 2‐year‐olds in any given decade that were recovered by the surface fishery are more similar between tagging states (Table 1).

We argue that Table 1 strongly suggests incomplete mixing among juveniles present at different locations off the southern and eastern Australian coast. However, some caution is required when interpreting these data. Individual cohorts were tagged and released from each state in different ratios, and the fates of members of the same cohort would not be expected to be independent.

### 3.3 Proportions of released tags recovered by surface fisheries

The proportion of a particular group of juveniles that migrated to the GAB cannot be estimated from simple summaries of tag releases and recoveries without additional data on natural mortality rates as well as surface fishery harvest rates and reporting rates. Instead, we examine the tag recovery data for evidence of incomplete mixing of individual cohorts. We show in the Appendix that, in the case of complete mixing, the subgroups of the same cohort tagged at age one can be expected to be recovered by the surface fishery in the same proportions irrespective of the harvest rate or reporting rate of the surface fishery. In this way, evidence of differences in the probability of surface fishery recovery between the two subgroups is shown to be evidence of differences between the groups in probabilities of presence on the surface fishery grounds over time.

We fit statistical models to estimate the probabilities of recovery separately by cohort. First, we model the probability a 1‐year‐old tagged off each of the three tagging locations off southern and eastern Australia is recovered by the Australian surface fishery in a subsequent fishing season.

Let π_*ly*_ denote the probability that an SBT in location l∈{WA,SA,EA} and tagged but not recovered in year *y,* is recovered by the Australian surface fishery in a subsequent year. Let *T*_*ly*_ *be* the number of 1‐year‐olds tagged and released from tagging location *l* in year *y* that are not recovered in the same year and let *S*_*ly*_ be the number of individuals from this group later recovered from the surface fishery. We assume that the observed *S*_*ly*_ are realizations of a binomial random variable with probability π_*ly*_. The numbers of surface fishery recoveries of tags released between 1959 and 2007 are modeled as:

(1) $$\begin{array}{cc} & S_{ly}∼\text{Binomial}\left(T_{ly},\;\pi_{ly}\right),\\ & \text{logit}\left(\pi_{ly}\right)\text{= log}\left(\frac{\pi_{ly}}{1\;-\;\pi_{ly}}\right)=\beta_{l}+\delta_{ly},\\ & \delta_{ly}=\phi_{l}\times \delta_{l\left(y-1\right)}+\varepsilon_{ly},\\ & \varepsilon_{ly}∼N\left(0,\sigma_{l}^{2}\right). \end{array}$$

The terms β_*l*_ define average annual probabilities on the logit scale of surface fishery recovery of tags released from locations *l* between 1959 and 2007. Separate year effects, δ_*ly*_, are defined for each tagging location. The tagging location‐specific year effects are each assumed to follow an AR(1) autoregressive process. The full set of δ_*ly*_ corresponding to each of the tagging locations is constrained to sum to zero so that the β_*l*_ are well specified. A Bayesian approach to model fitting was applied using the Stan software package (Stan Development Team 2013) with priors:

$$\begin{array}{cc} & \beta_{l}∼N\left(\text{mean}=0,\;SD=10\;\right),\\ & \phi_{l}∼N\left(\text{mean}=0,\;SD=2\;\right),\\ & \sigma_{l}∼\text{Half‐Cauchy}\left(\text{scale}=5\;\right). \end{array}$$

We base posterior inference upon 60,000 Hamiltonian Monte Carlo (HMC) samples generated from three HMC chains each consisting of 105,000 iterations, discarding the first 5,000 as burn‐in and retaining every fifth iteration thereafter. This sampling procedure gave posterior samples that varied in estimated effective length (information content after accounting for autocorrelation expressed in terms of equivalent independent samples) between parameters, but which exceeded 2,000 in all cases. The estimated potential scale reduction factor (see, e.g., Gelman & Rubin, 1992) HMC summary statistics output by Stan were given as unity in all cases suggesting that the sample had converged to the true posterior distribution. Posterior parameter summaries and posterior predictive model diagnostics are provided in the Supporting information.

**Figure 10:**
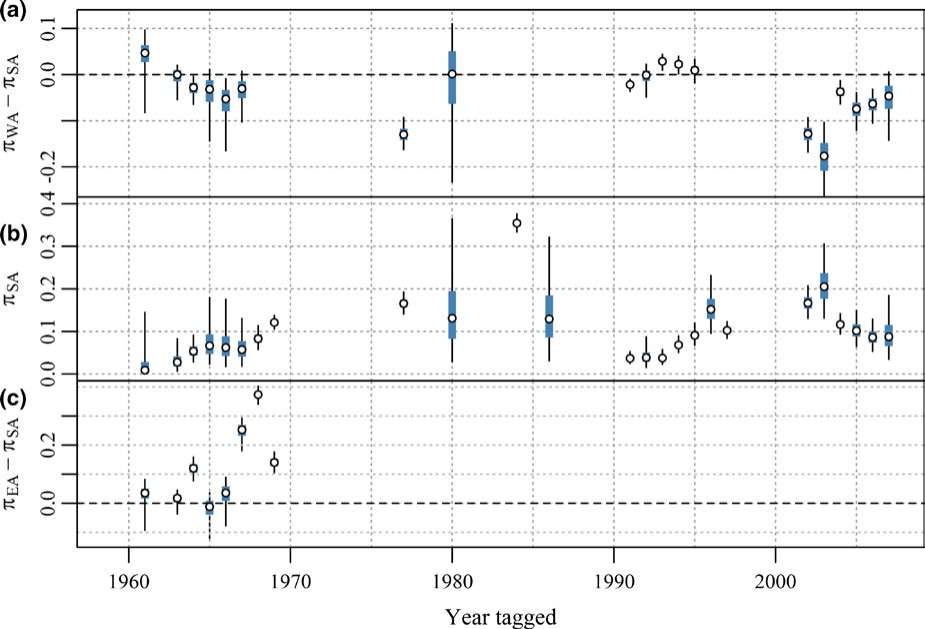
Posterior estimates of (a) probability of surface fishery recovery of 1‐year‐olds tagged in Western Australia minus probability of recovery of 1‐year‐olds tagged in South Australia (SA), (b) probability of surface fishery recovery of 1‐year‐olds tagged in SA, and (c) probability of surface fishery recovery of 1‐year‐olds tagged in Eastern Australia (EA) minus probability of recovery of 1‐year‐olds tagged in SA. The open circles represent posterior medians, the boxes represent 50% credible intervals, and the thin vertical bars represent 95% credible intervals

The fitted model suggests that in most years, the probability of recovery by the surface fishery of 1‐year‐olds tagged and released in WA was likely to have been lower than for 1‐year‐olds tagged and released from SA (i.e., π_WA_ − π_SA_ < 0), although this discrepancy is not evident in tags released during the 1990s (see Figure 10a). The difference in probability of surface fishery recovery between tags released in EA and SA was most pronounced in the latter half of the 1960s (Figure 10c). Overall, there is good evidence that 1‐year‐olds off WA during the late 1960s and 2000s were less likely to be captured by Australian surface fisheries in subsequent years than 1‐year‐olds off SA over the same intervals. We include in the Supporting information details of an alternative model for surface fishery recoveries that was fitted. The alternative model was equivalent to (1) except it assumed 1‐year‐olds released in a given year had the same surface fishery recovery probability irrespective of tagging location. Diagnostics included in the Supporting information show there is effectively zero probability that that the observed differences in proportions of surface fishery recoveries among tagging locations could have occurred if the reduced model was correct. Although the diagnostics are model specific, the extreme inadequacy of the reduced model suggests 1‐year‐olds from the same cohort in different locations off southern Australia have different probabilities of surface fishery recovery in subsequent years and therefore do not completely mix.

### 3.4 Distribution of longline recoveries by cohort

Murphy (1977) argued that if juveniles mixed off southern Australia and dispersed randomly to the high seas feeding grounds, longline recoveries should have the same distribution irrespective of where in Australia the juveniles were tagged. Considering the distribution of longline recoveries in this way is helpful because it means the problem of differences in juvenile mortality among tagging locations is largely overcome.

It is well known in fisheries science that the spatial distribution of tag recoveries over any interval will be different, in general, from the spatial distribution of tagged fish over the same interval, because of possible spatial variability in survival, fishing mortality, and tag reporting rates (see, e.g., Hilborn, 1990). Based on comments we have received from reviewers, it would seem that there is a possibly common misconception among fisheries scientists that spatial variability in these same quantities can also explain differences in the spatial distributions of tag recoveries among subgroups from the same cohort. If the subgroups are mixing thoroughly, then almost by definition they are experiencing the same environment. Therefore, unless there are phenotypical differences between the subgroups or any age differences among the subgroups cannot be reasonably ignored (remembering we refer to subgroups from the same cohort), in the case of complete mixing, the subgroups would be expected to grow at the same rate, to experience the same levels of natural and fishing mortality and the same rates of tag reporting continuously across their distribution. As we show in the Appendix, this means that statistically significant differences in the spatial distribution of tag recoveries among subgroups of a cohort over a common interval can reasonably be assumed to provide evidence of incomplete mixing. The test for complete mixing described by Latour, Hoenig, Olney, and Pollock (2001) and the “CUSTARD” test described by Kolody and Hoyle (2015) both rely upon this assumption. Inferences on fish movement can also be made by comparing recovery locations from different release sites and conditioning on subsets of recovered tags (McGarvey & Feenstra, 2002).

**Figure 11:**
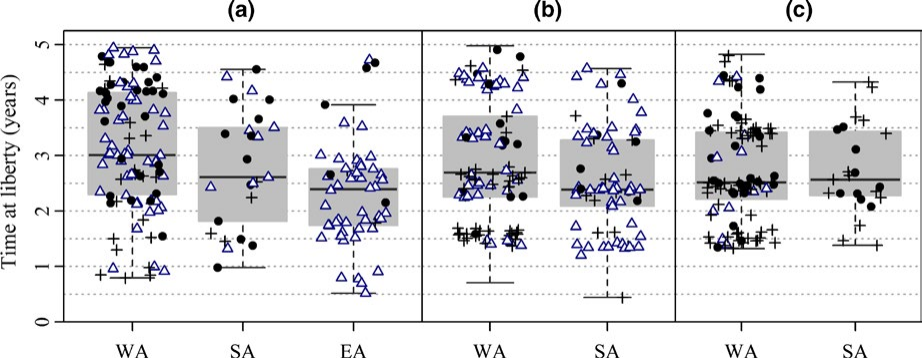
Boxplots of times‐at‐liberty by tagging state of longline recoveries of southern bluefin tuna tagged at <2 years of age and released off southern Australia (a) 1959–1984, (b) 1990–1997, and (c) 2001–2007. Times‐at‐liberty have been truncated at 5 years (with added noise) and recoveries with times‐at‐liberty <90 days excluded. Individual times‐at‐liberty are plotted over the corresponding boxplots to provide greater information about the distribution of times‐at‐liberty by tagging state. Closed black circles denote recoveries from Western LL, plusses denote recoveries from Central LL, and open triangles denote recoveries from Eastern LL. The points plotted for Western Australia releases comprise a random sample of 100 recoveries from these groups to avoid overcrowding the figure

Longline selectivity of 1‐year‐old SBT is very low (see, e.g., Butterworth et al., 2003). Therefore, the differences in longline recovery locations between tagging states, evident in Table 1, imply that not only did 1‐year‐olds from the different states tend to migrate to different feeding grounds, they remained on or returned to those fishing grounds until they became available to longline fleets in the different proportions observed. This is borne out by boxplots of times‐at‐liberty of recoveries of SBT tagged as 1‐year‐olds (Figure 11). Median times‐at‐liberty for all of the groups compared are well over 2 years. Times‐at‐liberty of individual recoveries are plotted over the boxplots with plot characters identifying the longline fishing ground where the individual was recaptured (Figure 11).

The plots show that differences in recapture locations among individuals released from the various tagging states persist for several years after release. Recoveries from Eastern LL accounted for a higher proportion of total longline recoveries among releases from EA before 1990 than among releases from WA and SA over the same period (Figure 11a). Similarly, recoveries from Eastern LL accounted for a higher proportion of longline recoveries among releases from SA during the 1990s compared to releases from WA over the same period (Figure 11b). In both cases, the discrepancies persist for times‐at‐liberty out to 3 or 4 years. The most obvious feature in Figure 11c is the lower proportion of longline recoveries from Eastern LL among releases from both WA and SA compared with earlier decades (Figure 11a,b). The number of recoveries from releases of 1‐year‐olds from SA during the 2000s (Figure 11c) was quite low. Overall, we conclude from Figure 11 that differences in the longline recovery locations among tags released as 1‐year‐olds from different tagging states evident in Table 1 are not explainable by short times‐at‐liberty. It is also worth highlighting the bands of longline times‐at‐liberty evident in Figure 11b,c. This is a consequence of the seasonal nature of the longline fleets mentioned earlier that, in recent years, have operated predominantly in the winter months (Figure 7). Additional summaries of recovery locations including surface fishery recovery locations and times‐at‐liberty of tags released from different tagging states before 1990 can be found in Caton (1991).

**Figure 12:**
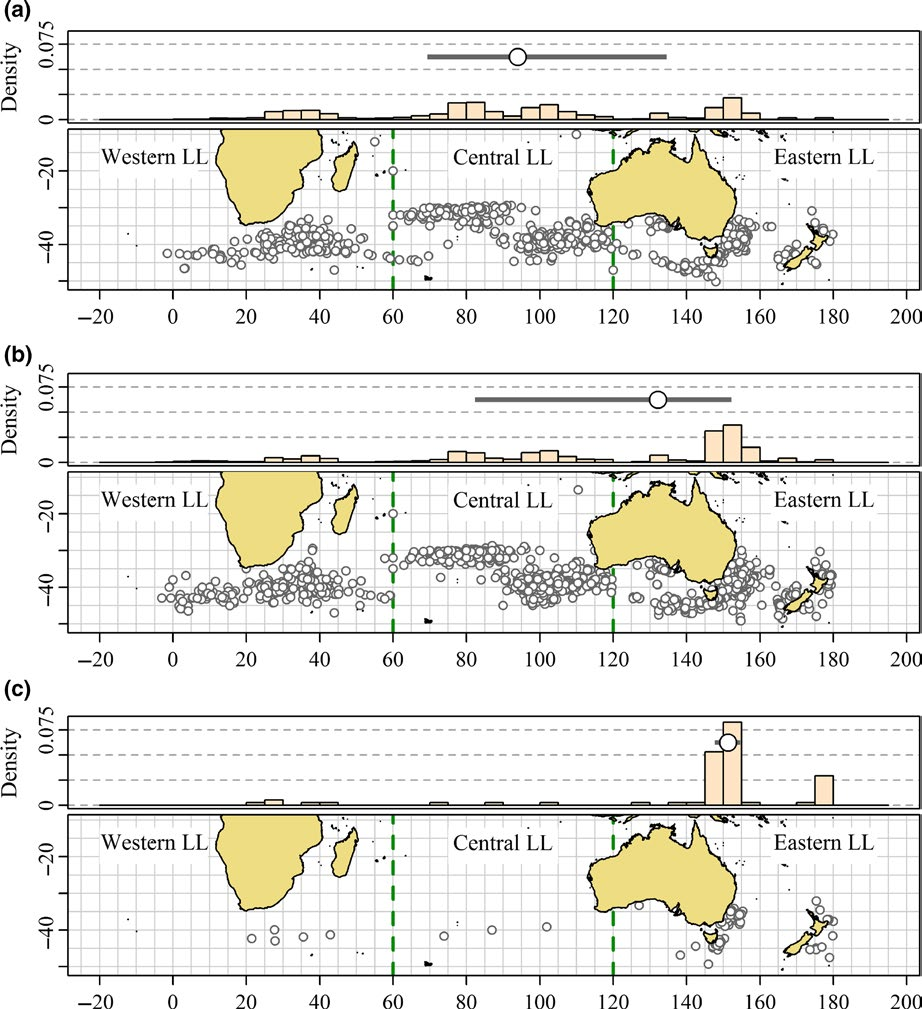
Location of longline recoveries of tags released from (a) Western Australia (WA), (b) South Australia (SA) and (c) Eastern Australia (EA) (mostly NSW) with ages‐at‐tagging <3 years. Density histograms of recovery longitude with 5‐degree bin width shown on top margins. The thick horizontal bars over the histogram panels span the interquartile range of longline recovery longitudes for each tagging location and the overplotted circles denote the median longline recovery longitudes. Recoveries with times‐at‐liberty <90 days and >5 years are excluded. (Source: Commission for the Conservation of Southern Bluefin Tuna, CSIRO)

The longline recoveries have mostly resulted from recaptures on discrete fishing grounds which can be distinguished by longitude. Comparing the distribution of longline recovery locations of juveniles tagged at 3 years of age and below by state of release (Figure 12) reveals that the differences highlighted by Murphy (1977) and Murphy and Majkowski (1981) have been maintained during the substantial tagging programs run during the 1980s, 1990s, and 2000s. The density histograms of recapture longitude show that tags released from WA recaptured by longline (Figure 12a) have occurred in greater proportion than longline recoveries of tags released from SA (Figure 12b) at almost all longitudes west of 120°E.

The analyses of longline recovery locations described earlier were qualitative. Conditioning recovery locations of individual cohorts on the total number of longline recoveries used informally by Murphy (1977) also permits more formal analyses that quantify the evidence of incomplete mixing of cohorts taking into account the numbers of recoveries observed. Latour et al. (2001) proposed a chi‐square test for incomplete mixing comparing the spatial distributions of recoveries from subgroups of individual cohorts. We consider recoveries of members of cohorts tagged at 3 years of age and below and recovered between the ages of 3 and 6 years. We show in the Appendix that the expected proportions of total longline recoveries recaptured in each longline fishing ground between the ages of three and six among subgroups of the same cohort are the same even with differences in harvest rates and reporting rates among fishing grounds. Therefore, evidence of differences in the spatial distribution of longline recoveries from subgroups of the same cohort provides evidence of incomplete mixing.

Twelve cohorts tagged in both WA and SA during the 1990s and 2000s tagging studies were recovered by longline fleets in sufficient quantities to do chi‐square tests comparing the recovery locations of recoveries released from SA and WA. The test results, provided in Table S5, indicate evidence of incomplete mixing in four of the twelve cohorts. In all four cases, significantly higher proportions of longline recoveries from WA were recaptured in Western LL and lower proportions recaptured in Eastern LL than would be expected if the cohorts were well mixed.

Arguably a limitation of the chi‐square test for nonmixing (Latour et al., 2001) is that the test will have low power except for individual cohorts with large numbers of recoveries. As an alternative, we use a hierarchical Bayesian modeling approach that allows cohort‐specific estimates of longline recovery location while also sharing of information between cohorts. Stan (Stan Development Team 2013) does not provide inbuilt functionality for fitting multinomial models, so we equivalently model longline recovery location using two complementary binomial submodels.

This time we let *L*_*ly*_ denote the number of SBT spawned in year *y* that were tagged and released from tagging location l∈(WA,SA,EA) at 3 years of age or younger and were later recovered from a longline vessel between the age of three and six. We then denote the number of these longline recoveries recaptured east of 120°E (i.e., recovered in Eastern LL) *Z*_*ly*_. We assume that each observed *Z*_*ly*_ is the realization of a random variable with a binomial probability distribution. Longline recoveries east of 120°E, *Z*_*ly*_, are modeled as:

(2) $$\begin{array}{cc} & Z_{ly}∼\text{Binomial}\left(L_{ly},\;P_{ly_{a}}\right),\\ & \text{logit}\left(P_{ly_{a}}\right)=\log\left(\frac{P_{ly_{a}}}{1-P_{ly_{a}}}\right)=\mu_{l_{a}}+\omega_{ly_{a}},\\ & \omega_{ly_{a}}=\kappa_{a}\times \omega_{l{\left(y-1\right)}_{a}}+\varepsilon_{ly_{a}},\\ & \varepsilon_{ly_{a}}∼N\left(0,\sigma_{l_{a}}^{2}\right). \end{array}$$

The terms $\mu_{l_{a}}$ define the average cohort logit‐scale probabilities that an individual spawned between 1958 and 2006 and tagged in locations *l* is recaptured east of 120°E conditional on being recovered by longline between the ages of three and six. Separate year effects, $\omega_{ly_{a}}$, are specified for each tagging location. Each set of year effects is assumed to follow their own AR(1) process but share a common AR(1) coefficient, κ_*a*_. Models were initially fitted allowing different autoregressive coefficients for releases from each tagging location, but their posterior distributions were found to be similar.

Now let *X*_*ly*_ be the number of longline recoveries of tags released from location *l* spawned in year *y* recaptured west of 120°E (i.e., not recaptured in Eastern LL) between the ages of three and six. Then, *X*_*ly*_ = *L*_*ly*_ − *Z*_*ly*_. The number of recoveries west of 60°E (i.e., recaptured in Western LL) is denoted *W*_*ly*_. We assume *W*_*ly*_ is a binomial random variable:

(3) $$\begin{array}{cc} & W_{ly}∼\text{Binomial}\left(X_{ly},\;P_{ly_{b}}\right),\\ & \text{logit}\left(P_{ly_{b}}\right)=\log\left(\frac{P_{ly_{b}}}{1-P_{ly_{b}}}\right)=\mu_{l_{b}}+\omega_{ly_{b}},\\ & \omega_{ly_{b}}=\kappa_{b}\times \omega_{l{\left(y-1\right)}_{b}}+\varepsilon_{ly_{b}},\\ & \varepsilon_{ly_{b}}∼N\left(0,\sigma_{l_{b}}^{2}\right). \end{array}$$

The definitions of parameters with *b* subscripts in submodel (3) are analogous to corresponding parameters with *a* subscripts in submodel (2). The priors for parameters of submodels (2) and (3) are the same as those used for corresponding parameters in model (1) as outlined in the Supporting information.

Finally, we let **V**_*ly*_ be a three component vector of the number of longline recoveries of releases of juveniles spawned in year *y* and tagged and released from location *l* at 3 years of age and below that were recaptured in Western LL, Central LL, and Eastern LL, respectively. Then, **V**_*ly*_ can be described as a random variable with $V_{ly}∼\text{Multinomial}\left(L_{ly},\;P_{ly}\right),$ where the three component column vector, **P**_*ly*_, has elements which are the conditional probabilities of longline recovery in Western LL, Central LL, and Eastern LL, respectively, $P_{ly}={\left[\begin{array}{c} \left(1-P_{ly_{a}}\right)P_{ly_{b}} \end{array}\;\left(1-P_{ly_{a}}\right)\left(1-P_{ly_{b}}\right)P_{ly_{a}}\right]}^{T}$, with $P_{ly_{a}}$ as defined in submodel (2) and $P_{ly_{b}}$ as described in submodel (3). Posterior parameter summaries and model diagnostics are provided in the Supporting information.

**Figure 13:**
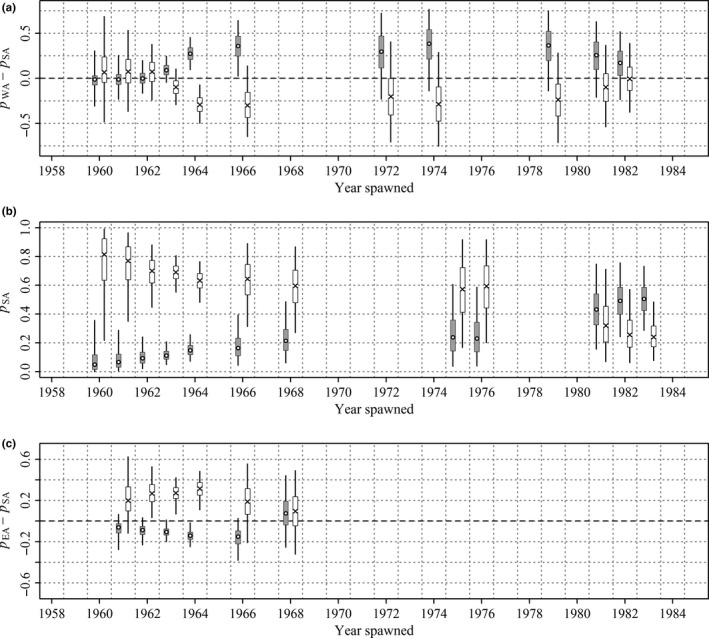
Posterior estimates of (a) probability of recapture east of 120°E meridian (crosses and white boxes) and west of 60°E (open circles and gray boxes) of tags released in Western Australia (WA) minus probabilities of tags from the same cohorts released in South Australia (SA), (b) probability of recapture east of 120°E (crosses and white boxes) and west of 60°E (open circles and gray boxes) of tags released in SA and (c) probability of recapture east of 120°E (crosses and white boxes) and west of 60°E (open circles and gray boxes) of tags released in Eastern Australia minus probabilities of tags from the same cohorts released in SA. All probabilities are conditioned on longline recovery. Cohorts spawned before 1985. The plotted points are posterior medians, boxes represent 50% credible intervals, and thin vertical bars 95% credible intervals

**Figure 14:**
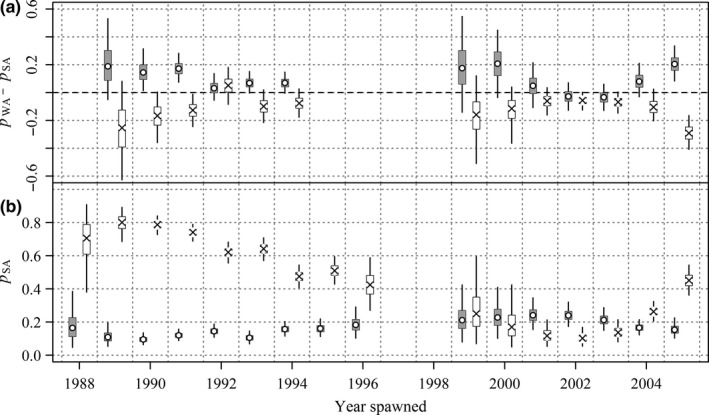
Posterior estimates of (a) probability of recapture east of 120°E meridian (crosses and white boxes) and west of 60°E (open circles and gray boxes) of tags released in Western Australia (WA) minus probabilities of tags from the same cohorts released in South Australia (SA) and (b) probability of recapture east of 120°E (crosses and white boxes) and west of 60°E (open circles and gray boxes) of tags released in SA. All probabilities are conditioned on longline recovery. Cohorts spawned since 1987. The plotted points are posterior medians, boxes represent 50% credible intervals, and thin vertical bars 95% credible intervals

Posterior probabilities of recoveries in Eastern LL and Western LL of cohorts by tagging state spawned before 1985 are plotted in Figure 13 and of cohorts spawned since 1985 in Figure 14. Posterior probabilities for releases from WA and EA are expressed as differences from releases from the same cohort from SA. There is strong evidence that cohorts spawned in the 1960s that were off EA at the times of tagging if captured by longline were more likely to be captured in Eastern LL and less likely to be captured in Western LL than members of the same cohorts off SA at the time of tagging (Figure 13c). There is some evidence that members of cohorts spawned before 1985 (Figure 13a) and strong evidence that members of cohorts spawned after 1985 (Figure 14a) off WA at the times of tagging captured by longline were more likely to be captured in Western LL than members of the same cohorts off SA at the times of tagging.

Overall, from Figures 13 and 14, we conclude that specific differences in longline recovery locations exist between SBT tagged in different Australian states, providing evidence that subgroups of individual cohorts present off different Australian states as juveniles have different future spatial distributions.

Similar to before, we fitted a reduced model that is equivalent to (2) and (3) except that it assumes juveniles spawned in a given year had the same conditional probability of longline recovery in each of the three longline regions irrespective of tagging location. Again, the diagnostics for this reduced model, provided in Supporting information, show it to be inadequate.

The observed differences in the probability of surface fishery recovery of 1‐year‐old SBT among tagging locations (Table 1, Figure 12) would seem to indicate differences between these groups in presence on the Australian surface fishery grounds in subsequent fishing seasons. However, if this were the case, all else being equal, the groups subjected to lower surface fishery harvest rates (i.e., 1‐year‐olds tagged in WA) would be expected to be recovered in greater proportion by longline fleets. Curiously, this has not been the experience with SBT. As well as being generally recovered in lower proportion by surface fishery recaptures, tag releases of 1‐year‐olds from WA were also recovered in lower proportion from longline fleets in each decade than were 1‐year‐olds tagged in SA (Table 1). This observation suggests that either a proportion of juveniles from WA migrate permanently to unfished areas or, perhaps more likely, the contingents favored by juveniles off WA experience higher rates of juvenile natural mortality. Closer analysis of the existing tag recovery data might help clarify this issue.

### 3.5 Recoveries of tags released in oceanic waters

**Figure 15:**
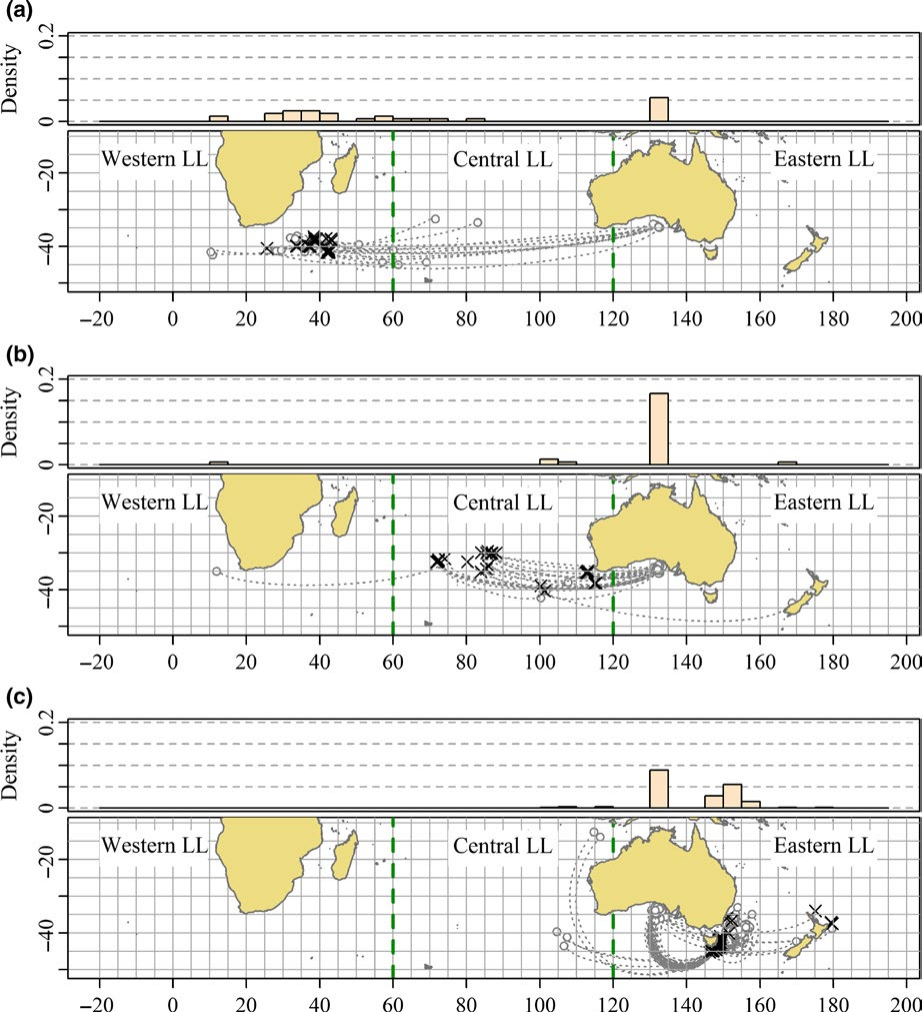
Release (crosses) and recovery (open circles) locations of tagged southern bluefin tuna (SBT) released from longline vessels in (a) Western LL region, (b) Central LL region, (c) Eastern LL region. Recoveries of SBT tagged at 4 years old and below with times‐at‐liberty exceeding 90 days are included. (Source: Commission for the Conservation of Southern Bluefin Tuna)

A limitation of early conventional tagging studies of SBT for inferring movement, as noted by Gunn and Block (2001, p. 182), was that tags were released from only a restricted portion of the overall spatial range of the population. Since this time, tagged SBT have been released from longline vessels in the Tasman Sea (Eastern LL), the SEIO (Central LL), and the western Indian Ocean (Western LL). Release and recovery locations of SBT tagged and released from longline cruises at 4 years of age and below between 1992 and 2008 are provided in Table 2 and plotted in Figure 15. These data describe both conventional and archival tag releases. To allow time for mixing, recoveries occurring 90 days or less after release were excluded. If juveniles are undertaking seasonal migrations, the probability of inter‐regional migration within 90 days of tagging should be reasonable.

**Table 2:** Locations of recoveries of conventional and archival tags released from longline cruises tagged at 4 years of age and below and with times‐at‐liberty exceeding 90 days

	Releases	Recoveries	Surface	Recovered by longline		
				West. LL	Cent. LL	East. LL
Western LL^a^	664	32 {0.05}	9 (0.28)	19 (0.59)	4 (0.13)	0 (0.0)
Central LL	402	34 {0.08}	28 (0.82)	1 (0.03)	4 (0.12)	1 (0.03)
Eastern LL	843	137 {0.16}	61 (0.45)	0 (0.0)	5 (0.04)	71 (0.52)

Numbers in braces are recoveries as proportions of tags released by release location. Numbers in parentheses are proportions of total recoveries by release location. (Source: Commission for the Conservation of Southern Bluefin Tuna.)

a An additional seven tags released from the Western LL region were recovered by longline vessels but were missing recapture location details.

Most of the oceanic tag releases from Eastern LL occurred in the 1990s, whereas most of the releases from the Western LL region occurred in the 2000s. Releases from Central LL were more balanced between these two decades. Despite the differences in the timing of the releases, it is evident from Figure 15 that individuals represented by the tags released in the Eastern LL region tended to remain east of 100°E while those represented by the tags released in the Western LL region tended to remain west of 100°E. The recoveries of conventional tags from oceanic waters provide perhaps the strongest evidence of separate contingents of juvenile SBT.

Conventional and archival tags (Table 2) tags released with juveniles tagged 4 years of age and below from oceanic cruises in Western LL were far less likely to be recovered by the surface fishery (1.4%) than were tags released from Central LL (7.0%) and Eastern LL (7.2%). This suggests that the tagged populations in Central LL and Eastern LL were perhaps five times more likely to migrate to the surface fishing grounds than were members of the population tagged in Western LL. We conclude most juveniles in Western LL did not migrate to the GAB in summer during the period covered by the oceanic tag releases in this region.

**Figure 16:**
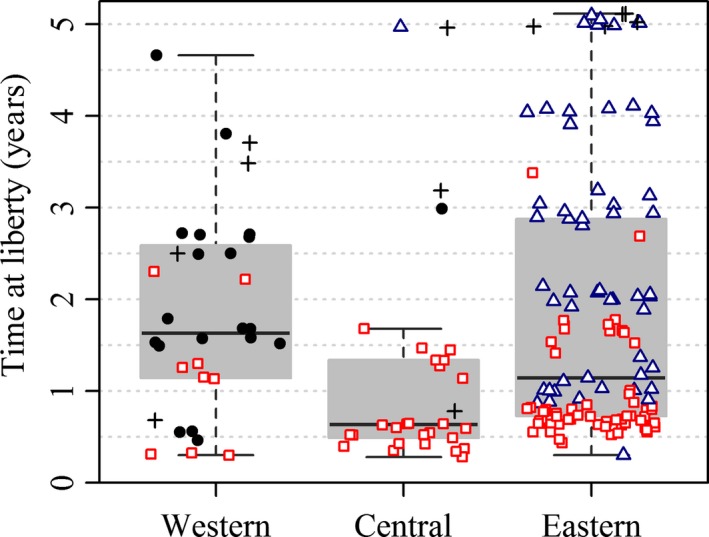
Boxplots of times‐at‐liberty by tagging location of recoveries of tagged southern bluefin tuna released from oceanic longline cruises (conventional and archival tags combined). Individual times‐at‐liberty are plotted over the corresponding boxplots to provide greater information about the distribution of times‐at‐liberty by tagging state. Closed black circles denote recoveries from Western LL, plusses denote recoveries from Central LL, open triangles denote recoveries from Eastern LL, and recoveries from the Australian surface fishery are denoted with red open squares. Recoveries occurring <90 days after release are excluded

Analyses of recovery locations of conventional and archival tags released from longline fishing grounds (Table 2, Figures 15 and 16) provide the most direct evidence of incomplete mixing in juvenile and subadult SBT. Of well over a thousand juveniles in total released from longline cruises in Central LL and Eastern LL with conventional and archival tags (see Table 2), only one archival tag has been recovered in Western LL. By contrast, 19 recoveries of archival and conventional tags released in Western LL have been recovered from a total of less than 700 tags released in Western LL. We conclude that subgroups of cohorts present in Central LL and Eastern LL at the time of tagging rarely migrated to Western LL.

Boxplots of times‐at‐liberty are shown in Figure 16 with times‐at‐liberty and recapture locations of individual recoveries shown. Where tags have been recovered from tuna farms, times‐at‐liberty have been calculated based on inferred surface fishery capture not the date of harvesting from the farm (see Chambers, Sidhu, & O'Neill, 2014). With the exception of one recovery from Eastern LL, the recoveries with shortest times‐at‐liberty from each longline tagging location were made by the surface fishery indicating the 90‐day mixing period was sufficient for these data. The boxplots reveal the recoveries mapped in Figure 15 correspond to recoveries often occurring 2 or 3 years after release and the contrasting recovery locations persist over this period.

Tags released from Eastern LL were frequently recovered from the surface fishery in the GAB between 6 months and 2 years after release (Figure 16) demonstrating that at least some of this group migrated to the GAB. What is remarkable though is that there are no longline recoveries from this group made in Central LL or Western LL with times‐at‐liberty of <5 years (Figure 16). Two of the recoveries made after more than 5 years at liberty are from the spawning grounds, and the others might have been on their way when recaptured (Figure 15). On the other hand, good numbers of recoveries from this group resulted from longline recaptures in Eastern LL with times‐at‐liberty ranging from around 1 year to more than 5 years. Presumably, some of the juveniles that migrated to the GAB survived and some of those that survived were later recaptured by longline vessels and their tags recovered. We conclude, therefore, that the juveniles tagged in Eastern LL that did migrate to the GAB and survived mostly returned to Eastern LL in subsequent winters.

Tags released from Western LL were much less frequently recovered from the surface fishery (Table 2, Figure 16) than tags released from the other longline fishing grounds. This suggests most juveniles in the western Indian Ocean at the time these tags were released did not migrate to the GAB. However, a few surface fishery recoveries among this tagging group indicate that a small proportion did. Again, presumably some juveniles that migrated from Western LL to the GAB survived but, in contrast to tags released from Eastern LL, no tags released from Western LL were recovered by longline fishers in Eastern LL.

Together Figures 15 and 16 indicate that migration choices of individuals summering in the GAB are dependent upon where the individual wintered previously. The distribution of longline recovery locations from conventional tag releases in Western LL and Eastern LL for times‐at‐liberty up to 5 years is not only different, but they exhibit no overlap. Given the number of conventional tags released in the Western LL and Eastern LL regions (Table 2) and the number of recoveries observed (Table 2 and Figure 16), the differences in tag recovery locations observed from these two groups of releases (Table 2, Figures 15 and 16) cannot be attributed to sampling variability. Qualitatively, it is difficult to imagine more convincing evidence of incomplete mixing in a genetically homogeneous population than is apparent in these data.

### 3.6 Electronic tagging studies

An acoustic tagging study, which tagged mostly 1‐year‐old SBT off WA, is described by Hobday, Kawabe, Takao, Miyashita, and Itoh (2009). Acoustic tags were surgically implanted in 1‐year‐old SBT off the WA coast over five consecutive summers from 2002–2003 to 2006–2007. Up to 70 listening stations, which included acoustic receivers, were moored in lines across the continental shelf and clustered at three inshore topographical features referred to as “lumps” off the south coast of WA. Acoustic tags emit a coded pulse so that when an acoustically tagged fish passes near a listening station, the identity, date, and time are recorded on the receiver (Hobday et al., 2009).

The acoustic tagging studies have provided information on interannual variability in departure times of individuals present along the southern coast of WA. They have also enabled studies of the effect of the Leeuwin Current and sea temperature on juvenile behavior. These studies had limited scope to inform migration rates because the locations of listening stations were restricted to the southern coast of WA. However, an adjustment made to the study in the fifth year of the study provided another opportunity to investigate incomplete mixing.

In 2006–2007, a total of 130 acoustic tags were released. These consisted of 50 tags released in the region off the south coast of WA, east of 118°E, where tagging had been carried out during the first 4 years of the study, but also 34 tags west of 118.2°E on the south coast and 46 tags released on the southern part of the west coast of WA. The proportions of released tags later detected by the array of acoustic receivers along the south coast varied greatly among the three release locations. Detections of tags released along the south coast are discussed in Fujioka et al. (2010, their table 1). Of the 50 tags released east of 118.2°E, 48 tags, or 96%, were later detected by acoustic receivers. Of the 34 tags released west of 118.2°E, only 13 tags, or 38%, were later detected. The 46 acoustic tags released on the west coast are not discussed by Fujioka et al. (2010), but according to Hobday et al. (2009), only one of the 46 tags, or 2%, was later detected within the array of acoustic receivers along the south coast of WA.

Hobday et al. (2009) observed that, as the individuals tagged on the west coast were similar in size to 1‐year‐olds released on the south coast, the difference in the proportion of tags detected could not be explained by mortality and must be instead due to migration. They concluded “not all the juvenile SBT population is in southern Australia during the austral summer” (Hobday et al., 2009, p. 419) and, considering interannual variation in the Leeuwin Current, suggested it was “reasonable to presume that the proportion of SBT moving in southern Western Australia may also vary between years” (pp. 419–420).

While information derived from acoustic tagging studies on SBT has provided some useful information, electronic archival tags, also referred to as “data storage tags” (see, e.g., Galuardi & Lam, 2014), have influenced theories about juvenile movement of SBT to a much greater extent. Archival tags, like acoustic tags, are surgically implanted inside individual fish when they are first captured. After the tags are inserted, the fish are released. However, whereas the acoustic tags emit a signal that enables the location of nearby fish to be detected on external receivers, in the case of archival tags, light‐based geolocation is used to derive the daily position of the fish (with error) from information that is stored on the electronic tag, inside the fish (see Basson et al., 2012; Gunn & Block, 2001). This means that the rich information stored on archival tags is only able to be observed if the tagged fish is later recaptured and the electronic device returned to scientists.

Electronic archival tags have been used to obtain daily locations of individual SBT since 1993 (Gunn & Block, 2001). Information sourced from archival tags revealed that neither the traditional nor alternative models adequately explained juvenile migration of SBT. Seasonal migration between SA and NSW had been evident from conventional tagging studies, but archival tagging studies revealed that a proportion of the juveniles resident in the GAB over summer migrated west to the SEIO during winter before returning to the GAB for the following summer.

An important archival tagging study of SBT was described in Basson et al. (2012). The study, which was run between 2003 and 2009, aimed to release archival tags implanted in juveniles smaller than 125 cm fork length across the full range of juvenile SBT habitat. A total of 570 archival tags were released southeast of South Africa (27 tags), in the Central Indian Ocean (159), off the south coasts of WA (177) and SA (122), and in the Tasman Sea off New Zealand (85) (Basson et al., 2012). Of these, 74 tags were later recovered for analysis, but two had retained no data and so could not be used. Basson et al. (2012) also had access to recoveries from earlier archival tagging studies so that overall 122 recoveries were available for their analyses. Based mostly on daily locations inferred from these 122 individual SBT, Basson et al. (2012, p. 2) state “the majority of juvenile SBT are likely to return to the GAB each summer and it is unlikely that a large proportion of juvenile SBT remain off South Africa over summer.” Although the daily location observations were not available to this study, we examine details of the archival tagging data stored on the CCSBT database and summary information included in Basson et al. (2012), Takahashi et al. (2004), and Itoh, Takahasi, Kurota, and Oshitani (2006) for additional information on juvenile movement and distribution.

The locations of recoveries of the archival tags were summarized by Basson et al. (2012). For convenience, we provide summaries of the full set of archival tag releases and recaptures stored on the CCSBT database in Table 3. These include the tags analyzed by Basson et al. (2012) and the conclusions are similar irrespective of which set of data is considered. We provide summaries of archival tag releases of individuals deemed to be 4 years old and below separately from releases of older individuals. Tags recovered after 90 or fewer days‐at‐liberty are excluded from these summaries. Of 1,545 archival tag releases of SBT 4 years and below, 188 have been recovered with times‐at‐liberty >90 days, and 150 of these recoveries have occurred via recapture by the surface fishery operating in the GAB (Table 3). The recovery of a considerable proportion of tags from the Australian fleet is to be expected because it presently catches a greater number of SBT than any of the other fleets and its SBT catch consists almost entirely of juveniles. The reporting rate of the Australian commercial fleet has also previously been assumed to be higher than the longline fleets (Polacheck et al., 2006). More problematic is the observation that the proportion of released tags that were recovered differed markedly depending on the location of release (Table 3). A much higher proportion of archival tags released from the GAB has been recovered (21%) compared with those released from the other locations (6%). Basson et al. (2012) summarize archival tag recoveries from tags released in different areas in the same years. The proportion of tags recovered from releases in the GAB was regularly substantially higher than from releases made from the other regions in the same year (Basson et al. 2012, their Table 6.2). This is evidence that the population tagged in the GAB behaved quite differently to the populations tagged elsewhere. We argue that behavioral differences affected the probability of recapture and tag recovery. Behaviors that increased the probability of recovery will tend to have been over‐represented in the set of recovered archival tags, whereas behaviors that result in decreased probability of recovery will have been under‐represented. Very few archival tags released with SBT 5 years of age and above have been recovered (Table 3). The fact that all six archival tag recoveries of older individuals tagged in Western LL occurred in the same region might indicate that the tendency for individuals to remain in this region, evidenced in Figures 15 and 16, continues into adulthood. These six Western LL recoveries occurred after times‐at‐liberty ranging from 187 to 1,274 days with a mean of 698 days.

**Table 3:** Summary of recoveries of archival tags by release location released between 1993 and 2009 with times‐at‐liberty between 90 days and 5 years

	Releases	Recoveries	Surface	Recovered by longline		
				West. LL	Cent. LL	East. LL
WA^a^						
≤4 years old	449	29 {0.06}	25 (0.86)	1 (0.03)	2 (0.07)	1 (0.03)
>4 years old	0	**–**	**–**	**–**	**–**	**–**
SA^b^						
≤4 years old	629	132 {0.21}	102 (0.77)	6 (0.05)	11 (0.08)	13 (0.10)
>4 years old	2	0	0	0	0	0
Western LL^b^						
≤4 years old	65	1 {0.02}	1 (1.00)	0 (0.00)	0 (0.00)	0 (0.00)
> 4 years old	126	6 {0.05}	0 (0.00)	6 (1.00)	0 (0.00)	0 (0.00)
Central LL						
≤4 years old	296	21 {0.07}	18 (0.86)	1 (0.05)	2 (0.10)	0
>4 years old	96	0	0	0	0	0
Eastern LL^a^						
≤4 years old	79	4 {0.05}	4 (1.00)	0 (0.00)	0 (0.00)	0 (0.00)
>4 years old	27	1 {0.04}	1 (1.00)	0 (0.00)	0 (0.00)	0 (0.00)

Numbers in braces are proportions of releases recovered from combined fishing grounds. Numbers in parentheses are proportions of total recoveries by release location. Note that data pertaining to releases of southern bluefin tuna (SBT) ≤4 years old from Western LL, Central LL, and Eastern LL are included in Table 2 and in Figures 15 and 16. (Source: Commission for the Conservation of Southern Bluefin Tuna.)

a An additional four tags were recovered by “beach walking,” three released from Western Australia (WA) and one from Eastern LL. In each case, the tags were recovered near the release location, but apparent times‐at‐liberty ranged from 95 to 1,695 days.

b An additional two tags were recovered, one released from Western LL and the other from South Australia (SA), but were missing recapture location details.

Basson et al. (2012) identified individuals among archival tag recoveries released from the GAB that went west of the 55°E meridian between May and November in the years 1993 to 2006. From a total of 93 observations (individual fish could be considered in multiple years), only six were observed west of 55°E between May and November. Furthermore, none of these go significantly west of 40°E. Two of the observations are from the same individual, a juvenile that summered in the Indian Ocean between the two winters, and few if any of the tracks appear to return to the GAB after crossing the 55°E meridian (Basson et al., 2012, their figure 8.4). That is, there is little or no evidence of juvenile SBT resident in the GAB in two consecutive summers that migrated west of the 55°E meridian in between these summers.

Comparing juvenile CPUE observed off South Africa with the SEIO (Figure 6) suggests the juvenile abundance of SBT south of Africa over winter might be similar or perhaps greater than in the SEIO. By contrast, the observed archival tags recovered from juveniles summering in the GAB exhibit almost no presence west of 60°E and none west of 40°E (Basson et al., 2012; their table 7.2). In short, the archival tagging data described by Basson et al. (2012) do not explain the presence of juvenile SBT off southern Africa evident in longline catch data (Figures 5 and 6). Instead, the observed recoveries demonstrate that the juveniles residing in the GAB over summer exhibit minimal intermixing with the juveniles regularly caught south of Africa.

Archival tags have also been released by Japanese scientific studies. These have included archival tags released off southern Africa, some of which have been recovered. Details of these releases are included in Table 3, but are not considered by Basson et al. (2012). Takahashi et al. (2004) describe daily locations inferred from three recovered archival tags released from the Western LL region. The recovered tags revealed that all three individuals remained at latitudes close to the 40°S parallel. One of the three migrated to the SEIO before returning to waters south of Africa, the other two remained west of 50°E. Takahashi et al. (2004) acknowledge that the number of archival tags recovered from the pilot program was quite low. A fourth archival tag released in western Indian Ocean discussed by Itoh et al. (2006) remained at large for more than 2 years, strictly west of 60°E, before being recaptured by a Taiwanese longline vessel. The qualitatively very different locations observed from the large number of recovered archival tags released in the GAB, described by Basson et al. (2012) compared to those released in the western Indian Ocean, described by Takahashi et al. (2004) and Itoh et al. (2006), considered together with substantial differences in the proportions of released archival tags that were recovered (Table 3), are suggestive of limited intermixing among individuals from these regions. The more recent archival tag recoveries, described in Basson et al. (2012), are consistent with the conclusions of Takahashi et al. (2004) who claimed earlier that accumulating evidence from conventional and archival tag returns “suggested a possibility for fish to tend to stay either eastern or western sides of (the) Indian Ocean” (p. 9) and after summarizing the tag recovery data added that the data “strongly indicate the need to reconsider (the) reliability of (the) complete mixing hypothesis.” The authors of this report have advised of their support for their work to be referenced and stated that their opinions have not changed on this matter (N. Takahashi, personal communication 2015).

## 4 PROPOSED JUVENILE DISTRIBUTION AND MOVEMENT

Begg and Waldman (1999) recommend a holistic approach to inferring fisheries stock structure, partly, they argue, because simultaneous consideration of multiple sources of evidence “maximizes the likelihood of correctly identifying stocks” (p. 39). Although we prefer to avoid referring to subgroups of juvenile SBT as “separate stocks”, we find considering evidence from different data types together indicates aspects of spatial structuring that would be difficult to infer from consideration of any single data source in isolation.

**Figure 17:**
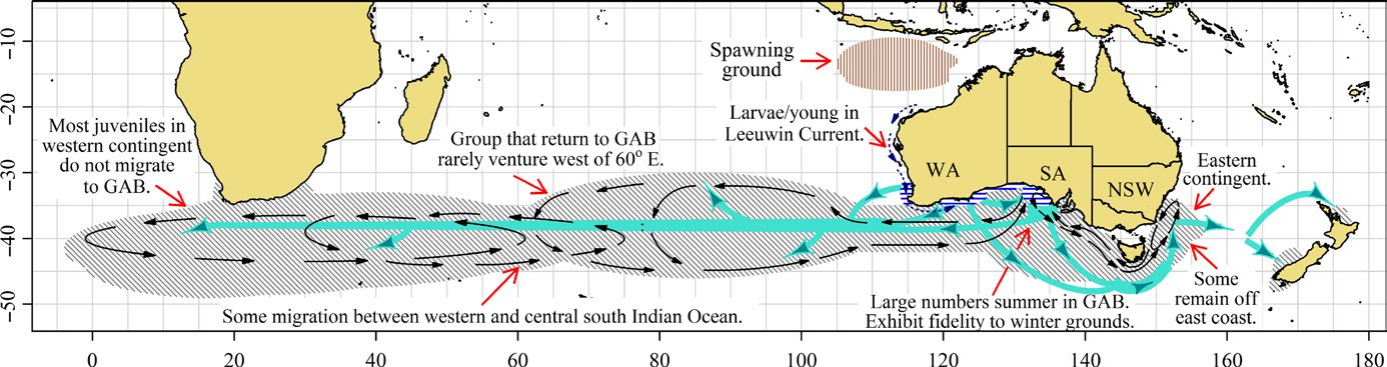
Diagram showing hypothesized and simplified migration and movement patterns of juvenile southern bluefin tuna up to the age of 5 years. Gray hatching indicates the approximate common distribution of 2‐ to 5‐year‐olds. The main known nursery grounds are shown as horizontal blue shading along the south and southwest coast of Australia. Thick turquoise arrows indicate distinct movements such as recruitment to contingents and emigration. Thin black arrows indicate general movements of individuals within contingents

In this section, we propose some characteristics of juvenile SBT distribution and migration that we contend are suggested by observations and analyses summarized in previous sections. To aid explanation, we depict key characteristics in Figure 17. Many of characteristics of the distribution and movement we propose are either well accepted or have been described previously (e.g., Basson et al., 2012; Caton, 1991; Murphy, 1977; Shingu, 1978). Diagrams published previously depicting earlier models of SBT migration are included in Supporting information for comparison. However, this study establishes some important aspects of juvenile SBT migration with greater certainty than was previously the case and provides important new insights. Figure 17 should be considered to give some general features of distribution and migration of juvenile SBT. The movements of individuals and schools will invariably depart considerably from those suggested by the figure. While some changes in the distribution over time have been documented, most of the core features seem to have been more stable than might have been expected.

Larvae or very small juveniles make their way south from the spawning grounds along the west coast of Australia in the Leeuwin Current making their way to the southwest coast of WA around age one. The general movement of juveniles from the southwest coast of WA is from west to east along the southern coast of Australia, but progressively groups peel off and move west into the southern Indian Ocean. 1‐ and 2‐year‐olds disperse from WA and 1‐ to 5‐year‐olds and older from SA and NSW, probably much as described in the alternative model (Fig.S3). As shown most clearly by electronic archival tags (see, e.g., Basson et al., 2012; Gunn & Block, 2001), a large population of 2‐ to 4‐year‐olds summer in the GAB and migrate seasonally east to the Tasman Sea or west into the SEIO. Recoveries of tags from oceanic releases (Figure 16) reveal juveniles that summer in the GAB exhibit fidelity to winter feeding grounds. A substantial proportion of the juveniles that move west into the Indian Ocean continue west to oceanic waters south of Africa. Tag recovery data, particularly tags released in oceanic waters (Figures 15 and 16), suggest that juveniles that migrate to the southwestern Indian Ocean and southeastern Atlantic Ocean only rarely migrate to southern Australia. Daily location data from archival tags suggest that juveniles that summer in the GAB rarely venture west of 60°E in the south central Indian Ocean (see Basson et al., 2012).

The pattern of tag recoveries from oceanic releases (Figures 15 and 16) suggests not only do juveniles remain separate on either side of the Indian Ocean, but that individuals summering in the GAB exhibit fidelity to winter feeding grounds. This important aspect of juvenile SBT behavior has not been demonstrated previously. Basson et al. (2012) suggest archival tag recoveries provide evidence individuals commonly switch winter feeding grounds. This might indicate that fidelity becomes stronger around 3 or 4 years of age.

Conventional tag recoveries and a limited number of archival tag recoveries suggest individuals in the western contingent mostly remain at longitudes between the 0° and 60°E meridians, although movements into the CIO and SEIO may be fairly common. Some individuals do migrate from the western Indian Ocean to the GAB, but this is uncommon. As this contingent grows, it probably accounts for a good proportion of the fishery in CCSBT area 9 (Figure 3). Caton (1991) suggests this has been the most important of the Japanese longline fisheries since it established in the late 1960s.

Recoveries of tags released from eastern Australia during the 1960s (Table 1) and from longline vessels in the Tasman Sea during the 1990s both suggest the existence of another subgroup of juveniles off eastern Australia with a unique migration pattern. Members of this subgroup are more likely to migrate to the GAB than juveniles in the western Indian Ocean, but those that do migrate to the GAB appear to exhibit fidelity to the winter feeding ground in the Tasman Sea (Figures 15 and 16). There is some migration of 2‐ to 4‐year‐olds to New Zealand, but this movement appears to be more common in slightly older individuals. As the fish in the eastern contingent age, their distribution likely expands to largely account for the fisheries in CCSBT areas 4, 5, and 6 (Figure 3).

## 5 DISCUSSION AND RECOMMENDATIONS

According to Hobday et al. (2015), combined conventional and archival tagging data suggest the proportions of 1‐year‐olds that remain off southwest Australia, move west into the Indian Ocean from WA, and move east into the GAB are unknown, but it is assumed that the majority are in the GAB by the following summer (p. 194). At present, there is little basis for estimating the proportion of juveniles that summer in the GAB and this proportion probably varies considerably among years. However, as we have shown, there is evidence from multiple data sources of incomplete mixing of cohorts among juveniles tagged in different locations. Additionally, there is persistent longline catch of juveniles in the western Indian Ocean and southeast Atlantic. Considerable numbers of archival tags have been recovered providing daily location data of seasonal migrations of juveniles that summer in the GAB, yet examples of juveniles migrating from the GAB to South Africa and back remain conspicuous by their absence.

Basson et al. (2012, p. 13) claim the archival tag recovery data summarized in their report provide a large amount of “fishery‐independent” movement information. Data from implanted archival tags are not fishery independent (Galuardi & Lam, 2014). As pointed out by Sippel et al. (2015), the observation of interim locational data from implanted archival tags between tagging and recapture is dependent on the fish being captured. The interim movements of juveniles recovered from the GAB (see Basson et al., 2012) are completely different from individuals recovered from the western Indian Ocean described by Takahashi et al. (2004) and Itoh et al. (2006). We argue that juveniles with archival tags whose movements were more consistent with those described by Takahashi et al. (2004) and Itoh et al. (2006) were relatively less likely to be recovered and are therefore likely to be under‐represented among recovered archival tags. Low rates of migration among juveniles tagged in the western Indian Ocean and related low probability of recovery explains the relatively low proportions of these tags that have been recovered (see Tables 2 and 3).

Although most of the analyses described in this study are qualitative and quite basic, limited intermixing between subgroups of juvenile SBT is an important finding that has previously not been well established. For example, Farley et al. (2007, p. 159) write “The proportion of the juvenile population that migrate towards South Africa each year is not known nor whether individuals show fidelity to that region.” They later add “SBT probably form one well‐mixed population rather than several independent groups” (Farley et al., 2007, p. 160). Hobday et al. (2015, p. 199) point out that little has been known about the movements of subadults (ages 5‐10 years) in particular. Analyses presented here suggest not only is intermixing between juvenile groups limited, but also that the groups tend to remain separate into the subadult stage and perhaps longer (Figure 16).

The demonstration of limited mixing between separate juvenile subgroups has implications for the interpretation of existing SBT data as well as future research priorities for this species. If, as proposed here, the proportion of juveniles that are outside of the GAB over summer varies between years and in some years is not negligible, recruitment indices based on aerial surveys in the GAB will be difficult to interpret (Basson et al., 2012, p. 177). It is hard to say what proportion of juveniles might be typically outside of the GAB at the time of the aerial survey, but apparent changes in behavior over time suggested by Polacheck, Hobday, West, Bestley, and Gunn (2006) and in Figures 12, 13, and 14 would indicate the proportion is unlikely to be constant. The reliability of the aerial survey for SBT as an index of juvenile abundance has been questioned recently by Sullivan (2015) who pointed out that the survey gave no signal of the apparently strong 2005 year class. Recruitment is estimated to have increased starting in 2005 from historical lows at the turn of the millennium (Hillary et al., 2016; their figure 1). This improvement in recruitment would have been expected to be seen as an increase in the aerial survey index from 2007 continuing in 2008 and 2009. In contrast, the aerial survey was near historical low levels over this period (see Hillary et al., 2016; their figure 2). The supposed low recruitment during the mid‐2000s suggested by GAB‐based indices of juvenile abundance was questioned earlier (Shiao et al., 2008) based on the stability of Taiwanese CPUE in the Indian Ocean over this period. The apparent discrepancy between the aerial survey and recruitment during the second half of the 2000s is consistent with an increased proportion of 2‐ to 4‐year‐olds summering outside of the GAB over this period.

The global population of SBT is presently very much smaller than it was during the 1960s. Recruitment and therefore juvenile abundance are also estimated to be typically considerably lower than was the case in the 1960s (see Hillary et al., 2016; their figure 1). It is plausible that a contraction in the distribution of juvenile SBT has accompanied the reduction in abundance and perhaps this could have resulted in a population that is mostly resident in the GAB over summer. However, the operation and catch of a Taiwanese fleet targeting juvenile SBT southeast of Africa as well as recoveries from the 2000s tagging study provide evidence that some juveniles continue to summer in this part of the western Indian Ocean.

Fidelity to winter feeding grounds, evident in Figure 16, suggests that migration choices in juvenile SBT cannot be assumed to be random responses to environmental stimuli. Rather, choices of individual fish are somehow related to where the fish has previously been. This aspect of juvenile SBT movement has not been demonstrated previously. Examples of contingents in fish migration described previously have often been examples of natal homing. Imprinting has been suggested as a possible mechanism for natal homing whereby newly mature adults follow spatial cues presumably observed in their early life history to their place of spawning. However, imprinting does not explain why sets of individuals would initially choose different winter feeding grounds when they have not previously visited any of them. The “adopted migrant” hypothesis proposed by McQuinn (1997) seems to be a better explanation for recruitment of 1‐year‐olds to a particular winter feeding ground. According to the adopted migrant or “entrainment” (see Petigas et al., 2006) hypothesis, migration paths result from social learning where young fish adopt successful migration circuits by following slightly older individuals. In this way, juveniles become entrained in established life cycle circuits (Secor & Kerr, 2009). Corten (2002) has suggested this method of social learning explains “conservatism” observed in various migration routes, not just spawning migration routes, in Atlantic herring.

Previous speculation about mechanisms for long distance migration and navigation is discussed in Hobday et al. (2015). Dell and Hobday (2008) speculated that an adopted migrant mechanism operating off the WA coast might explain apparent changes in the proportion of juvenile SBT migrating east. The only reasoning that was provided for this hypothesis was possibly larger numbers of 2‐year‐olds off WA during the 2000s when lower proportions of 1‐year‐olds off WA appear to have migrated east to the GAB than occurred during the 1990s. It was also evident in Dell and Hobday (2008, their figure 1) that tagging locations off WA during the 2000s were generally nearer to shore than was the case in earlier studies.

The characterization of the juvenile population off Eastern Australia as a distinct subgroup might explain the failure of the NSW fishery in the 1980s while the SA fishery remained viable (see Caton et al., 1990, p. 34). The disappearance of SBT from NSW during the 1980s has been interpreted as evidence of a range contraction in the spatial distribution of SBT in response to reduced abundance (Farley et al., 2007, p. 152). Secor (1999) notes that range contractions are consistent with populations that comprise contingent subgroups. The catch of the surface fishery off NSW was concentrated around December each year (Shingu, 1978; his figure 19) as juveniles formed dense schools on their southward migration along the eastern coast of Australia. Recovery rates of tags released from the EA region were sometimes very high and the recoveries were overwhelmingly sourced from the NSW surface fishery that operated at the time (see, e.g., Hampton, 1991; his table 4). This suggests not only that the tagged population off NSW was part of a separate group, but also that juvenile members of the group were subject to high exploitation rates at times. In these circumstances, the “sudden and continued absence” of SBT from the NSW coast (Caton, 1991, p. 309) seems consistent with the adopted migrant hypothesis (McQuinn, 1997). If recruitment to an eastern Australian contingent of young juveniles from the GAB relied upon them learning from slightly older juveniles returning to the GAB from NSW, the apparent heavy exploitation of the contingent could have resulted in the route around southern Tasmania being lost. Planque, Loots, Petitgas, Lindstrom, and Vaz (2011, p. 6) argue stock collapse is often associated with the collapse of spatial memory and contingent diversity. The observation of Caton et al. (1990, p. 1) that there had “been no significant recruitment of small fish to the longline fishery off New Zealand since 1980, whereas small fish represented about half the longline catch in this area during the 1960s” is also consistent with a putative Tasman Sea contingent that accounts for a significant proportion of SBT observed off New Zealand that had reduced juvenile abundance during the 1970s and 1980s. Shingu (1970) noted that years with higher proportions of 100–110 cm SBT in the early Japanese longline catch off New Zealand coincided with the appearance of the same group in the Tasmanian fishery north of 40°S, and that, furthermore, when these higher proportions of smaller fish were observed, apparently corresponding groups were observed in the catch on the same fishing grounds for “at least 3 or 4 years after their first appearance” (p. 32). This might indicate that individuals off NZ and northeast of Tasmania are, or were, from the same cohesive group, or contingent.

We have proposed that the juvenile SBT population typically includes a subgroup that resides in the western Indian Ocean mostly separate from the juveniles in the GAB and also separate from another subgroup off eastern Australia in the Tasman Sea that migrates to the GAB in summer somewhat more frequently. These two putative subgroups fail to account for an obviously significant proportion of the juvenile population that migrates to the SEIO in winter from the GAB. It is unclear if the remainder of the juvenile population are best classified as a third discrete subgroup or if the reality is more complicated. Conventional and archival tags released from the SEIO (or Central LL) have been recovered by the surface fishery in the GAB in proportions similar to releases from the Tasman Sea (Eastern LL). However, of roughly 400 archival and conventional tags released in Central LL, only four were later recovered from longliners operating in Central LL (Figure 15, Table 2). We note as well that Basson et al. (2012) observed “switching” between winter feeding grounds in three of eight archival tags for which two winters were observed. This rate of switching is higher than might be expected given apparently stronger fidelity to Eastern LL by the Tasman Sea subgroup. Archival tag recoveries also suggest a proportion of juveniles overwinter off southern Australia without migrating to either the Tasman Sea or the SEIO (Basson et al., 2012, p. 3).

The adopted migrant mechanism requires that established life cycle circuits exhibit spatial overlap with labile individuals (Petigas et al., 2006). The Tasman Sea, or eastern, contingent could adopt labile juveniles from the GAB via observed seasonal migrations between SA and NSW. However, the putative western Indian Ocean contingent is less easily explained by this mechanism, because the evidence we have described suggests few juvenile members of this contingent migrate to Australia. Perhaps juveniles could be recruited to this contingent by following juveniles from southern Australia into the Indian Ocean and then following members of the western Indian Ocean contingent when these groups mix in the southern Indian Ocean (Figure 17). This suggestion is speculative and we doubt existing data would be sufficient to shed much light on this matter.

The observation that 1‐year‐olds tagged off WA are typically less likely to be recovered by the surface fishery than are 1‐year‐olds tagged off SA and that longline recoveries of the 1‐year‐olds tagged off WA are more likely to result from recaptures in the western Indian Ocean is best explained by the existence of divergent juvenile migration paths where one or more groups never enter the GAB as juveniles. Preece et al. (2015) acknowledge the gene‐tagging‐based estimator of absolute cohort abundance they propose will be negatively biased under these circumstances.

The potential to derive quantitative estimates of migration from the Brownie design tagging studies run during the 1990s and 2000s should be investigated. Hampton (1991) estimated migration rates between the Australian fisheries and the Japanese longline fishery from the early tagging data. The Brownie design of the more recent studies might provide additional opportunities for quantitative modeling. However, evidence of fidelity to winter feeding grounds implies models assuming Markovian migration choices would be questionable. Spatially structured models for SBT tag recovery data have been discussed previously (see, e.g., Polacheck et al., 2006; their Appendix 11; Eveson et al., 2012). However, the present study suggests the migration assumptions of these models need to be relaxed. Specifically, whereas both models treat “southern Australia” (WA and SA combined) as a single region, differences in recovery probabilities mean these locations need to be treated as separate regions. While, Eveson et al. (2012) assume Markovian movement, Polacheck et al. (2006, their Appendix 11) consider a variant allowing fidelity to winter feeding grounds. Unfortunately, unknown reporting rates of the longline fleets in particular would limit the parameters that could be estimated. Nevertheless, some useful parameters might be able to be estimated because of the Brownie design of the tagging study.

Otolith microchemistry has been vital in discriminating between individuals from different juvenile habitats for many species including Pacific bluefin tuna (*T. orientalis*) (Rooker, Secor, Zdanowicz, & Itoh, 2001) and Atlantic bluefin tuna (Rooker et al., 2008). The application of these techniques to SBT has been investigated (see Gunn, Harrowfield, Proctor, & Thresher, 1992; Proctor et al., 1995), but the identification of natural markers in SBT otoliths has proven elusive, presumably because the chemical compositions of their various habitats are not very different. Lin, Wang, You, and Tzeng (2013) have suggested high ratios of barium to calcium in otolith profiles might be associated with instances of residence in the GAB. The ability to estimate the proportion of different age classes that were resident in the GAB as juveniles from a random sample of the catch would represent a major advance. However, the approach described by Lin et al. (2013) needs to be validated in a broader study including otoliths from individuals harvested in the GAB.

Based on what we argue is the broadest analysis of data related to the distribution and movement of juvenile SBT to date, we conclude the evidence strongly favors the existence of multiple subgroups of juvenile SBT that remain mostly separate, as suggested by Takahashi et al. (2004) and implied earlier by other researchers (Caton, 1991; Hampton, 1989; Murphy, 1977). Conventional and archival tagging data appear to indicate that only a minority of juveniles present in the western Indian Ocean and eastern Atlantic Ocean in winter migrate to the GAB in summer. Unfortunately, the results from the Takahashi et al. (2004) and Itoh et al. (2006) have not been made widely available. We suggest a collaborative study of the full set of recovered archival tags contrasting movement behavior by release location would give an improved understanding of spatial substructure in juvenile SBT. Analyses of longline catch and effort data, although imprecise, would suggest that the proportion of the total juvenile population in this area is unlikely to be negligible. Differences in the characteristics of tag recovery data between tagging periods indicate that the proportion of juveniles outside the GAB over summer probably varies between years.

The development of a spatially explicit integrated stock assessment for SBT has been previously proposed (e.g., Hillary et al., 2016). The findings of the present study support this proposal and provide information on what would be appropriate assumptions of a spatial population dynamics model. The conclusion of incomplete mixing does not necessarily imply management of SBT needs to be spatially explicit. The collapse of the NSW fishery in the 1980s provides an extreme example of how the migratory behavior of juveniles can influence the response of the population to high localized harvest rates in certain circumstances. However, it seems unlikely that such high harvest rates would be allowed to occur now that the global TAC is set by the CCSBT. The implications of separate juvenile subgroups for the interpretation of existing datasets should be considered and proposed research projects should be assessed cognizant that the proportion of juveniles that summer outside of the GAB is probably not negligible and likely varies from year to year.

## CONFLICT OF INTEREST

None declared.

## AUTHOR CONTRIBUTIONS

MSC conceived and designed the study. MSC, LAS, and BO negotiated permission to use the data. MSC, LAS, BO, and NS contributed to analysis and interpretation. MSC drafted the article. MSC, LAS, BO, and NS contributed to revision of the article. MSC, LAS, BO, and NS approved final submission.

## DATA ACCESSIBILITY

Data analyzed in this document are managed by the Commission for the Conservation of Southern Bluefin Tuna https://www.ccsbt.org/ unless otherwise indicated. Access to these data is restricted. Contact: Colin Millar, cmillar@ccsbt.org.

## APPENDIX 1 DIFFERENCES IN PROPORTION OF RELEASES RECOVERED BY SURFACE FISHERY

### 1.1

Consider a population of juvenile SBT consisting of *T*_1_members of a single cohort that were tagged at a particular location at age one. Assume the population is subject to a general stochastic process of fishing and death as described in Quinn and Deriso (1999, chapter 1), but allow the probability of capture and survival of a randomly selected individual at any point after tagging to depend on whether or not the individual is in the Great Australian Bight (GAB) or, alternatively, elsewhere within the juvenile distribution of SBT. Let *f*_*g*_(*t*) be the probability that a randomly chosen individual from the population is captured at instant *t* given it is alive and in the GAB at time *t* and *f*_*e*_(*t*) be the probability that an individual is captured at instant *t* given it is alive and outside the GAB at time *t*. Let *m*_*g*_(*t*) and *m*_*e*_(*t*) be the probabilities that an individual from the population dies of causes other than fishing at instant *t* given it is alive and inside the GAB and alive and outside of the GAB, respectively. Let ψ_1_(*t*) be the probability that a surviving individual from the tagged population is in the GAB at time *t*. If *t*_0_ is the time when the individuals are tagged, then the probability an individual from the population remains alive at time *t* after tagging is expressed as:

$$r_{1}\left(t\right)=\exp\left\{-\int_{t_{0}}^{t}\left[\psi_{1}\left(x\right)\left\{f_{g}\left(x\right)+m_{g}\left(x\right)\right\}+\left(1-\psi_{1}\left(x\right)\right)\left\{f_{e}\left(x\right)+m_{e}\left(x\right)\right\}\right]dx\right\}.$$

A member of the tagged population can be caught by the Australian purse seine fleet during some time interval between *t*_*k*_ and *t*_*k*_ + Δ*t* provided the individual is alive at time *t*_*k*_ and is present in the GAB at some stage during the time interval. The probability is:

$$p_{1}=\int_{t_{k}}^{t_{k}+\Delta t}\psi_{1}\left(t\right)f_{g}\left(t\right)r_{1}\left(t\right)dt\;.$$

If ρ_*g*_(*t*) is the probability a tag recaptured by the surface fishery at instant *t* is reported, then the probability a tagged individual is recaptured at time t and its tag(s) reported is

$$p_{1}^{\prime }=\int_{t_{k}}^{t_{k}+\Delta t}\psi_{1}\left(t\right)\rho_{g}\left(t\right)f_{g}\left(t\right)r_{1}\left(t\right)dt\;.$$

The expected number of recoveries from surface fishery recapture between *t*_*k*_ and *t*_*k*_ + Δ*t* is $E\left(S_{1}\right)=T_{1}p_{1}^{\prime }$.

Consider now a second population of *T*_2_ juvenile SBT from the same cohort as the first also tagged at age one, but tagged at a different location. As the second population is the same age as the first, it is reasonable to assume that the probability of recapture and natural mortality of members of this population will also be *f*_*g*_(*t*) and *m*_*g*_(*t*) for individuals in the GAB at instant *t* and *f*_*e*_(*t*) and *m*_*e*_(*t*) for individuals outside the GAB. That is, fishing mortality and natural mortality might depend on age/time and location, but conditional on its location, we would not expect these probabilities to depend on where an individual was located when it was tagged some months or years earlier at time $t_{0}$. Also if a tagged individual was recaptured by a purse seine vessel operating in the GAB at time *t*, we would not expect the reporting probability, ρ_*g*_(*t*), to depend on tagging location. Therefore, letting the probability an individual from the second group is in the GAB at time *t* be ψ_2_(*t*), the expected proportion of the second population to be recovered during the interval between *t*_*k*_ and *t*_*k*_ + Δ*t* is given by:

$$E\left(\frac{S_{2}}{T_{2}}\right)=p_{2}^{\prime },\;\;\text{where}$$

$$p_{2}^{\prime }=\int_{t_{k}}^{t_{k}+\Delta t}\psi_{2}\left(t\right)\rho_{g}\left(t\right)f_{g}\left(t\right)r_{2}\left(t\right)dt,\;\text{and}$$

$$r_{2}\left(t\right)=\exp\left\{-\int_{t_{0}}^{t}\left[\psi_{2}\left(x\right)\left\{f_{g}\left(x\right)+m_{g}\left(x\right)\right\}+\left(1-\psi_{2}\left(x\right)\right)\left\{f_{e}\left(x\right)+m_{e}\left(x\right)\right\}\right]dx\right\}.$$

It follows that $p_{2}^{\prime }\neq p_{1}^{\prime }\Rightarrow \psi_{2}\neq \psi_{1}$.

Therefore, evidence of different recovery probabilities can be interpreted as evidence of differences in temporal probability of presence in the GAB and thus incomplete mixing of the two subpopulations. The relationship between $p_{2}^{\prime }-p_{1}^{\prime }$ and presence in the GAB depends on the unknown time‐dependent mortality and tag reporting rates defined above, but it would be expected that $p_{2}^{\prime }>p_{1}^{\prime }$ would occur when the second population were more often present in the GAB than the first population at times when *f*_*g*_(*t*) was high.

#### DIFFERENCES IN SPATIAL DISTRIBUTION OF LONGLINE RECOVERIES

The reasoning used for the surface fishery can be applied similarly for each of the three longline fishing areas, Western LL, Central LL, and Eastern LL. Again we consider individuals from a single cohort tagged at a particular location, but this time we consider individuals tagged up to and including age three. Let *N*_1_ = *r*_1,1_ + *r*_1,2_ + *N*_1,3_ be the number of tagged members of the cohort alive at age 3 with *r*_1,1_ the number surviving from the group tagged at age 1 and *r*_1,2_ the number surviving from the group tagged at age 2 and *N*_1,3_ the number from the cohort tagged at age 3. Then, the expected number of recoveries from this cohort recaptured by the longline fleet in Eastern LL between the ages of 3 and 6 is given by:

$$E\;\left(Z_{1}\right)=N_{1}p_{E1}^{\prime },$$

where

$$p_{E1}^{\prime }=\int_{3}^{6}\psi_{E1}\left(t\right)\rho_{E}\left(t\right)f_{E}\left(t\right)r_{1}\left(t\right)dt$$

and

$$r_{1}\left(t\right)=\exp\left\{-\;\int_{3}^{t}\left[\psi_{E1}\left(x\right)\;\left\{f_{E}\left(x\right)+m_{E}\left(x\right)\right\}+\left(1-\psi_{E1}\left(x\right)\right)\;\left\{f_{\neg E}\left(x\right)+m_{\neg E}^{*}\left(x\right)\right\}\right]\;dx\;\right\}\;.$$

ψ_*E*1_(*t*) is the probability any particular member of the group tagged at the first location is located in Eastern LL at instant *t*.

*f*_*E*_(*t*) is the probability of longline capture given located in Eastern LL at instant *t*.

*m*_*E*_(*t*) is the probability of other death given located in Eastern LL at instant *t*.

*f*_¬*E*_(*x*) is the probability of longline capture given located outside Eastern LL.

$m_{\neg E}^{*}\left(x\right)$ is the probability of other death including surface fishery recapture if located outside Eastern LL.

ρ_*E*_(*t*) is the probability a tag recovered by the Eastern LL fishery at instant *t* is reported.

We argue it is reasonable to assume that the conditional probabilities, *f*_*E*_(*t*), *m*_*E*_(*t*), *f*_¬*E*_(*x*), $m_{\neg E}^{*}\left(x\right)$ and ρ_*E*_(*t*) depend on the age of the individual at time *t*, but not the age of the individual when it was tagged. The probability ψ_*E*1_(*t*) may depend on the age the individual was tagged. Differences in ψ_*E*1_(*t*) between members of the cohort tagged in location 1 at different ages would be evidence of nonmixing of this subgroup. This could be investigated further but is not formally considered in the present study.

Using the same reasoning corresponding expressions for the expected number of tags from longline recapture from Central LL, *C*_1_, and Western LL, *W*_1_,  could be derived with:

$E\left(W_{1}\right)=N_{1}p_{W1}^{\prime }$ and $E\left(C_{1}\right)=N_{1}p_{C1}^{\prime }.$

The quantities $p_{W1}^{\prime }$ and $p_{C1}^{\prime }$ are defined analogously to $p_{E1}^{\prime }.$ The total number of longline recoveries expected from the cohort between the ages of three and six is

$$E\left(L_{1}\right)=N_{1}\left(p_{W1}^{\prime }+p_{C1}^{\prime }+p_{E1}^{\prime }\right)\;.$$

The expected proportion of longline recoveries occurring in Eastern LL, for example, is:

$$E\left(\frac{Z_{1}}{L_{1}}\right)=\frac{N_{1}p_{E1}^{\prime }}{N_{1}\left(p_{W1}^{\prime }+p_{C1}^{\prime }+p_{E1}^{\prime }\right)}=\frac{p_{E1}^{\prime }}{p_{W1}^{\prime }+p_{C1}^{\prime }+p_{E1}^{\prime }}.$$

Using similar reasoning as for the surface fishery recoveries, we expect members of the same cohort tagged at a different location will be subject to the same probabilities of recapture, other death and tag reporting conditional on their location. Therefore, for *N*_2_ = *r*_2,1_ + *r*_2,2_ + *N*_2,3_ tags released from tagging location 2 surviving at age 3, the expected number of recoveries of individuals recaptured by Eastern LL between the ages of 3 and 6 is:

$$E\left(Z_{2}\right)=N_{2}p_{E2}^{\prime },$$

where

$$p_{E2}^{\prime }=\int_{3}^{6}\psi_{E2}\left(t\right)\rho_{E}\left(t\right)f_{E}\left(t\right)r_{2}\left(t\right)dt$$

and

$$r_{2}\left(t\right)=\exp\left\{-\int_{3}^{t}\left[\psi_{E2}\left(x\right)\left\{f_{E}\left(x\right)+m_{E}\left(x\right)\right\}+\left(1-\psi_{E2}\left(x\right)\right)\left\{f_{\neg E}\left(x\right)+m_{\neg E}^{*}\left(x\right)\right\}\right]dx\right\}.$$

It follows that $p_{E2}^{\prime }\neq p_{E1}^{\prime }\Rightarrow \psi_{E2}\left(t\right)\neq \psi_{E1}\left(t\right)$.

The expected proportions of longline recoveries among releases from location 2 made in Eastern LL is

$$E\left(\frac{Z_{2}}{L_{2}}\right)=\frac{N_{2}p_{E2}^{\prime }}{N_{2}\left(p_{W2}^{\prime }+p_{C2}^{\prime }+p_{E2}^{\prime }\right)}=\frac{p_{E2}^{\prime }}{p_{W2}^{\prime }+p_{C2}^{\prime }+p_{E2}^{\prime }}.$$

Overall, we conclude that differences in reporting rates and mortality rates between recapture regions would not be expected to result in differences in the spatial distribution of tags among subgroups from the same cohort if the cohort were well mixed. Rather differences of this kind are likely to result from differences in probability of presence of the two subgroups in each region as a function of time $\psi_{E2}\left(t\right)\neq \psi_{E1}\left(t\right)$, $\psi_{C2}\left(t\right)\neq \psi_{C1}\left(t\right)$ and $\psi_{W2}\left(t\right)\neq \psi_{W1}\left(t\right)$.

## References

[ece33500-bib-0001] Basson, M. , Hobday, A. J. , Eveson, J. P. , & Patterson, T. A. (2012) Spatial interactions among juvenile southern bluefin tuna at the global scale: A large scale archival tag experiment. *Final Report for Fisheries Research and Development Corporation Project No 2003/002*, 347 pp. http://frdc.com.au/research/final-reports/Pages/2003-002-DLD.aspx. Accessed 20 July 2015.

[ece33500-bib-0002] Begg, G. A. , & Waldman, J. R. (1999). An holistic approach to fish stock identification. Fisheries Research, 43, 35–44.

[ece33500-bib-0003] Brownie, C. , Anderson, D.R. , Burnham, K.P. , & Robson, D.S. (1985) Statistical inference from band recovery data: A handbook. 2nd edn(Fish and Wildlife Service Resource Publication, No. 156). United States Department of the Interior, Washington D.C.

[ece33500-bib-0004] Butterworth, D. S. , Ianelli, J. N. , & Hilborn, R. (2003). A statistical model for stock assessment of southern bluefin tuna with temporal changes in selectivity. African Journal of Marine Science, 25, 331–361.

[ece33500-bib-0005] Cadrin, S. X. , & Secor, D. H. (2009). Accounting for spatial population structure in stock assessment: Past, present and future In Beamish R. J., & Rothschild B. J. (Eds.), The future of fisheries science in North America (pp. 405–426). Berlin, Germany: Springer Science.

[ece33500-bib-0006] Campbell, D. , Brown, D. , & Battaglene, T. (2000). Individual transferable catch quotas: Australian experience in the southern bluefin tuna fishery. Marine Policy, 24, 109–117.

[ece33500-bib-0007] Caton, A. (1991). Review of aspects of southern bluefin tuna biology, population and fisheries In Deriso R. B., & Bayliff W. H. (Eds.), World meeting on stock assessment of Bluefin Tunas: Strengths and weaknesses (pp. 181–350). Inter‐American Tropical Tuna Commission: La Jolla.

[ece33500-bib-0008] Caton, A. , McLaughlin, K. , & Williams, M. J. (1990). Southern Bluefin Tuna: Scientific background to the debate. Canberra, ACT, Australia: Commonwealth Government Printer.

[ece33500-bib-0009] Chambers, M. S. , Sidhu, L. A. , & O'Neill, B. (2014). Southern bluefin tuna (*Thunnus maccoyii*) shed tags at a higher rate in tuna farms than in the open ocean – two‐stage tag retention models. Canadian Journal of Fisheries and Aquatic Sciences, 71, 1220–1228.

[ece33500-bib-0010] Corten, A. (2002). The role of “conservatism” in herring migrations. Reviews in Fish Biology and Fisheries, 11, 339–361.

[ece33500-bib-0011] Dell, J. T. , & Hobday, A. J. (2008). School‐based indicators of tuna population status. ICES Journal of Marine Science, 65, 612–622.

[ece33500-bib-0012] Ellis, D. , & Kiessling, I. (2016). Ranching of southern bluefin tuna in Australia In Benetti D. D., Partridge G. J., & Buentello A. (Eds.), Advances in Tuna Aquaculture: From hatchery to market (pp. 217–232). Tokyo: Academic Press.

[ece33500-bib-0013] Eveson, J. P. , Basson, M. , & Hobday, A. J. (2012). Using electronic tag data to improve mortality and movement estimates in a tag‐based spatial fisheries assessment model. Canadian Journal of Fisheries and Aquatic Sciences, 69, 869–883.

[ece33500-bib-0015] Farley, J. H. , Davis, T. L. O. , Gunn, J. S. , Clear, N. P. , & Preece, A. L. (2007). Demographic patterns of southern bluefin tuna, *Thunnus maccoyii*, as inferred from direct age data. Fisheries Research, 83, 151–161.

[ece33500-bib-0016] Fujioka, K. , Hobday, A. J. , Kawabe, R. , Miyashita, K. , Honda, K. , Itoh, T. , & Takao, Y. (2010). Interannual variation in summer habitat utilization by juvenile southern bluefin tuna (*Thunnus maccoyii*) in southern Western Australia. Fisheries Oceanography, 19, 183–194.

[ece33500-bib-0017] Galuardi, B. , & Lam, C. H. (2014). Telemetry analysis of highly migratory species In Cadrin S. X., Kerr L. A., & Mariani S. (Eds.), Stock identification methods, 2nd ed. (pp. 447–476). San Diego: Academic Press.

[ece33500-bib-0018] Gelman, A. , & Rubin, D. B. (1992). Inference from iterative simulation using multiple sequences. Statistical Science, 7, 457–511.

[ece33500-bib-0019] Grewe, P. M. , Elliott, N. G. , Innes, B. H. , & Ward, R. D. (1997). Genetic population structure of southern bluefin tuna (*Thunnus maccoyii*). Marine Biology, 127, 555–561.

[ece33500-bib-0020] Gunn, J. , & Block, B. A. (2001). Advances in acoustic, archival and pop‐up satellite tagging of tunas In Block B. A., & Stevens E. D. (Eds.), Tunas: Physiology, ecology and Evolution (pp. 167–224). San Diego: Academic Press.

[ece33500-bib-0021] Gunn, J. , Farley, J. , & Hearn, B. (2003) Catch‐at‐age; age at first spawning; historical changes in growth; and natural mortality of SBT: An integrated study of key uncertainties in the population biology and dynamics of SBT based on direct age estimates from otoliths. *Final Report for Fisheries Research and Development Corporation, Project No 1997/111*, 91 pp. http://frdc.com.au/research/final-reports/Pages/1997-111-DLD.aspx. Accessed 23 September 2015.

[ece33500-bib-0022] Gunn, J. S. , Harrowfield, I. R. , Proctor, C. H. , & Thresher, R. E. (1992). Electron probe microanalysis of fish otoliths – evaluation of techniques for studying age and stock discrimination. Journal of Experimental Marine Biology and Ecology, 158, 1–36.

[ece33500-bib-0023] Hampton, J. (1989). Population dynamics, stock assessment and fishery management of the southern bluefin tuna (Thunnus maccoyii), PhD thesis, (p. 273). Sydney, NSW, Australia: University of New South Wales.

[ece33500-bib-0024] Hampton, J. (1991). Estimation of southern bluefin tuna *Thunnus maccoyii* natural mortality and movement rates from tagging experiments. Fishery Bulletin, 89, 591–610.

[ece33500-bib-0025] Hilborn, R. (1990). Determination of fish movement patterns from tag recoveries using maximum likelihood estimators. Canadian Journal of Fisheries and Aquatic Sciences, 47, 635–643.

[ece33500-bib-0026] Hillary, R. M. , Preece, A. L. , Davies, C. R. , Kurota, H. , Sakai, O. , Itoh, T. , … Branch, T. A. (2016). A scientific alternative to moratoria for rebuilding depleted international tuna stocks. Fish and Fisheries, 17, 469–482.

[ece33500-bib-0027] Hobday, A. J. , Evans, K. , Eveson, J. P. , Farley, J. H. , Hartog, J. R. , Basson, M. , & Patterson, T. A. (2015). Distribution and migration – southern bluefin tuna (*Thunnus maccoyii*) In Kitagawa T., & Kimura S. (Eds.), Biology and ecology of Bluefin Tuna (pp. 189–210). Boca Raton, FL: CRC Press.

[ece33500-bib-0028] Hobday, A. J. , Kawabe, R. , Takao, Y. , Miyashita, K. , & Itoh, T. (2009). Correction factors derived from acoustic tag data for a juvenile southern bluefin tuna abundance index in Southern Western Australia In Nielsen J. (Ed.), Electronic tagging and tracking in marine fisheries II. Reviews: Methods and technologies in fish biology and fisheries. Berlin, Heidelberg: Springer Netherlands.

[ece33500-bib-0029] Hunter, J. R. , Argue, A. W. , Bayliff, W. H. , Dixon, A. E. , Fonteneau, A. , Goodman, D. , & Seckel, G. R. (1986). The dynamics of Tuna movements: An evaluation of past and future research. FAO Fisheries Technical Paper 277. Rome, Italy: FAO.

[ece33500-bib-0031] Hynd, J. S. , & Lucas, C. (1975). Population dynamics of the southern bluefin tuna. Indo‐Pacific Fisheries Council Proceedings, 15, 424–435.

[ece33500-bib-0032] Ishizuka, Y. (1987) Migration and growth of southern bluefin tuna based on Australian tagging data In: Sixth Southern Bluefin Tuna Trilateral Scientific Meeting, SBFWS/87/10, 17‐21 August 1993, Hobart, Australia.

[ece33500-bib-0033] Itoh, T. , Takahasi, N. , Kurota, H. , & Oshitani, S. (2006) Report of activities for conventional and archival tagging of southern bluefin tuna by Japan in 2005/2006 and proposal of tagging in 2006/2007. CCSBT‐ESC/0609/36.

[ece33500-bib-0035] Kolody, D. S. , Eveson, J. P. , & Hillary, R. M. (2016). Modelling growth in tuna RFMO stock assessments: Current approaches and challenges. Fisheries Research, 180, 177–193.

[ece33500-bib-0036] Kolody, D. , & Hoyle, S. (2015). Evaluation of tag mixing assumptions in western Pacific Ocean skipjack tuna stock assessment models. Fisheries Research, 163, 127–140.

[ece33500-bib-0037] Kurota, H. , Hiramatsu, K. , & Tsuji, S. (2002). Simulation model toward development of assessment procedures of tagging data. CCSBT‐SC/0209/18.

[ece33500-bib-0039] Latour, R. J. , Hoenig, J. M. , Olney, J. E. , & Pollock, K. H. (2001). A simple test for nonmixing in multiyear tagging studies: Application to striped bass tagged in the Rappahannock River, Virginia. Transactions of the American Fisheries Society, 130, 848–856.

[ece33500-bib-0040] Lin, Y. T. , Wang, C. H. , You, C. F. , & Tzeng, W. N. (2013). Ba/Ca ratios in otoliths of southern bluefin tuna (*Thunnus maccoyii*) as a biological tracer of upwelling in the Great Australian Bight. Journal of Marine Science and Technology (Taiwan), 21, 733–741.

[ece33500-bib-0041] McGarvey, R. , & Feenstra, J. E. (2002). Estimating rates of fish movement from tag recoveries: Conditioning by recapture. Canadian Journal of Fisheries and Aquatic Sciences, 59, 1054–1064.

[ece33500-bib-0042] McQuinn, I. H. (1997). Metapopulations and the Atlantic herring. Reviews in Fish Biology and Fisheries, 7, 297–329.

[ece33500-bib-0043] Murphy, G. I. (1977). New understanding of southern bluefin tuna. Australian Fisheries, 36, 2–6.

[ece33500-bib-0044] Murphy, G. I. , & Majkowski, J. (1981). State of the southern bluefin tuna population: Fully exploited. Australian Fisheries, 40, 20–29.

[ece33500-bib-0045] Nakamura, H. (1969). Tuna distribution and migration. London: Fishing News.

[ece33500-bib-0046] Olson, R. J. (1980). Synopsis of the biological data on the southern bluefin tuna, *Thunnus maccoyii* (Castelnau, 1872) In Bayliff W. H. (Ed.), Synopses of biological data on eight species of scombrids (pp. 151–212). London: Inter‐American Tropical Tuna Commission Special Report No. 2.

[ece33500-bib-0047] Petigas, P. , Reid, D. , Planque, B. , Norgueira, E. , O'Hea, B. , & Contano, U. (2006). The entrainment hypothesis: An explanation for the persistence and innovation in spawning migration and life cycle patterns. ICES CM, B: 07.

[ece33500-bib-0048] Planque, B. , Loots, C. , Petitgas, P. , Lindstrom, U. , & Vaz, S. (2011). Understanding what controls the spatial distribution of fish populations using a multi‐model approach. Fisheries Oceanography, 20, 1–17.

[ece33500-bib-0049] Polacheck, T. , Hobday, A. , West, G. , Bestley, S. , & Gunn, J. (2006) Comparison of east‐west movements of archival tagged southern bluefin tuna in the 1990s and early 2000s. CCSBT‐ESC/0609/28.

[ece33500-bib-0050] Polacheck, T. , Laslett, G. M. , & Eveson, J. P. (2006) Estimation of mortality rates from tagging data for pelagic fisheries: Analysis and experimental design. *Final Report for Fisheries Research and Development Corporation Project No 2002/015*, 47 pp http://frdc.com.au/research/final-reports/Pages/2002-015-DLD.aspx (accessed 13 September 2015).

[ece33500-bib-0051] Pollock, K. H. , & Ravelling, D. G. (1982). Assumptions of modern band‐recovery models, with emphasis on heterogeneous survival rates. The Journal of Wildlife Management, 46, 88–98.

[ece33500-bib-0052] Preece, A. , Davies, C. , Bravington, M. , Hillary, R. , Eveson, P. , & Grewe, P. (2013) Preliminary cost and precision estimates of sampling designs for gene‐tagging of SBT, CCSBT‐ESC/1309/18.

[ece33500-bib-0053] Preece, A. , Eveson, P. , Davies, C. , Grewe, P. , Hillary, R. , & Bravington, M. (2015) Report on gene‐tagging design study. CCSBT‐ESC/1509/18.

[ece33500-bib-0054] Proctor, C. H. , Thresher, R. E. , Gunn, J. S. , Mills, D. J. , Harrowfield, I. R. , & Sie, S. H. (1995). Stock structure of the southern bluefin tuna *Thunnus maccoyii*: An investigation based on probe microanalysis of otolith composition. Marine Biology, 122, 511–526.

[ece33500-bib-0055] Quinn, T. J. II , & Deriso, R. B. (1999). Quantitative fish dynamics. Oxford, NY, USA: Oxford University Press.

[ece33500-bib-0057] Robins, J. P. (1963). Synopsis of biological data on southern bluefin tuna *Thunnus thynnus maccoyii* (Castelnau) 1872. FAO Fisheries Report, 6, 562–587.

[ece33500-bib-0058] Rooker, J. R. , Secor, D. H. , De Metrio, G. , Schloesser, R. , Block, B. A. , & Neilson, J. D. (2008). Natal homing and connectivity in Atlantic bluefin tuna populations. Science, 322, 742–744. 18832611 10.1126/science.1161473

[ece33500-bib-0059] Rooker, J. R. , Secor, D. H. , Zdanowicz, V. S. , & Itoh, T. (2001). Discrimination of northern bluefin tuna from nursery areas in the Pacific Ocean using otolith chemistry. Marine Ecology Progress Series, 218, 275–282.

[ece33500-bib-0060] Seber, G. A. F. (1973). The estimation of animal abundance and related parameters. London: Griffin.

[ece33500-bib-0061] Secor, D. H. (1999). Specifying divergent migrations in the concept of stock: The contingent hypothesis. Fisheries Research, 43, 13–34.

[ece33500-bib-0062] Secor, D. H. (2015). Migration ecology of marine fishes. Baltimore: John Hopkins University Press.

[ece33500-bib-0063] Secor, D. H. , & Kerr, L. A. (2009). Lexicon of life cycle diversity in diadromous and other fishes. American Fisheries Society Symposium, 69, 537–556.

[ece33500-bib-0065] Shiao, J. C. , Chang, S. K. , Lin, Y. T. , & Tzeng, W. N. (2008). Size and age composition of southern bluefin tuna (*Thunnus maccoyii*) in the central Indian Ocean inferred from fisheries and otolith data. Zoological Studies, 47, 158–171.

[ece33500-bib-0066] Shingu, C. (1967). Distribution and migration of the southern bluefin tuna. Report of the Nankai Regional Fisheries Research Laboratory, 25, 19–34.

[ece33500-bib-0067] Shingu, C. (1970). Studies relevant to distribution and migration of the southern bluefin tuna. Bulletin of Far Seas Fisheries Research Laboratory, 3, 57–113. [In Japanese. English translation by M.A. Hintz.]

[ece33500-bib-0068] Shingu, C. (1978) Ecology and stock of southern bluefin tuna. Japan Association of Fishery Resource Protection Fisheries Study No. 31, 79 pp. [In Japanese. English translation in the form of Hintze, M.A. (1981) CSIRO Division of Fisheries and Oceanography Report No. 131, 81 pp.]

[ece33500-bib-0069] Sippel, T. , Eveson, J. P. , Galuardi, B. , Lam, C. , Hoyle, S. , Maunder, M. , … Nicol, S. (2015). Using movement data from electronic tags in fisheries stock assessment: A review of models, technology and experimental design. Fisheries Research, 163, 152–160.

[ece33500-bib-0070] Stan Development Team (2013) Stan: A C++ library for probability sampling, Version 2.4.0. http://mc-stan.org/. Accessed 14 February 2016.

[ece33500-bib-0071] Suda, A. (1971). Tuna fisheries and their resources in the IFPC area (p. 58). Shizuoka, Shizuoka, Japan: Far Seas Fisheries Research Laboratory, S Series.

[ece33500-bib-0072] Sullivan, K. (2015) Discussion Paper on Future Scientific Research Programme. CCSBT‐ESC/1509/20.

[ece33500-bib-0073] Takahashi, N. , Tsuji, S. , & Kurota, H. (2004) Review of current CCSBT tagging program and potential improvements, CCSBT‐ESC/0409/36.

[ece33500-bib-0074] Warashina, Y. , & Hisada, K. (1974). Preliminary evaluation of effect of the voluntary regulation on stock of southern bluefin tuna and the longline fishery. Bulletin of Far Seas Research Laboratory, 10, 193–220. (In Japanese with English abstract).

[ece33500-bib-0075] Warashina, Y. , Nishikawa, Y. , Tsuji, S. , Ishizuka, Y. , & Suzuki, Z. (1989) Environmental conditions and formation of longline fishing grounds for southern bluefin tuna in the areas off South Africa. Indo‐Pacific Tuna Development and Management Program, IPTP/89/GEN/17, 143‐155.

