# Supplementary material for: Evidence of separate subgroups of juvenile southern bluefin tuna
Source: Ecol Evol. 2017 Nov 2;7(22):9818–44. doi: 10.1002/ece3.3500 (PMC5696402; doi:10.1002/ece3.3500)
Supplement: Supplementary file 1 [file ECE3-7-9818-s001.pdf]

Evidence of separate subgroups of juvenile southern bluefin tuna - supplementary material 1

Mark S. Chambers[1], Leesa A. Sidhu[1], Ben O'Neill[1], and Nokuthaba Sibanda[2]

[1]School of Physical, Environmental and Mathematical Sciences, University of New South Wales at the Australian Defence Force Academy, Canberra.
[2]School of Mathematics and Statistics, Victoria University of Wellington.

1 Historical depictions of SBT movement

In this supplement we provide previously published diagrams of proposed movement of southern bluefin tuna (SBT). The figure captions are reproduced verbatim from source documents.

The sizes/ages of SBT caught on different fishing grounds were important to theories of movement proposed by Shingu (1978). The arrangement of age histograms shown in Figure S1 illustrates ontogenetic movements of his theory. The ontogenetic movements specifically are represented by the solid arrow. Seasonal movements are represented by dashed arrows. As pointed out by Caton (1991), the ages assumed in Figure S1 which are derived from length measurements are not the same as would be currently assumed.

Based on the information in Figure S1 as well as other information, Shingu (1978), inferred movement characterised by Figure S2. This can be regarded as a simplified representation of what has been referred to as "The Traditional Model". It should be recognised that Figures S1 and S2 were intended to represent the migration of SBT across its full life time.

Figure S3, taken from Majkowski et al. (1988), shows proposed movements along the Australian coast as well as dispersion of juveniles into oceanic waters. A similar figure appears in Murphy & Majkowski (1981) which includes the assumed ages of the depicted movements and dispersion. Figure S3 can be regarded as a truncated representation of "The Alternative Model".

Figure S4 is used by Basson et al. (2012) to represent their model of juvenile migration. A similar diagram appears in Hobday et al. (2015).

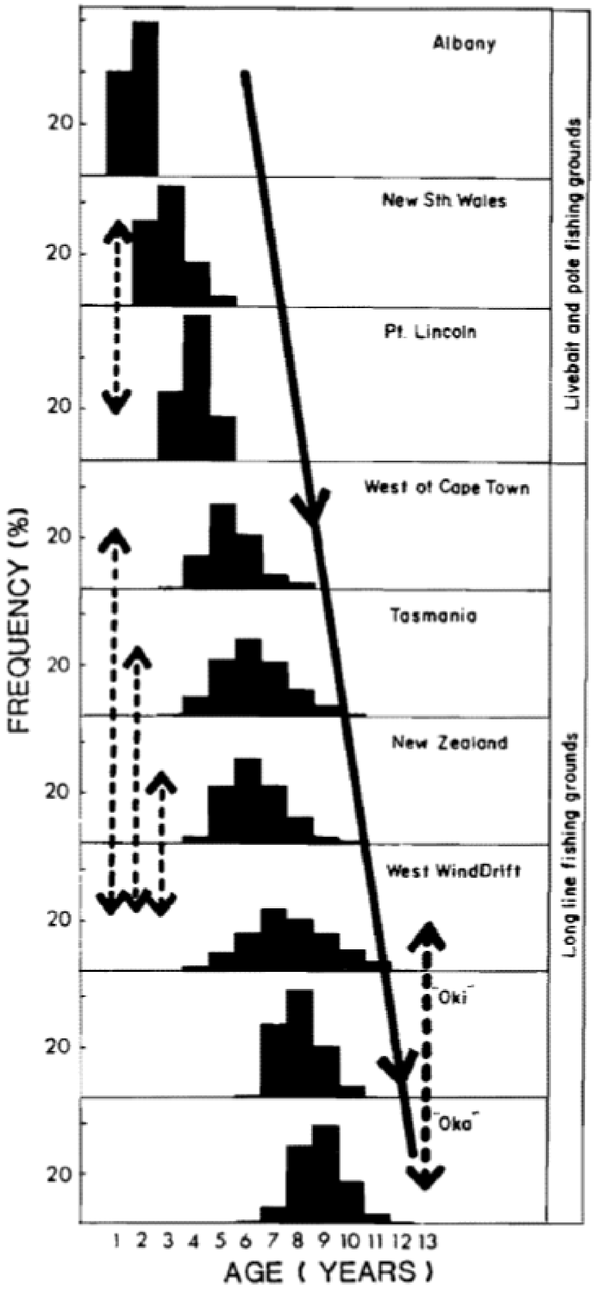

Figure S1: Change of distribution areas of southern bluefin tuna. Solid line: change during growth; Dotted line (thick): change during spawning and feeding; Dotted line (thin): seasonal change. Source: (Shingu 1978, his Figure 38).

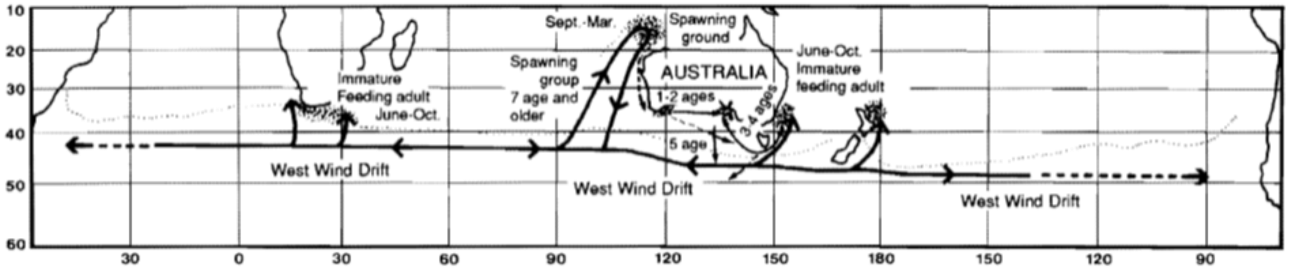

Figure S2: Schematic diagram of presumed course of migration of southern bluefin tuna. Source: (Shingu 1978, his Figure 39)

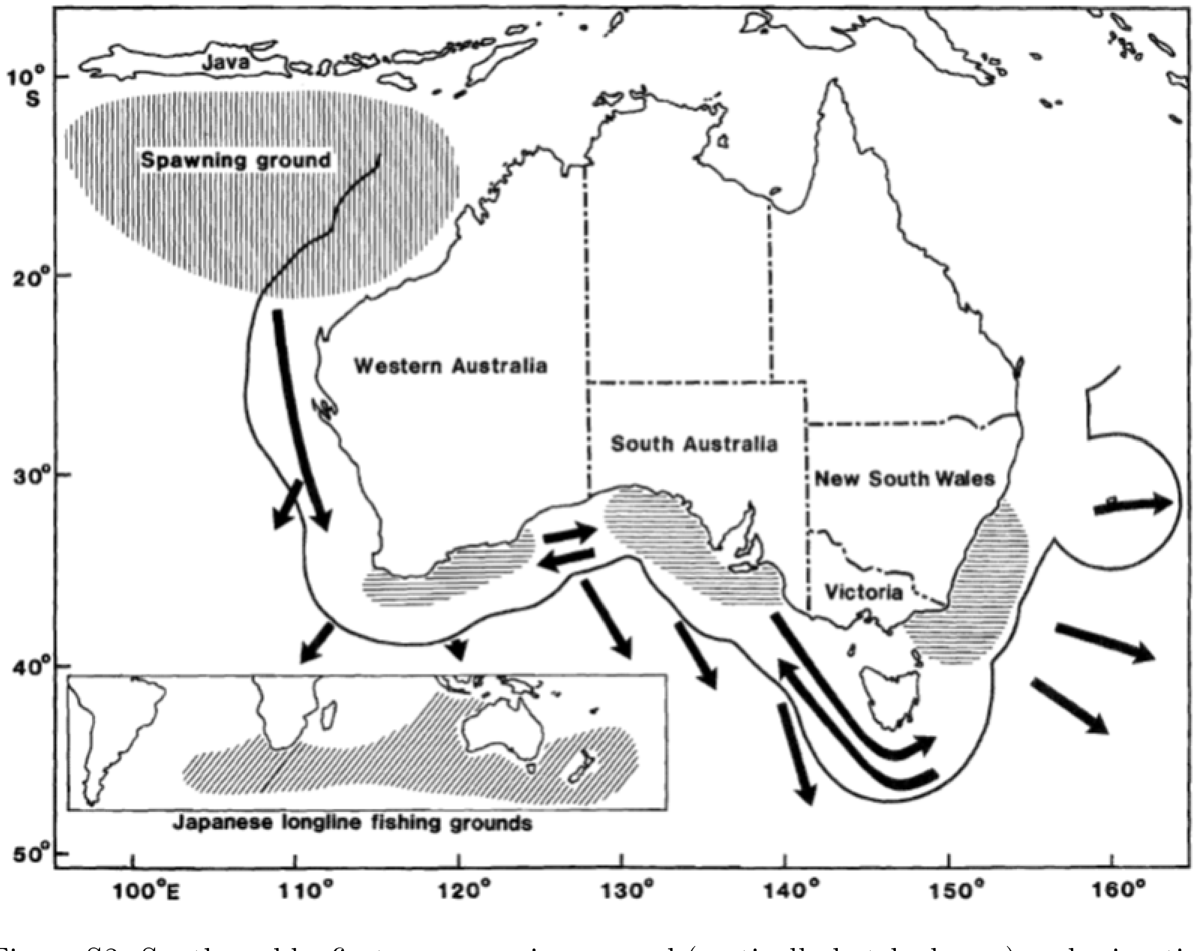

Figure S3: Southern bluefin tuna spawning ground (vertically hatched area) and migration pattern off the Australian coast (arrows indicate the primary direction of migration; the boundary of the 200-nautical-mile Australian fishing zone is indicated by the solid line) and the areas of the operation of the pre-1980 Australian fisheries (horizontally hatched areas) and the Japanese fishery (diagonally hatched area). Source: (Majkowski et al. 1988, their Figure 1)

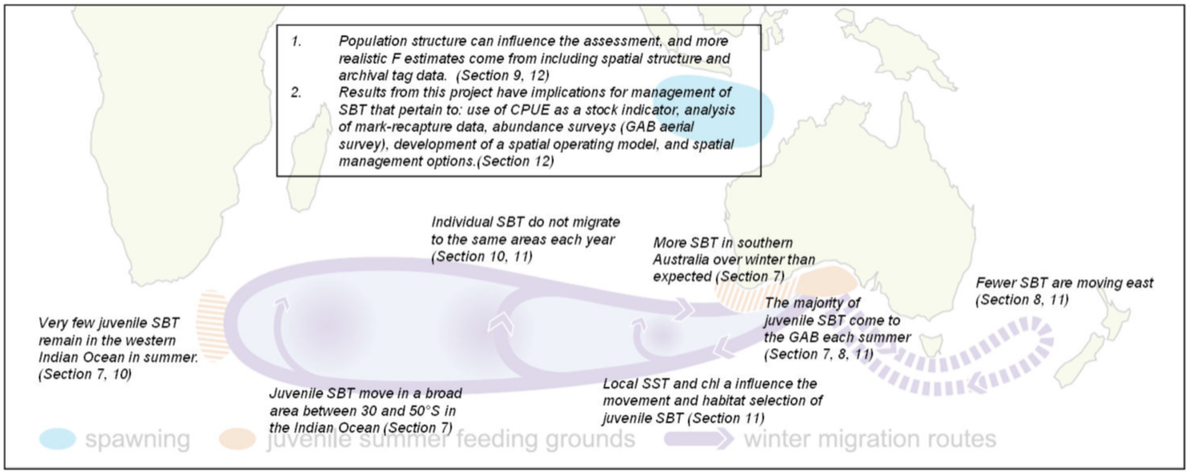

Figure S4: New insight into the movement and habitat use of juvenile (age 2-5) southern bluefin tuna throughout their range has resulted from this project. Juvenile SBT can occupy a broad area of the southern Indian Ocean, with several higher use areas. Fewer juveniles move to the Tasman Sea (dashed line). Some SBT are found in southern Western Australia in summer and winter. Source:(Basson et al. 2012, their Figure 16.1)

References

Basson, M., Hobday, A. J., Eveson, J. P. & Patterson, T. A. (2012), Spatial interactions among juvenile southern bluefin tuna at the global scale: a large scale archival tag experiment, Report 2003/002, Fisheries Research & Development Corporation.

Caton, A. (1991), Review of aspects of southern bluefin tuna biology, population and fisheries, *in* R. Deriso & W. Bayliff, eds, 'World Meeting on Stock Assessment of Bluefin Tunas: Strengths and Weaknesses', Vol. 3 of *Special Report*, Inter-American Tropical Tuna Commission, pp. 181–357.

Hobday, A. J., Evans, K., Eveson, J. P., Farley, J. H., Hartog, J. R., Basson, M. & Patterson, T. A. (2015), Distribution and migration - southern bluefin tuna (*Thunnus maccoyii*), *in* T. Kitagawa & S. Kimura, eds, 'Biology and ecology of bluefin tuna', CRC Press.

Majkowski, J., Hearn, W. & Sandland, R. (1988), 'A tag-release/recovery method for predicting the effect of changing the catch of one component of a fishery upon the remaining components.', *Canadian Journal of Fisheries and Aquatic Science* **45**, 675–684.

Murphy, G. I. & Majkowski, J. (1981), 'State of the southern bluefin tuna population: fully exploited', *Australian Fisheries* **40**, 20–29.

Shingu, C. (1978), Ecology and stock of southern bluefin tuna, Fisheries Study 31, Japan Association of Fishery Resources Protection. [In Japanese. English Translation in the form of CSIRO. Div. Fish. Oceanogr. Rep. No. 131, 79 pp. 1981].
