# Supplementary material for: Evidence of separate subgroups of juvenile southern bluefin tuna
Source: Ecol Evol. 2017 Nov 2;7(22):9818–44. doi: 10.1002/ece3.3500 (PMC5696402; doi:10.1002/ece3.3500)
Supplement: Supplementary file 2 [file ECE3-7-9818-s002.pdf]

Evidence of separate subgroups of juvenile southern bluefin tuna - supplementary material 2

Mark S. Chambers[1], Leesa A. Sidhu[1], Ben O'Neill[1], and Nokuthaba Sibanda[2]

[1]School of Physical, Environmental and Mathematical Sciences, University of New South Wales at the Australian Defence Force Academy, Canberra.
[2]School of Mathematics and Statistics, Victoria University of Wellington.

1 Surface fishery tag recoveries

In this section we describe two alternative Bayesian models for the numbers of surface fishery recoveries of one-year-old southern bluefin tuna (SBT) tagged and released in years $y \in \{1959, \cdots, 2007\}$ from three locations, $l \in \{\mathrm{WA}, \mathrm{SA}, \mathrm{EA}\}$, off southern and eastern Australia. Numbers of surface fishery recoveries observed are modelled as binomial random variables in terms of known numbers of tag releases because we are interested in the probability of surface fishery recovery. Specifically, we are interested in whether the probability of surface fishery recovery of one-year-old SBT tagged in a given year differs by tagging location.

We first describe the assumed model in Section 1.1 including model specification, posterior parameter summaries and diagnostics, and then, in Section 1.2, provide the same information for a reduced model. The characteristics of the assumed model and the goodness of fit of the assumed model compared with the reduced model is intended to provide information on the possibility of differences in surface fishery recovery probabilities among tagging states. Tags recaptured in the same surface fishing season as release are excluded from these analyses.

1.1 Assumed model for surface fishery recoveries

Let the number of one-year-old southern bluefin tuna (SBT) tagged and released from location l in year y that were not recovered from the Australian surface fishery in the same fishing season as release be denoted T_{ly}. We assume S_{ly}, the number of surface fishery recoveries observed from the T_{ly} released, is the realisation of a binomial random variable with probability π_{ly}. Probability of tag recovery, π_{ly}, is allowed to vary by tagging location and year of release. The full model is specified as:

$$\begin{aligned} S_{ly} &\sim \text{Binomial}(T_{ly}, \pi_{ly}), \qquad (1) \\ \text{logit}(\pi_{ly}) &= \log\left(\frac{\pi_{ly}}{1-\pi_{ly}}\right) = \beta_l + \delta_{ly}, \\ \delta_{ly} &= \phi_l \times \delta_{l(y-1)} + \epsilon_{ly}, \\ \epsilon_{ly} &\sim N(0, \sigma_l^2). \end{aligned}$$

The priors for model (1) are defined as:

$$\begin{aligned}
&\beta_l, \delta_{l,1959} \sim N(\text{mean} = 0, \text{std. dev.} = 10),\\
&\phi_l \sim N(\text{mean} = 0, \text{std. dev.} = 2),\\
&\sigma_l \sim \text{half} - \text{Cauchy}(\text{scale} = 5).
\end{aligned}$$

The tagging-location-specific year effects, δ_{ly}, were each assumed to follow an AR(1) autoregressive process. The full series of year effects, δ_{ly}, corresponding to each release location, l, was constrained to have a mean of zero so that the parameters, β_l were better determined. However, tags were not released from all locations in all years. This results in autocorrelated δ_{ly} that can wander around during periods where no tags were released, limiting the precision of posterior estimates of the β_l.

The model was fitted using Stan (Stan Development Team 2014).

1.1.1 Posterior distributions of model parameters

Summaries of posterior distributions of key parameters from Model (1) are given in Table S1. The quantities N_{eff} and $\hat{R}$ describe characteristics of the HMC sequence used to approximate the posterior distributions rather than the distributions themselves. The N_{eff} statistics are the estimated "effective sizes" of the HMC sample, a measure of the information content of the sequences expressed in terms of the equivalent number of independent samples. The $\hat{R}$ statistics are the "potential scale reduction factors" (Gelman & Rubin 1992) convergence diagnostic, comparing between chain and within chain variances. Values of $\hat{R}$ near unity are consistent with convergence of the HMC algorithm used to approximate the posterior distributions of the parameters.

Table S1: Posterior parameter summaries for the assumed binomial Model (1) for surface fishery recoveries of SBT. Tags recovered in the same fishing season as release were excluded from the analysis.

Parameter	Mean	Std. Dev.	95% Cred. Int.	N_{eff}	$\hat{R}$
β_{SA}	-2.47	0.28	(-3.09, -1.98)	4.4×10^4	1
β_{WA}	-3.10	0.19	(-3.52, -2.74)	7.1×10^4	1
β_{EA}	-1.90	2.39	(-6.67, 3.29)	1.2×10^4	1
ϕ_{SA}	0.64	0.19	(0.16, 0.97)	1.4×10^4	1
ϕ_{WA}	0.49	0.30	(-0.17, 0.97)	1.2×10^4	1
ϕ_{EA}	0.65	0.32	(-0.09, 1.05)	1.1×10^4	1
σ_{SA}	0.59	0.15	(0.37, 0.95)	3.4×10^4	1
σ_{WA}	0.83	0.15	(0.59, 1.16)	6.2×10^4	1
σ_{EA}	1.08	0.30	(0.65, 1.81)	2.6×10^4	1

Summaries of the year effects, δ_{ly}, from Model (1) are not included in Table S1. Instead, posterior estimates of surface fishery recovery of one-year-olds released each year according to this model are summarised in Figure S5. Although Model 1 provides posterior estimates of π_{ly} in years where no tags were released, we plot the distributions only for years when one-year-olds were tagged and released. It can be seen that despite posterior variance in

α, β_{WA} and β_{EA}, posterior estimates of π_{ly} are usually quite precise in years where data are available depending largely on the number of tags released from a given location, l, in a given year, y (Figure S5).

Posterior estimates of surface fishery recovery probability of one-year-olds released from WA (Figure S5a) are generally higher than of one-year-olds released from SA (Figure S5b) during the 2000s and the 1960s. Larger differences in probability of surface fishery recovery between tags released from EA (Figure S5c) and SA are estimated during the late 1960s. For example, one-year-olds tagged and released from EA during 1967 and 1968 are estimated to have had probabilities of greater than 0.3 and 0.4 respectively of being recovered by a surface fishery. In contrast, tags released from SA in the same years are estimated to have had probabilities less than 0.1 of being recovered by the surface fisheries.

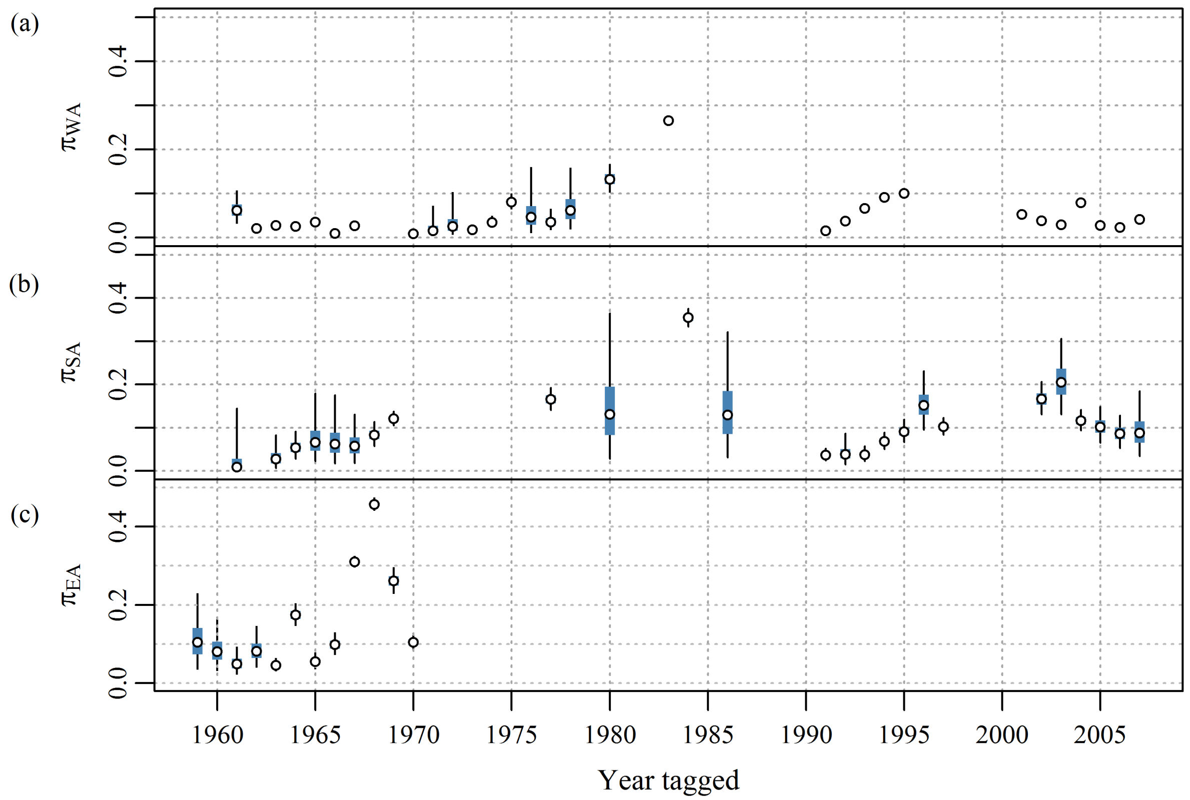

Figure S5: Posterior distributions of probability of surface fishery recovery of one-year-old SBT tagged and released annually from (a) WA, (b) SA and (c) EA according to Model (1). The boxes indicate posterior 50% credible intervals and the thin vertical lines are 95% credible intervals. Open circles are posterior medians of surface fishery recovery probability.

1.1.2 Realised Pearson residuals

Given the numbers of surface fishery recoveries, S_{ly}, the numbers of releases, T_{ly}, and the probabilities of surface fishery recovery, π_{ly}, the unstandardised Pearson or chi-squared residuals (see e.g. Collett 2002), e_{ly}, are:

$$e_{ly} = \frac{S_{ly} - T_{ly}\pi_{ly}}{\sqrt{T_{ly}\pi_{ly}(1 - \pi_{ly})}}.$$

The estimated recovery probabilities, π_{ly}, and therefore the residuals, e_{ly}, are conditional on the model parameters which we take to be random variables. Our belief about the parameter values is defined by their posterior distribution which we denote $\boldsymbol{\theta}$ and approximate with 300,000 HMC samples. We denote a single draw from the posterior $\boldsymbol{\theta}_q$. Recovery probabilities conditional on $\boldsymbol{\theta}_q$ are denoted π_{lyq} and the corresponding residuals e_{lyq}, with:

$$e_{lyq} = \frac{S_{ly} - T_{ly}\pi_{lyq}}{\sqrt{T_{ly}\pi_{lyq}(1 - \pi_{lyq})}}.$$

A Bayesian residual graph plots a single realisation of the residuals (i.e. based on a single draw $\boldsymbol{\theta}_q$) (Gelman et al. 2004, p. 170). We provide Bayesian Pearson residual graphs as an analog for binomial models (Figure S6). Realisations of Pearson residuals from three random draws from the posterior are included in Figure S6 and residuals from each tag release location are shown separately to provide extra information.

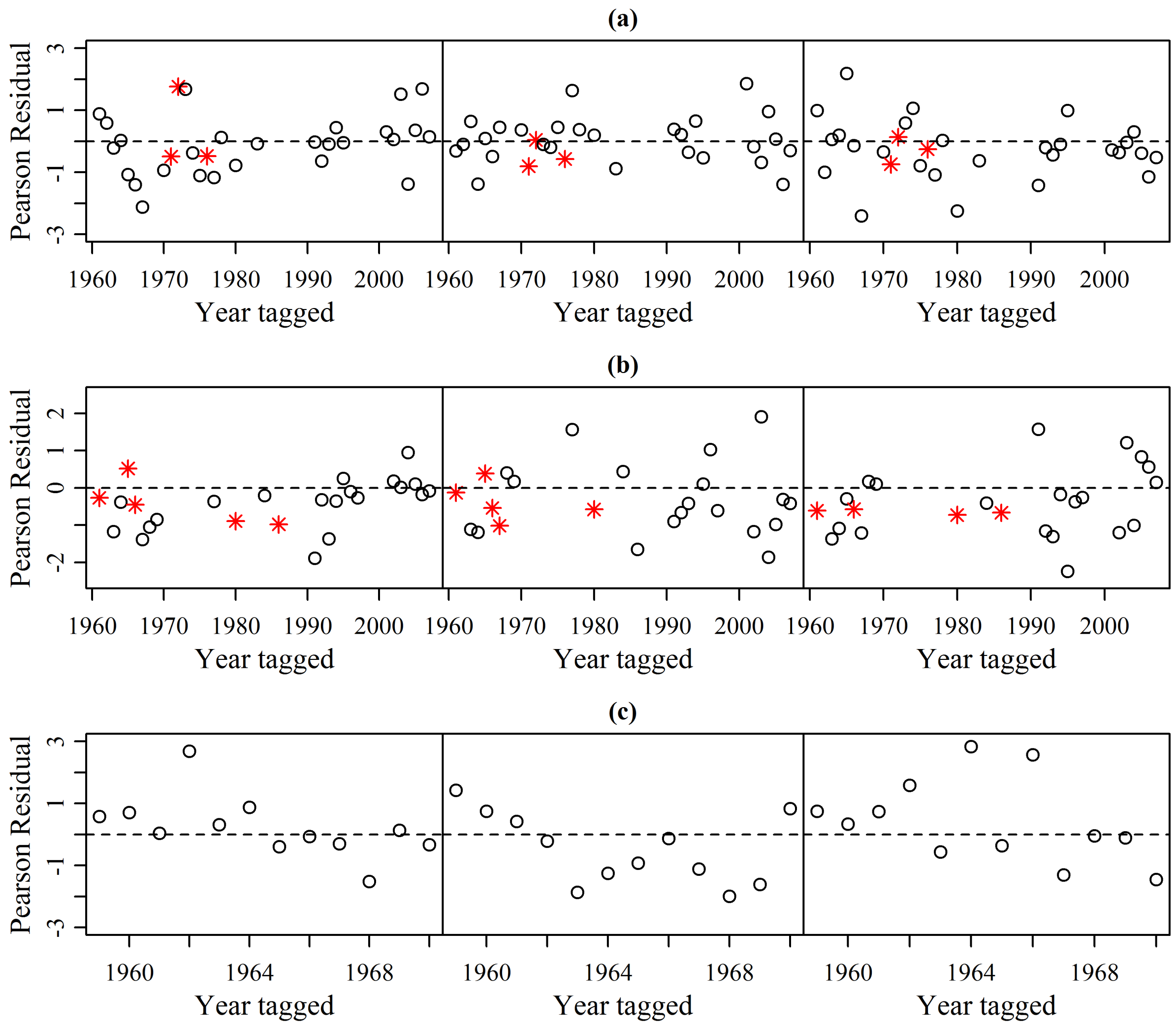

Figure S6: Realised Pearson residuals of number of surface fishery recoveries of one-year-olds tagged and released annually from (a) WA, (b) SA and (c) EA assuming Model (1). Three sets of realised residuals are plotted from each tag release location corresponding to three random draws from the posterior distribution of the parameters of Model (1). The red asterisks denote residuals associated with observations for which the conditionally expected numbers of surface fishery recoveries are less than one.

1.1.3 Posterior predictive checks

The goodness of fit of Bayesian models can be assessed by comparing the posterior distribution of realised "discrepancies" with the discrepancies of the posterior predictive distribution (Gelman et al. 2004). These comparisons form the basis of posterior p-values and discrepancy plots.

Following Brooks et al. (2000), we measure model discrepancy using the Freeman-Tukey statistic (Freeman & Tukey 1950). Brooks et al. (2000) note that the Freeman-Tukey statistic is more convenient than the chi-squared statistic when small numbers of observations occur at some combinations of the predictors. Here we randomly sample $Q = 50,000$ draws from the posterior $\boldsymbol{\theta}$.

For each posterior draw, $\boldsymbol{\theta}_q$, we calculate the corresponding set of recapture probabilities, π_{lyq}, as specified in Model (1). The realised discrepancies are then calculated as:

$$D(S; \boldsymbol{\theta}_q) = \sum_l \sum_y (\sqrt{S_{ly}} - \sqrt{T_{ly} \times \pi_{lyq}})^2.$$

Given π_{lyq}, we simulate a set of posterior predictive recoveries, $S_{lyq} \sim \text{Binomial}(T_{ly}, \pi_{lyq})$. The simulated discrepancies are then calculated as:

$$D(S_q; \boldsymbol{\theta}_q) = \sum_l \sum_y (\sqrt{S_{lyq}} - \sqrt{T_{ly} \times \pi_{lyq}})^2.$$

Having saved the set of Q realised and simulated discrepancies, posterior predictive p-values (see Meng 1994) can be estimated as:

$$\text{p} - \text{value} = \frac{1}{Q} \sum_{q=1}^{Q} I(D(S_q; \boldsymbol{\theta}_q) \geq D(S; \boldsymbol{\theta}_q),$$

where $I(\cdot)$ denotes an indicator function that takes the value of unity when the condition is satisfied and zero otherwise.

The standard practice of examining the discrepancies of a fitted model as a whole can be extended to examine different aspects of model fit separately. In this case we calculate discrepancies resulting from predicted recoveries of tags released from each tagging location separately. Simulated versus realised discrepancies for each tagging location for 2000 random draws from the posterior distribution are shown in Figure S7. Posterior predictive p-values (based on 50,000 draws from the posterior) corresponding to releases from each tagging location are provided on the same figure.

The posterior p-values for releases from each of the three tagging locations are quite near 0.5 (Figure S7) suggesting the observed data are typical of what would be expected if Model (1) were true.

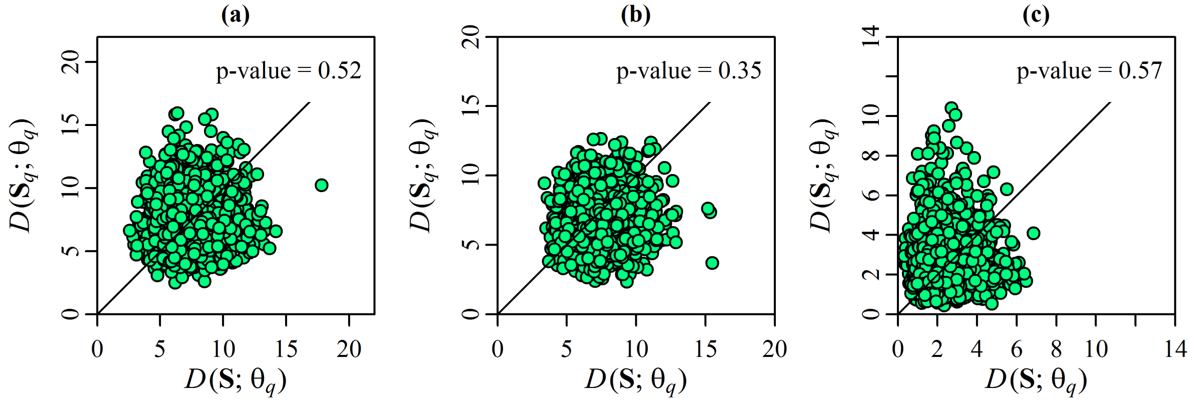

Figure S7: Freeman-Tukey discrepancy plots generated from 2000 random draws from the posterior of Model (1). Simulated versus realised discrepancies are plotted separately for tag releases from (a) WA, (b) SA and (c) EA. Posterior predictive p-values for each tag release location are shown in the top right corners.

1.2 Reduced model for surface fishery recoveries

If most juvenile SBT summer in the Great Australian Bight (GAB) and then mix thoroughly, it would be expected that subsequent to their first summer after tag and release, probabilities of surface fishery recovery of tagged one-year-olds would be independent of tagging location. To test this hypothesis we define a reduced model for surface fishery recovery of one-year-olds that, like Model (1), allows probability of surface fishery recovery to vary by year of release, but, unlike Model (1), assumes that releases from all three locations in a given year, y, have a common recovery probability, π_y.

Let T_y be the total number of one-year-old SBT tagged and released from WA, SA and EA in year y and not recovered in the same fishing season and let S_y be the number of these that are recovered by the surface fishery in a subsequent fishing season. We consider the possibility that S_y is the realisation of a binomial random variable with recovery probability, π_y. This reduced model is specified as:

$$\begin{aligned} &S_y \sim \text{Binomial}(T_y, \pi_y), \\ &\text{logit}(\pi_y) = \log\left(\frac{\pi_y}{1-\pi_y}\right) = \beta + \delta_y, \\ &\delta_y = \phi \times \delta_{(y-1)} + \epsilon_y, \\ &\epsilon_y \sim N(0, \sigma^2). \end{aligned} \tag{1*}$$

The priors for Model (1*) are defined as:

$$\begin{aligned} &\beta,\ \delta_{1959} \sim N(\text{mean} = 0, \text{std. dev.} = 10), \\ &\phi \sim N(\text{mean} = 0, \text{std. dev.} = 2), \\ &\sigma \sim \text{half} - \text{Cauchy}(\text{scale} = 5). \end{aligned}$$

Note that the reduced Model (1*) is equivalent to Model (1) except the effects allowing differences in recovery probability by tag release location have been removed. As with Model (1), probability of surface fishery recovery of one-year-olds tagged each year is assumed to follow an AR(1) process, but since no differences due to tagging state are assumed, the year effects in this case are shared by releases from the three tagging locations. We fitted the reduced model using Stan for comparison with the assumed Model (1).

1.2.1 Posterior distributions of model parameters

Posterior summaries of key Model (1*) parameters are provided in Table S2.

Posterior probabilities of surface fishery recovery, π_y, of one-year-olds released in year y according to Model (1*) are plotted in Figure S8. These distributions include the year effects, δ_y, not summarised in Table S4. Years in which no tags were released are excluded from this plot.

Table S2: Posterior parameter summaries for the reduced binomial Model (1*) for surface fishery recoveries of SBT tagged and released as one-year-olds from WA, SA and EA between 1959 and 2007. Recovered tags recaptured in the same fishing season as release were excluded from the analysis.

Parameter	Mean	Std. Dev.	Cred. Int.	N_{eff}	$\hat{R}$
β	-2.59	0.1	(-2.79, -2.40)	7.1×10^4	1
ϕ	0.68	0.16	(0.35, 0.99)	1.0×10^4	1
σ	0.77	0.11	(0.60, 1.02)	6.1×10^4	1

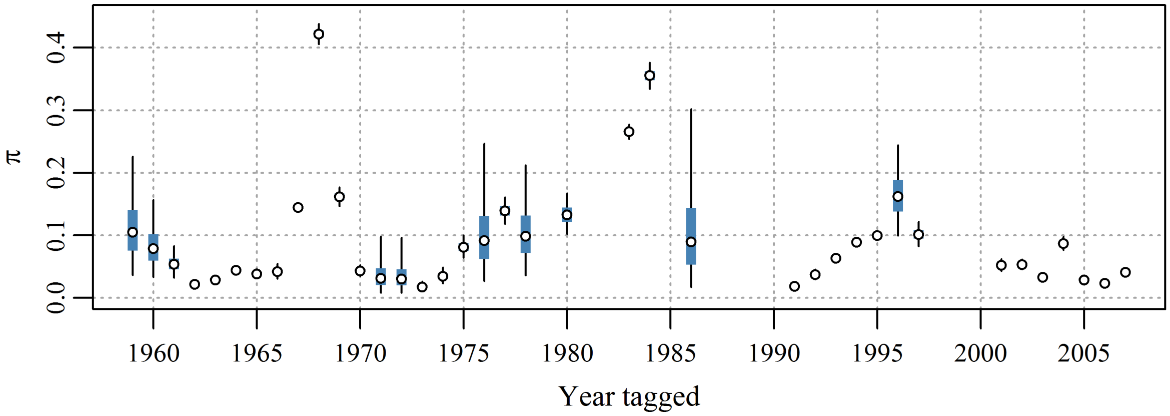

Figure S8: Posterior distributions of probability of surface fishery recovery of one-year-old SBT tagged and released annually from any of WA, SA or EA according to Model (1*). The boxes indicate posterior 50% credible intervals and the thin vertical lines are 95% credible intervals. Open circles are posterior medians of surface fishery recovery probability.

1.2.2 Realised Pearson residuals

The reduced model tends to overpredict the number of recoveries from WA releases resulting in large negative residuals in some years (Figure S9a). The large negative residuals mostly correspond to tag releases during the 1960s. Pearson residuals realised from tag releases from WA during the 2000s, whilst less extreme, also tend to be negative. Residuals corresponding to releases from EA tend to be positive (Figure S9c) and are extreme in some years.

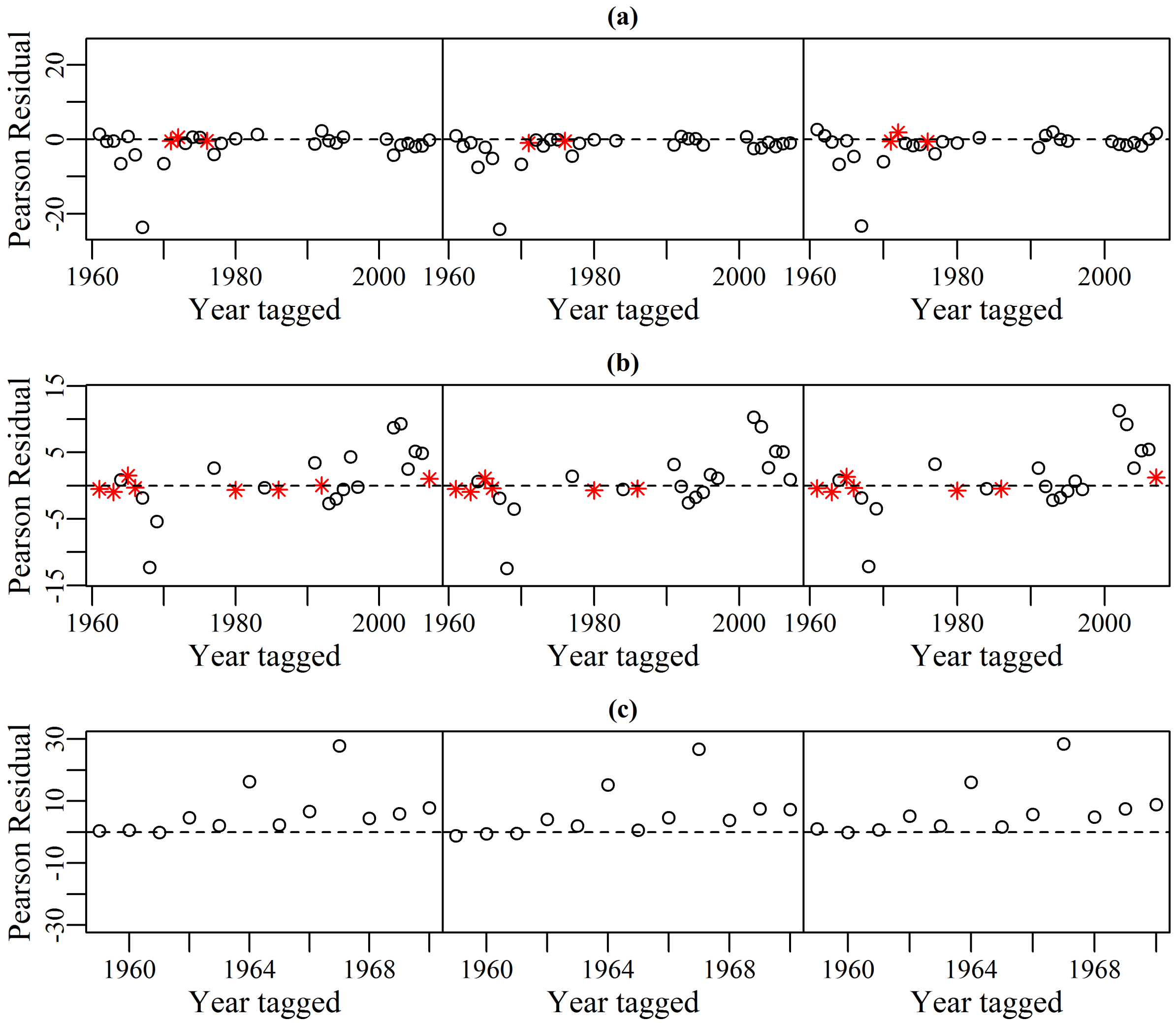

Figure S9: Realised Pearson residuals of number of surface fishery recoveries of SBT tagged and released annually from (a) WA, (b) SA and (c) EA assuming Model (1*). Three sets of realised residuals are plotted from each tag release location corresponding to three random draws from the posterior distribution of the parameters of Model (1*). The red asterisks denote residuals associated with observations for which the conditionally expected numbers of surface fishery recoveries are less than one.

In contrast with Figure S6, it is evident that the major features in Figure S9, such as the extreme negative residuals realised for releases from WA in 1967 (Figure S9a) and from SA in 1968 (Figure S9b), tend to be consistent across the rows. This suggests these are due to an inadequate fit of the reduced model to the observed data rather than posterior parameter uncertainty.

1.2.3 Posterior predictive checks

The lack of fit apparent in the Bayesian residual graphs (Figure S9) is shown more clearly in discrepancy plots (Figure S10). The discrepancy plots show that Model (1*) is very definitely inadequate. Simulated discrepancies never approach realised discrepancies and consequently the posterior p-values from all tag release locations are zero.

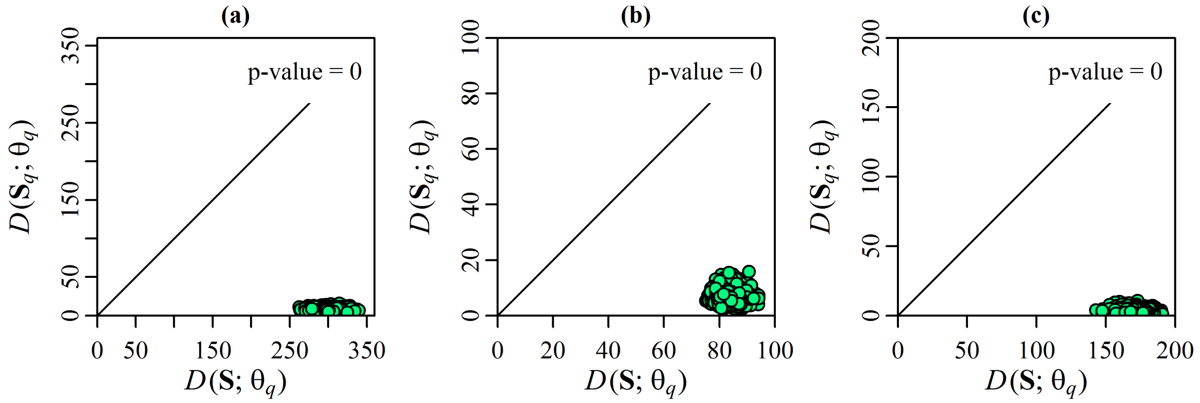

Figure S10: Freeman-Tukey discrepancy plots generated from 2000 random draws from the posterior of Model (1*). Simulated versus realised discrepancies are plotted separately for tag releases from (a) WA, (b) SA and (c) EA. Posterior predictive p-values for each tag release location are shown in the top right corners.

2 Recapture location of longline recovery

In this section we describe two alternative hierarchical Bayesian models for the number of recoveries in each of the three longline fisheries that we define (Western LL, Central LL and Eastern LL) from cohorts spawned between 1958 and 2006. In each case the observed numbers of recoveries from each cohort observed in the three longline fisheries are modelled using using a two stage approach.

The first stage models the probability that a fish from a given cohort tagged in a given location and recovered from a longline vessel is recaptured east of 120°E. The second stage models the probability a fish spawned in a particular year, tagged in a particular location, and recovered by longline not recaptured east of 120°E is recaptured west of 60°E. It is straightforward within a Bayesian framework to combine these two submodels to produce a multinomial model for longline recapture location.

We first describe the assumed model in Section 2.1 and then, in Section 2.2, provide the same information for a reduced model. The characteristics of the assumed model and the goodness of fit of the assumed model compared with the reduced model is intended to provide information on the possibility of differences in the distribution of longline recovery locations of individual cohorts by tagging state.

2.1 Assumed model for location of longline recovery

2.1.1 Binomial submodel for recapture in Eastern LL

Let L_{ly} be the total number of longline recoveries of SBT spawned in year $y \in \{1958, \cdots, 2006\}$ and tagged in tagging location, l, and let Z_{ly} be the number of these that were recaptured east of 120°E. We assume the Z_{ly} are binomial random variables with probabilities P_{ly_a}, described by:

$$
\begin{aligned}
&Z_{ly} \sim \text{Binomial}(L_{ly}, P_{ly_a}), \qquad (2)\\
&\text{logit}(P_{ly_a}) = \log\left(\frac{P_{ly_a}}{1 - P_{ly_a}}\right) = \mu_{l_a} + \omega_{ly_a},\\
&\omega_{ly_a} = \kappa_a \times \omega_{l(y-1)_a} + \epsilon_{ly_a},\\
&\epsilon_{ly_a} \sim N(0, \sigma^2_{l_a}).
\end{aligned}
$$

The priors for model (2) are defined as:

$$
\begin{aligned}
&\mu_{l_a}, \omega_{l\,1958_a} \sim N(\text{mean} = 0, \text{std. dev.} = 10),\\
&\kappa_a \sim N(\text{mean} = 0, \text{std. dev.} = 2),\\
&\sigma_{l_a} \sim \text{half} - \text{Cauchy}(\text{scale} = 5).
\end{aligned}
$$

The parameters μ_l are contrasts for releases from WA and EA respectively. The tagging-location-specific year effects are constrained to average zero and share a common AR(1) coefficient, κ. Separate autoregressive coefficients were originally fitted, but they were not found to be very different from one another.

2.1.2 Binomial submodel for recapture in Western LL given west of 120°E

Let the number of SBT spawned in year y tagged and released from location l at age three or below and recovered by longline recapture west of 120°E be X_{ly}. We assume W_{ly}, the number of longline recoveries of X_{ly} occuring west of 60°E, is the realisation of a binomial random variable with probability P_{ly_b}. Probability of tag recovery, P_{ly_b}, is allowed to vary by tagging location and year of release.

$$
\begin{aligned}
&W_{ly} \sim \text{Binomial}(X_{ly}, P_{ly_b}), \\
&\text{logit}(P_{ly_b}) = \log\left(\frac{P_{ly_b}}{1 - P_{ly_b}}\right) = \mu_{l_b} + \omega_{ly_b}, \\
&\omega_{ly_b} = \kappa_b \times \omega_{l(y-1)_b} + \epsilon_{ly_b}, \\
&\epsilon_{ly_b} \sim N(0, \sigma^2_{l_b}).
\end{aligned} \tag{3}
$$

The priors for model (3) are defined as:

$$
\begin{aligned}
&\mu_{l_b}, \omega_{l\,1958_b} \sim N(\text{mean} = 0, \text{std. dev.} = 10), \\
&\kappa_b \sim N(\text{mean} = 0, \text{std. dev.} = 2), \\
&\sigma_{l_b} \sim \text{half} - \text{Cauchy}(\text{scale} = 5).
\end{aligned}
$$

2.1.3 Combined multinomial model for longline recapture location

The two binomial submodels can together be alternatively interpreted as a multinomial model for recapture location conditional on longline recovery.

We have already defined the number of recoveries from Eastern LL, Z_{ly}, the number of recoveries from Western LL, W_{ly}, and the total number of longline recoveries L_{ly}. Let the number of recoveries from Central LL be C_{ly}, where $C_{ly} = L_{ly} - Z_{ly} - W_{ly}$. If we define the vector $\vec{V_{ly}} = [W_{ly}, C_{ly}, E_{ly}]^T$, then we assume:

$$
\vec{V_{ly}} \sim \text{Multinomial}(L_{ly}, \vec{P_{ly}}) \tag{4}
$$

where $\vec{P_{ly}} = [(1 - P_{ly_a})P_{ly_b}, (1 - P_{ly_a})(1 - P_{ly_b}), P_{ly_a}]^T$ and P_{ly_a} and P_{ly_b} are as defined in submodels 2 and 3.

2.1.4 Posterior distributions of model parameters

Posterior distributions of parameters of submodels (2) and (3) parameters are provided in the top and bottom halves respectively of Table S3.

Table S3: Posterior parameter summaries for the assumed binomial Submodels 2 and 3 for recapture ground conditional on longline fishery recovery, by year spawned, of SBT tagged and released in WA, SA and EA. Parameters with a subscripts pertain to submodel 2 and parameters with b subscripts pertain to submodel 3.

Parameter	Mean	Std. Dev.	95% Cred. Int.	N_{eff}	$\hat{R}$
μ_{SA_a}	0.06	0.24	(-0.41, 0.56)	5.6×10^4	1
μ_{WA_a}	-0.68	0.3	(-1.27, -0.08)	4.7×10^4	1
μ_{EA_a}	4.09	1.81	(1.63, 13.3)	2.5×10^4	1
κ_a	0.82	0.10	(0.64, 1.00)	6.4×10^3	1
σ_{SA_a}	0.64	0.14	(0.42, 0.97)	3.5×10^4	1
σ_{WA_a}	0.75	0.20	(0.44, 1.23)	2.7×10^4	1
σ_{EA_a}	1.54	1.41	(0.10, 5.21)	1.0×10^4	1
μ_{SA_b}	-0.18	0.29	(-1.17, 0.18)	3.9×10^4	1
μ_{WA_b}	0.93	0.45	(0.09, 0.42)	4.0×10^4	1
μ_{EA_b}	-0.83	7.60	(-16.5, 14.9)	1.8×10^4	1
κ_b	0.93	0.10	(0.65, 1.01)	1.6×10^4	1
σ_{SA_b}	0.40	0.16	(0.16, 0.78)	1.1×10^4	1
σ_{WA_b}	0.73	0.22	(0.39, 1.38)	2.3×10^4	1
σ_{EA_b}	38.9	136.4	(2.12, 231)	2.5×10^3	1

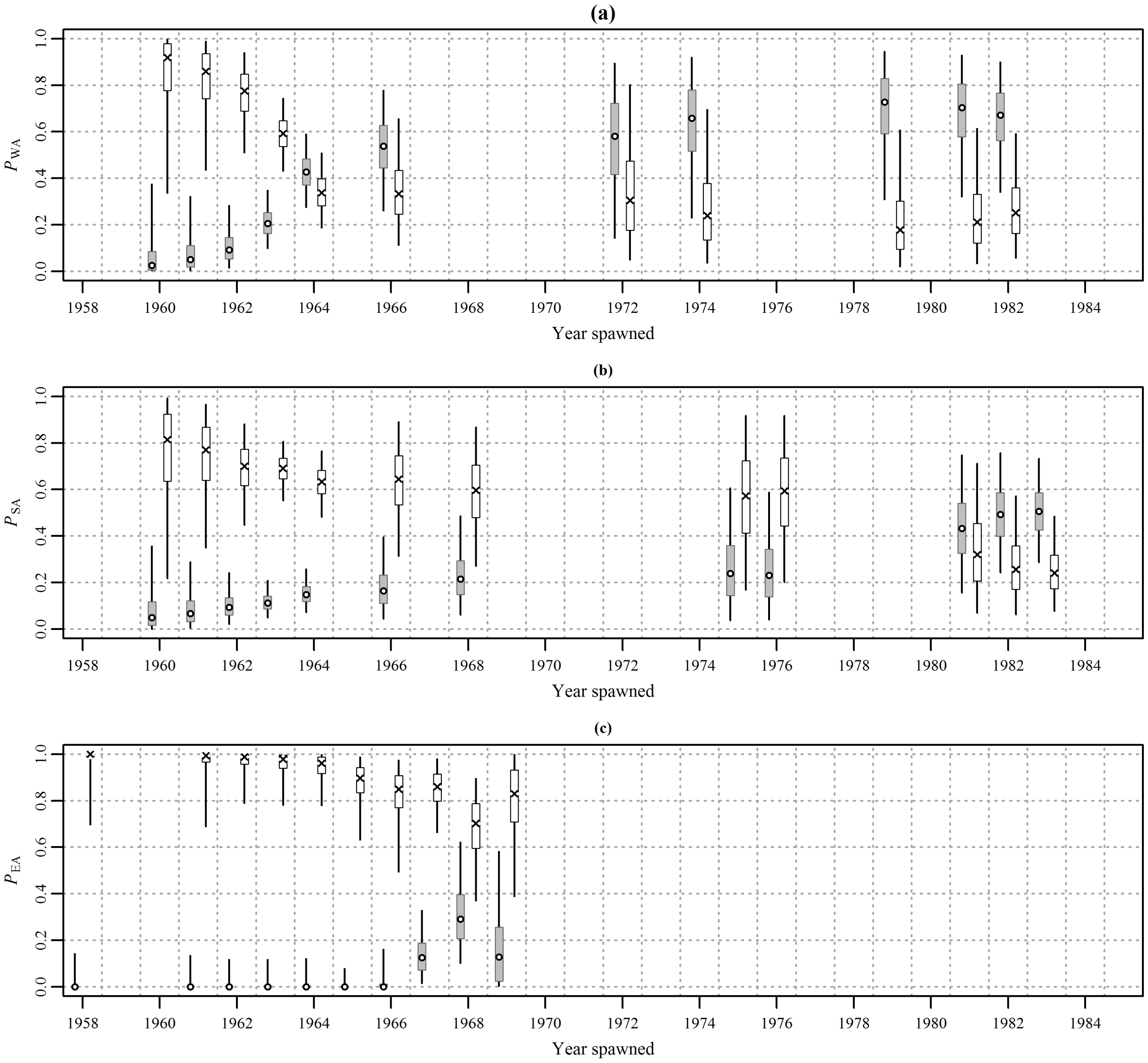

Figure S11: Posterior distributions of probability of recapture east of 120°E (crosses and white boxes) and west of 60°E (open circles and grey boxes) conditional on longline recovery of cohorts of spawned before 1985 and tagged and released from (a) WA, (b) SA and (c) EA according to Model (2). The boxes indicate posterior 50% credible intervals and the thin vertical lines are 95% credible intervals. Crosses and open circles are posterior medians.

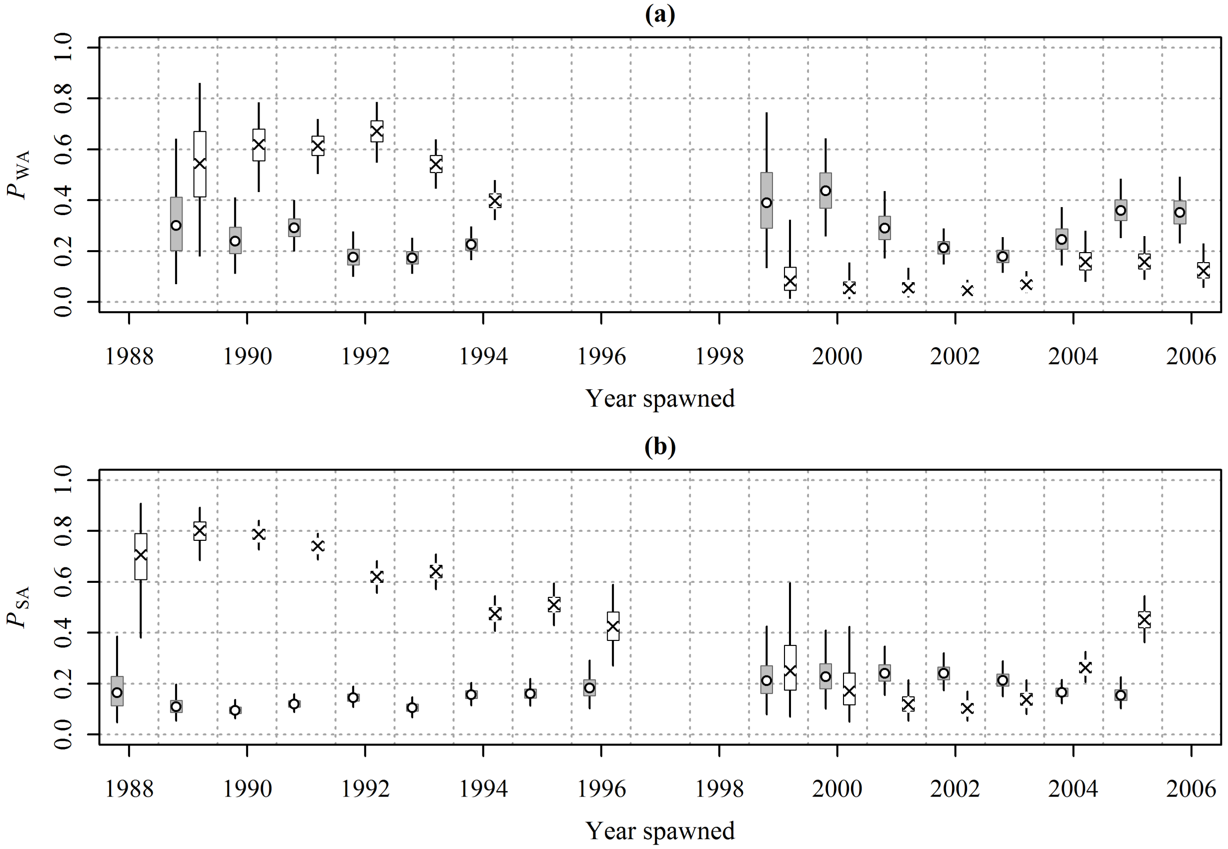

Figure S12: Posterior distributions of probability of recapture east of 120°E (crosses and white boxes) and west of 60°E (open circles and grey boxes) conditional on longline recovery of cohorts of spawned since 1987 and tagged and released from (a) WA and (b) SA described by Submodels 2 and 3. The boxes indicate posterior 50% credible intervals and the thin vertical lines are 95% credible intervals. Crosses and open circles are posterior medians.

2.1.5 Realised Pearson residuals for longline recovery locations

This time we provide realised Pearson residuals for each Submodel 2 and 3. We define the realised residual from the qth draw from the posterior of submodel 2 as:

$$e_{lyq_a} = \frac{Z_{ly} - L_{ly}P_{lyq_a}}{\sqrt{L_{ly}P_{lyq_a}(1 - P_{lyq_a})}}.$$

Three realisations of residuals by tagging location are plotted in Figure S13.

Consistent with earlier, we define the conditional probability of recapture in Western LL of a tagged SBT spawned in year y and tagged in location l is defined as $P_{ly}^{W} = (1 - P_{ly_a}) \times P_{ly_b}$ The realised residual from the qth draw from the posterior of submodel 3 is defined as:

$$e_{lyq_b} = \frac{W_{ly} - L_{ly}P_{ly}^{W}}{\sqrt{L_{ly}P_{ly}^{W}(1 - P_{ly}^{W})}}.$$

Three realisations of residuals by tagging location are plotted in Figure S14.

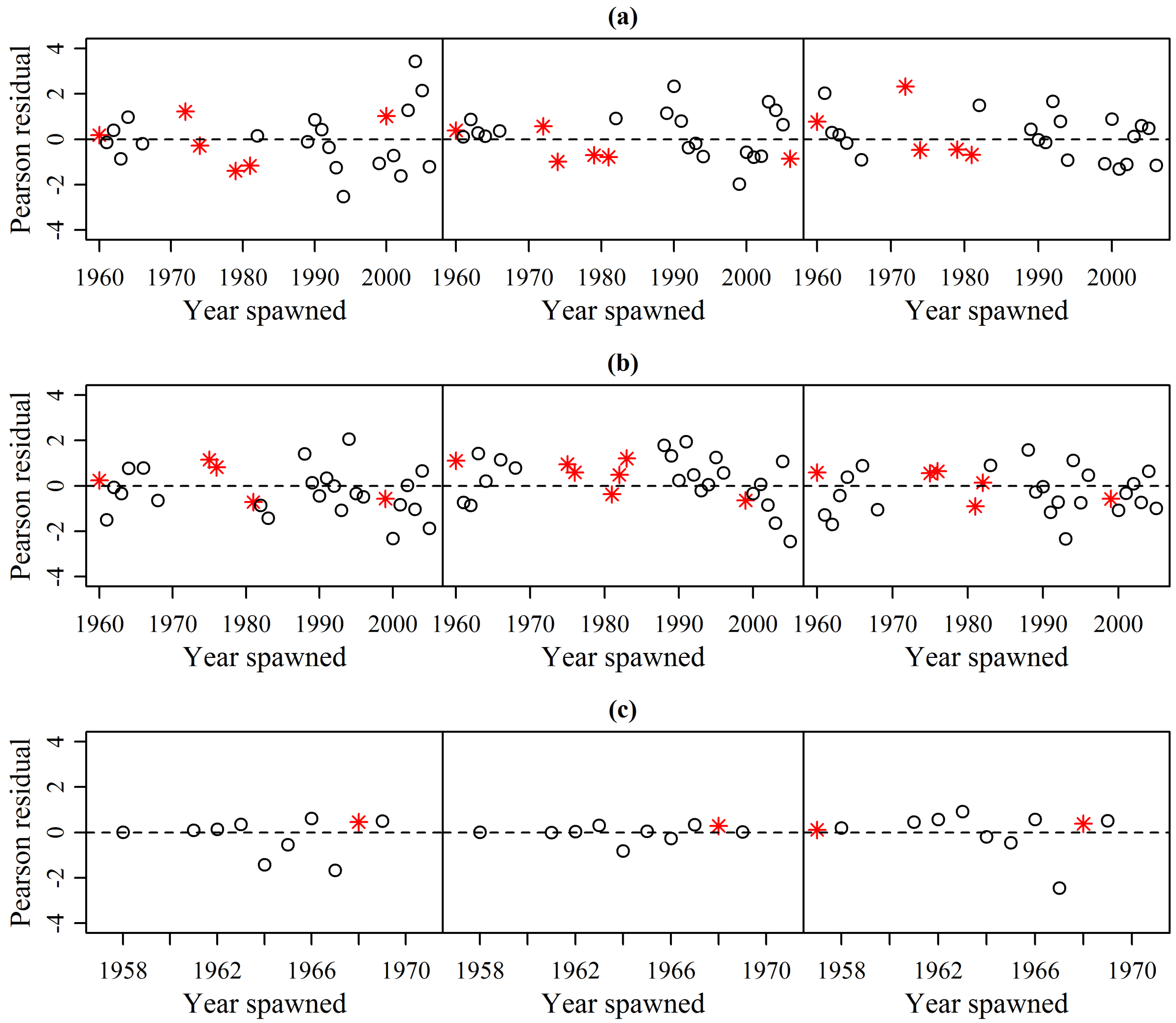

Figure S13: Pearson residuals of number of longline recoveries east of 120°E of SBT by year spawned and tagged and released in (a) WA, (b) SA and (c) EA realised assuming Submodel 2. Three sets of realised residuals are plotted from each tag release location corresponding to three random draws from the posterior. The red asterisks denote residuals associated with observations for which the conditionally expected number of longline recoveries east of 120°E is less than one.

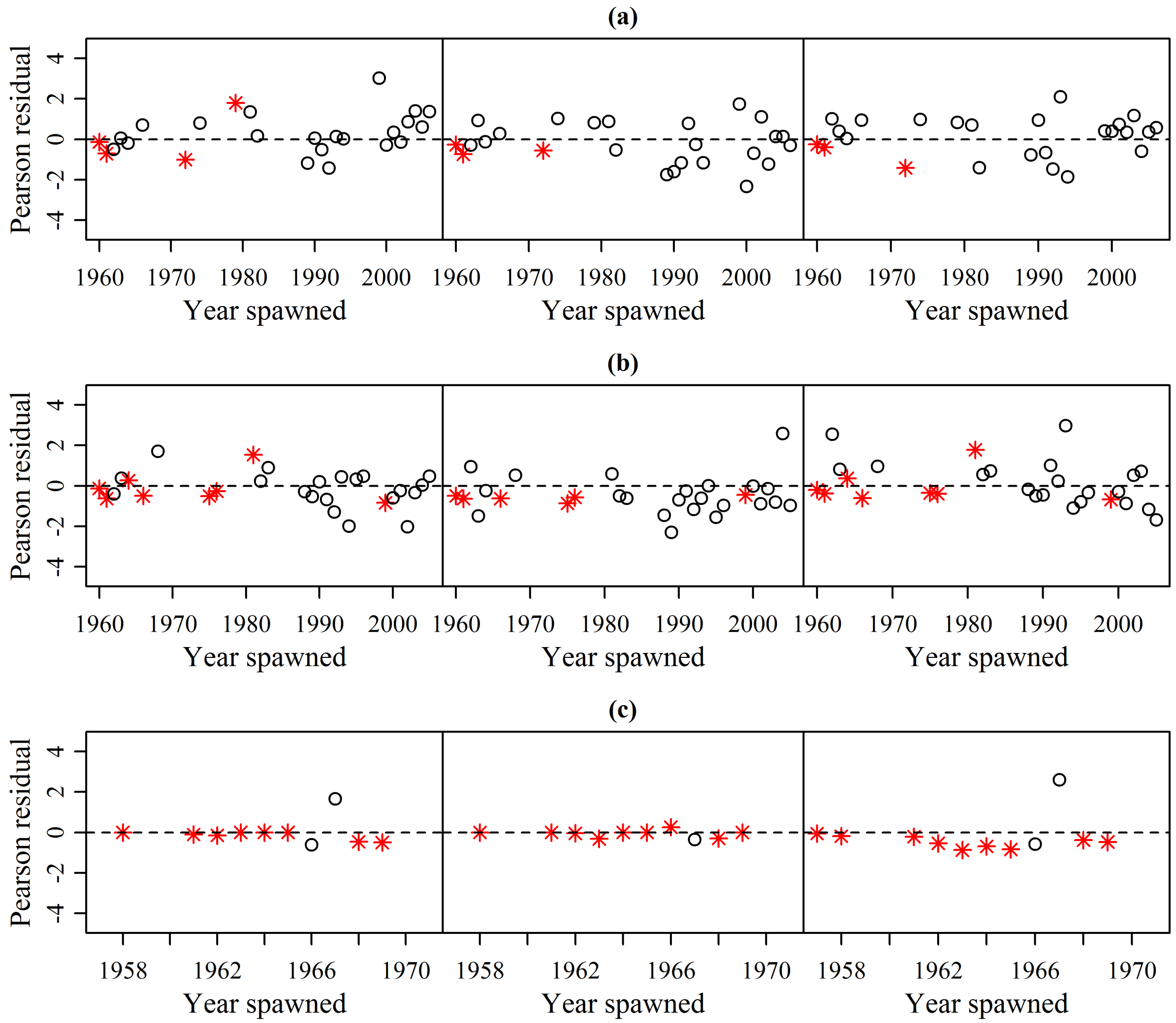

Figure S14: Pearson residuals of number of longline recoveries west of 60°E of SBT by year spawned and tagged and released in (a) WA, (b) SA and (c) EA realised assuming Sub-models 2 and 3. Three sets of realised residuals are plotted from each tag release location corresponding to three random draws from the posterior. The red asterisks denote residuals associated with observations for which the conditionally expected number of longline recoveries west of 60°E is less than one.

2.1.6 Posterior predictive checks

Analogous to Section 1.2.3 we calculate realised discrepancies and posterior predictive p-values for submodels 2 and 3 based on the Freeman-Tukey statistic. Discrepancies and p-values are calculated separately for each Submodel for each release location (Figure S15).

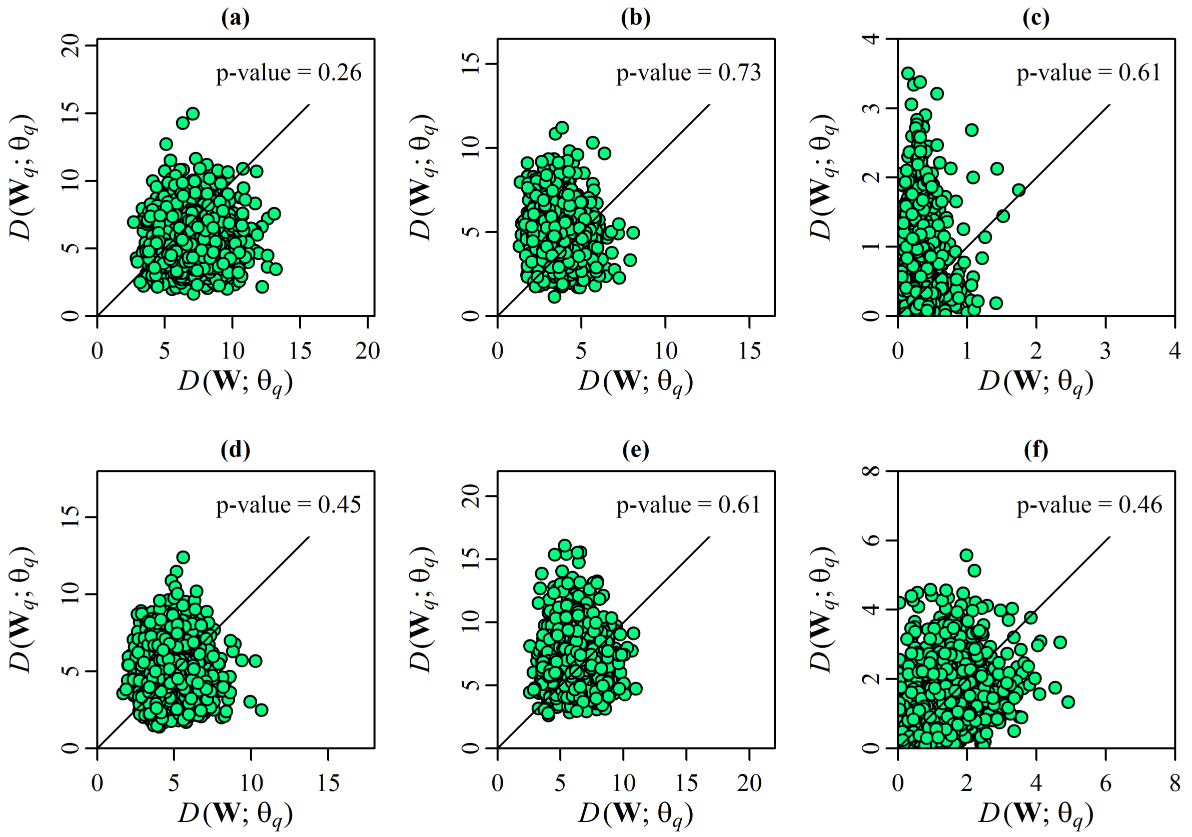

Figure S15: Freeman-Tukey discrepancy plots generated from 2000 random draws from the posterior of Submodel (2) based on releases from (a) WA, (b) SA and (c) EA and Submodel (3) given tag release from (d) WA, (e) SA and (f) EA. Posterior predictive p-values are shown in the top right corners.

The discrepancy plots and posterior predictive p-values suggest that the assumed longline model adequately describes longline recovery locations from each tag release location.

2.2 Reduced submodel for longline tag recapture location

If juvenile SBT summer in the GAB and mix throroughly, it would be expected that the recapture locations of members of a given cohort would be independent of their tagging location.

Let L_y be the total number of longline recoveries of SBT spawned in year y and tagged in WA, SA and EA and let Z_y be the number of these that were recaptured west of 120°E. We assume Z_y is a binomial random variable with probability, P_{y_a}.

$$
\begin{aligned}
&Z_y \sim \text{Binomial}(L_y, P_{y_a}), \qquad (2^*)\\
&\text{logit}(P_{y_a}) = \log\left(\frac{P_{y_a}}{1-P_{y_a}}\right) = \mu_a + \omega_{y_a},\\
&\omega_{y_a} = \kappa_a \times \omega_{(y-1)_a} + \epsilon_{y_a},\\
&\epsilon_{y_a} \sim N(0, \sigma_a^2).
\end{aligned}
$$

The priors for Model (2*) are defined as:

$$
\begin{aligned}
&\mu_a,\ \omega_{1958_a} \sim N(\text{mean} = 0, \text{std. dev.} = 10),\\
&\kappa_a \sim N(\text{mean} = 0, \text{std. dev.} = 2),\\
&\sigma_a \sim \text{half} - \text{Cauchy}(\text{scale} = 5).
\end{aligned}
$$

2.3 Reduced submodel for longline tag recapture in Western LL given west of 120° E

$$
\begin{aligned}
&W_y \sim \text{Binomial}(X_y, P_{y_b}), \qquad (3^*)\\
&\text{logit}(P_{y_b}) = \log\left(\frac{P_{y_b}}{1-P_{y_b}}\right) = \mu_b + \omega_{y_b},\\
&\omega_{y_b} = \kappa \times \omega_{(y-1)_b} + \epsilon_{y_b},\\
&\epsilon_{y_b} \sim N(0, \sigma_b^2).
\end{aligned}
$$

The priors for Model (3*) are defined as:

$$
\begin{aligned}
&\mu_b,\ \omega_{1958_b} \sim N(\text{mean} = 0, \text{std. dev.} = 10),\\
&\kappa_b \sim N(\text{mean} = 0, \text{std. dev.} = 2),\\
&\sigma_b \sim \text{half} - \text{Cauchy}(\text{scale} = 5).
\end{aligned}
$$

2.3.1 Posterior distributions of model parameters

Posterior distributions of model parameters are provided in Table S4.

Probabilities of longline recapture west of 120°E, assuming Model (2*) are shown in Figure S16.

Table S4: Posterior parameter summaries for the reduced binomial Submodels 2* and 3* for recapture ground conditional on longline fishery recovery, by year spawned, of SBT tagged and released off southern and eastern Australia. Parameters with a subscripts pertain to submodel 2* and parameters with b subscripts pertain to submodel 3*.

Parameter	Mean	Std. Dev.	95% Cred. Int.	N_{eff}	$\hat{R}$
μ_a	-0.32	0.17	$(-0.67,\ 0.01)$	6.5×10^4	1
κ_a	0.87	0.11	$(0.63,\ 1.02)$	9.9×10^3	1
σ_a	0.74	0.16	$(0.49,\ 1.11)$	3.2×10^4	1
μ_b	-0.22	0.26	$(-0.79,\ 0.26)$	2.1×10^4	1
κ_b	0.79	0.15	$(0.46,\ 1.01)$	1.1×10^4	1
σ_b	0.59	0.15	$(0.49,\ 0.94)$	2.1×10^4	1

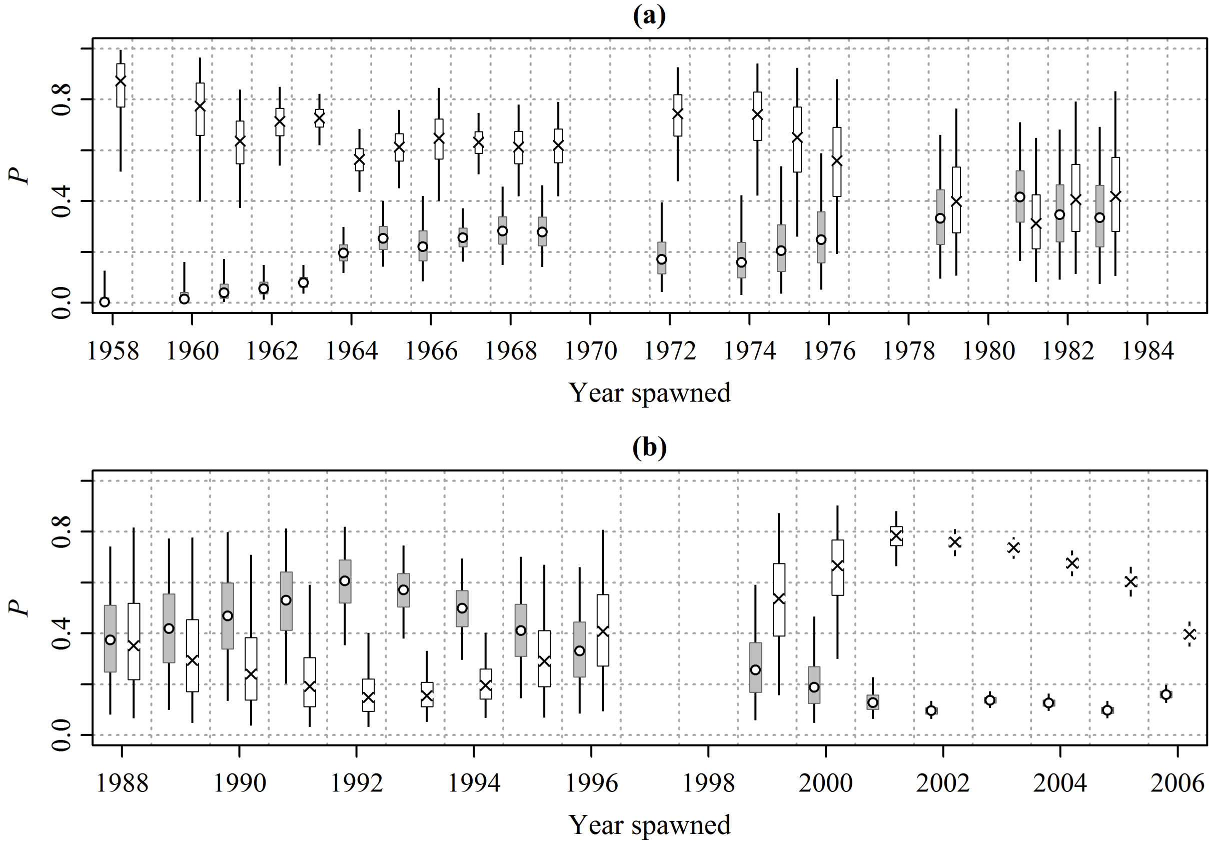

Figure S16: Posterior distributions of probability of recapture east of 120°E (crosses and white boxes) and west of 60°E (open circles and grey boxes) conditional on longline recovery of cohorts (a) spawned before 1987 and (b) spawned since 1987 that werre tagged and released from any of WA, SA or EA described by Submodels 2* and 3*. The boxes indicate posterior 50% credible intervals and the thin vertical lines are 95% credible intervals. Crosses and open circles are posterior medians.

2.3.2 Realised Pearson residuals for longline recovery locations

Pearson residuals realised from three random draws from the posterior distribution are shown in Figure S18. It can be seen that residuals associated with releases from WA tend to be positive and residuals associated with releases from EA tend to be negative. These anomalies are consistent across the three replicates of each set of residuals. Based on this observation alone we would conclude that Model (2*) does not adequately describe longline recaptures west of 120°E.

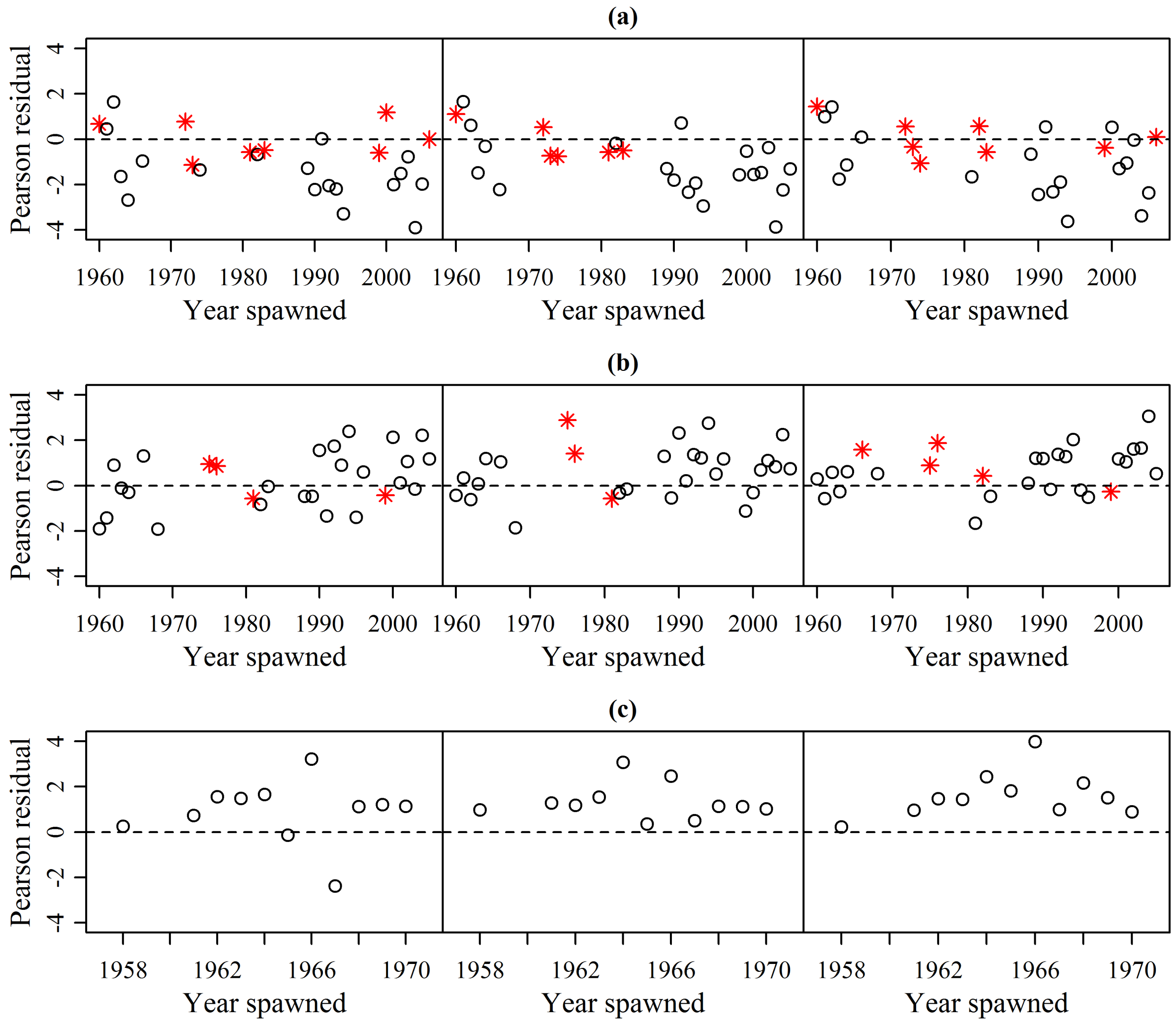

Figure S17: Pearson residuals of number of longline recoveries west of 120°E of SBT by year spawned and tagged and released in (a) WA, (b) SA and (c) EA realised assuming Submodel (2*). Three sets of realised residuals are plotted from each tag release location corresponding to three random draws from the posterior. The red asterisks denote residuals associated with observations for which the conditionally expected number of longline recoveries east of 120°E is less than one.

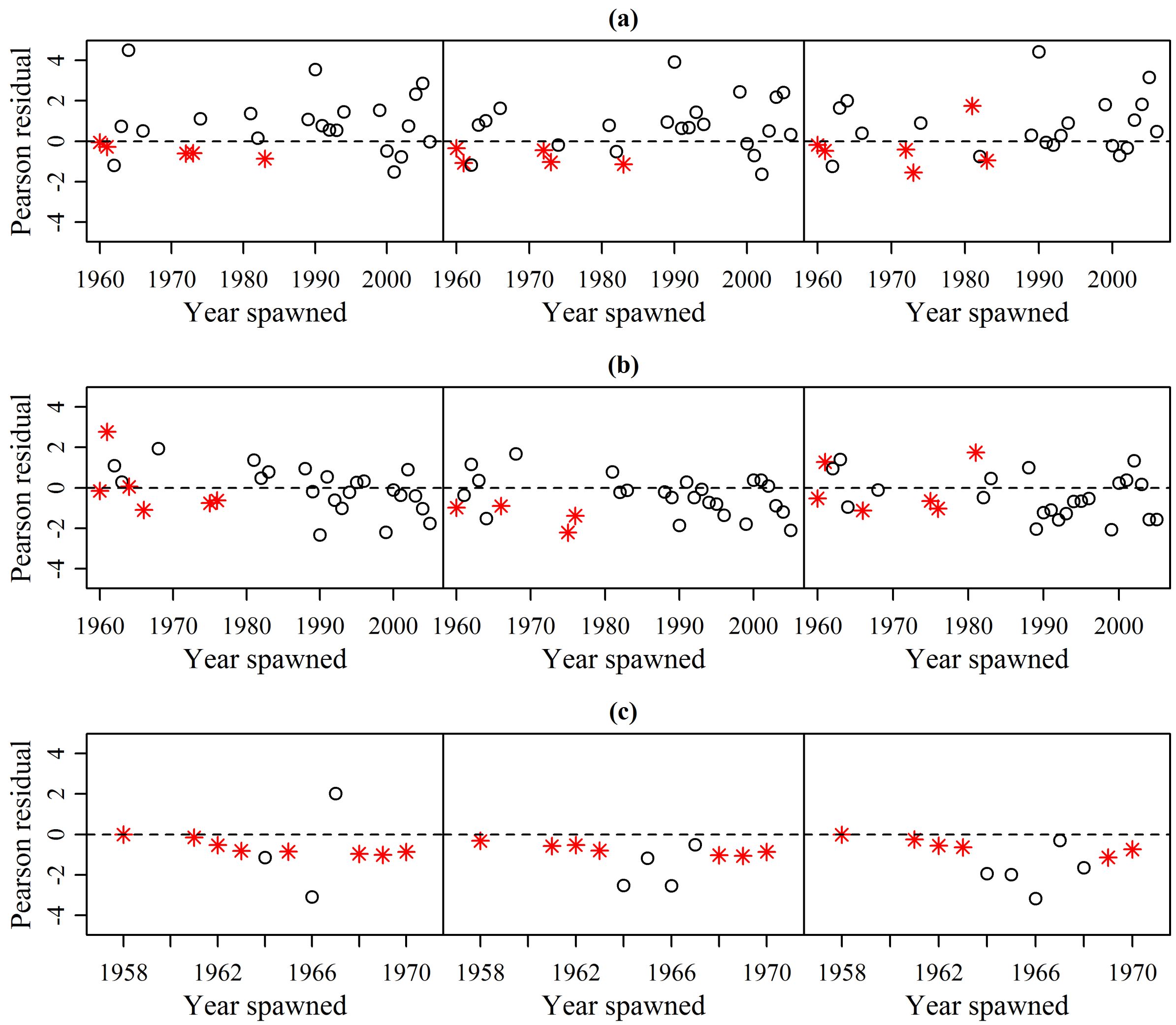

Figure S18: Pearson residuals of number of longline recoveries west of 120°E of SBT by year spawned and tagged and released in (a) WA, (b) SA and (c) EA realised assuming Submodels 2* and 3*. Three sets of realised residuals are plotted from each tag release location corresponding to three random draws from the posterior. The red asterisks denote residuals associated with observations for which the conditionally expected number of longline recoveries west of 60°E is less than one.

2.3.3 Posterior predictive checks

The discrepancy plots (Figure S19) indicate that the reduced Model (2*) does not fit the observed longline recoveries nearly as well as the assumed Model (2).

We note that the Freeman-Tukey goodness of fit statistic does not distinguish between positive and negative residuals, only their magnitude. Therefore, the imbalance of positive and negative residuals evident in Figure S18a and Figure S18c respectively is not captured by the posterior predictive checks presented in this section.

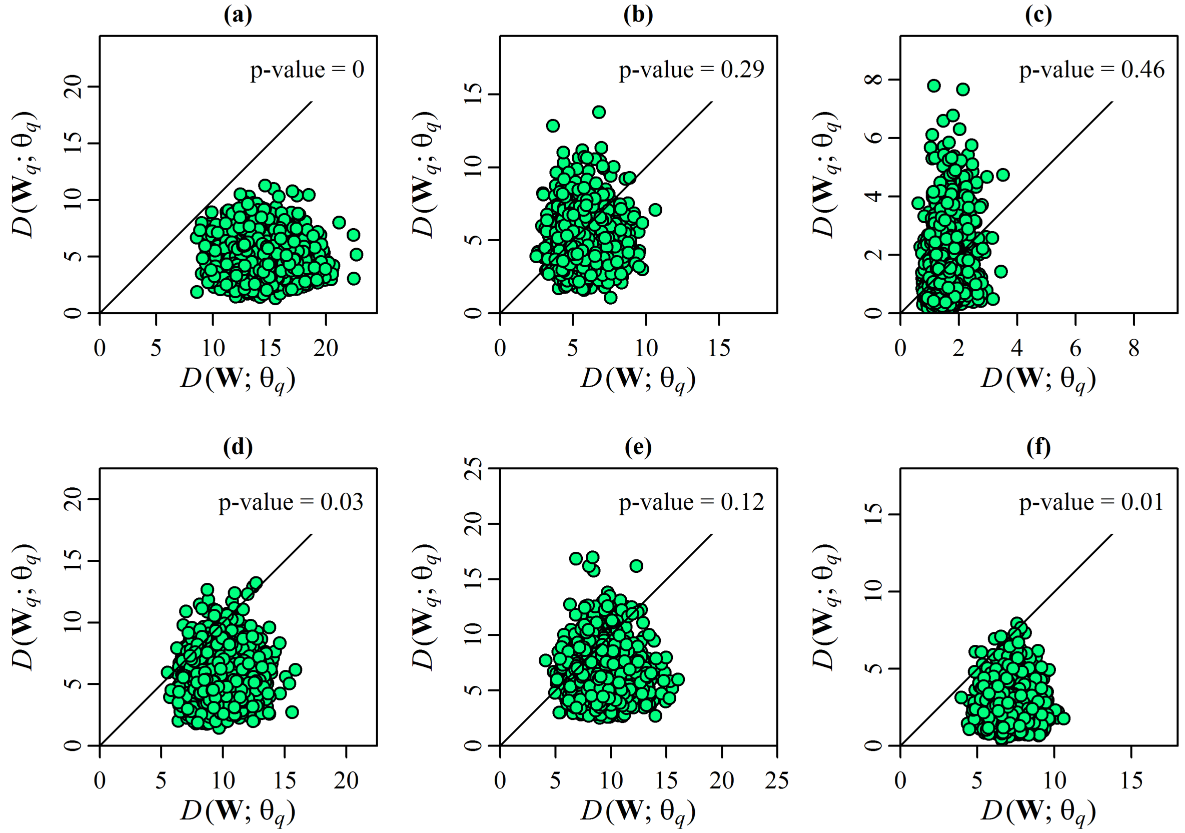

Figure S19: Freeman-Tukey discrepancy plots generated from 2000 random draws from the posterior of Submodel 2* based on releases from (a) WA, (b) SA and (c) EA and Submodel 3* given tag release from (d) WA, (e) SA and (f) EA. Posterior predictive p-values are shown in the top right corners.

References

Brooks, S. P., Catchpole, E. A. & Morgan, B. J. (2000), 'Bayesian animal survival estimation', *Statistical Science* pp. 357–376.

Collett, D. (2002), *Modelling Binary Data*, CRC.

Freeman, M. F. & Tukey, J. W. (1950), 'Transformations related to the angular and the square root', *The Annals of Mathematical Statistics* pp. 607–611.

Gelman, A., Carlin, J. B., Stern, H. & Rubin, D. B. (2004), *Bayesian Data Analysis*, 2nd ed. edn, Chapman & Hall/CRC, Boca Raton, FL.

Gelman, A. & Rubin, D. B. (1992), 'Inference from iterative simulation using multiple sequences', *Statistical Science* **7**(4), 457–472.

Meng, X.-L. (1994), 'Posterior predictive p-values', *The Annals of Statistics* **22**(3), 1142–1160.

Stan Development Team (2014), 'Rstan: the R interface to Stan, version 2.5.0'.
URL: *http://mc-stan.org/rstan.html*
