# Supplementary material for: Evidence of separate subgroups of juvenile southern bluefin tuna
Source: Ecol Evol. 2017 Nov 2;7(22):9818–44. doi: 10.1002/ece3.3500 (PMC5696402; doi:10.1002/ece3.3500)
Supplement: Supplementary file 3 [file ECE3-7-9818-s003.pdf]

# Evidence of separate subgroups of juvenile southern bluefin tuna - supplementary material 3

Mark S. Chambers[1], Leesa A. Sidhu[1], Ben O'Neill[1], and Nokuthaba Sibanda[2]

[1]School of Physical, Environmental and Mathematical Sciences, University of New South Wales at the Australian Defence Force Academy, Canberra.
[2]School of Mathematics and Statistics, Victoria University of Wellington.

## 1 Test of complete mixing from longline recoveries

Latour et al. (2001) describe a simple $\chi^2$ test of recapture locations of tags recovered from subgroups of a cohort to examine evidence of incomplete mixing. We compare longline recovery locations of twelve cohorts of SBT tagged and released from Western Australia and South Australia during tagging studies run during the 1990s and 2000s. The test is applied to individuals tagged at three years of age or less and recaptured between the ages of three and six. The results are shown in Table S5.

## References

Latour, R. J., Hoenig, J. M., Olney, J. E. & Pollock, K. H. (2001), 'A simple test for nonmixing in multiyear tagging studies: Application to striped bass tagged in the Rappahannock River, Virginia', *Transactions of the American Fisheries Society* **130**(5), 848–856.
**URL:** *http://dx.doi.org/10.1577/1548-8659(2001)130<0848:ASTFNI>2.0.CO;2*

Table S5: Observed and expected recapture locations of longline recoveries from twelve cohorts tagged at three years of age and below during the 1990s and 2000s tagging studies. Recaptures at ages between three and six years inclusive. Expected recoveries are recoveries expected in the case of complete mixing of cohorts. $\chi^2$ statistics and p-values of the null hypothesis of complete mixing of cohorts. Cohorts with significant evidence of incomplete mixing are bolded.

| | | Western LL | | Central LL | | Eastern LL | | | | |
|---|---|---|---|---|---|---|---|---|---|---|
| Cohort | Tag state | Obs. | Exp. | Obs. | Exp. | Obs. | Exp. | $\chi^2$ | df | $p$-value |
| 1989 | WA | 3 | 1.7 | 4 | 2.8 | 13 | 15.5 | | | |
| | SA | 14 | 15.3 | 23 | 24.2 | 139 | 136.5 | 2.1 | 2 | 0.35$^\dagger$ |
| **1990** | **WA** | **22** | **11.1** | **3** | **7.0** | **39** | **45.9** | | | |
| | **SA** | **33** | **43.9** | **32** | **28.0** | **189** | **182.1** | **17.7** | **2** | **<0.001** |
| 1991 | WA | 7 | 7.0 | 7 | 10.4 | 33 | 29.6 | | | |
| | SA | 31 | 31.0 | 49 | 45.6 | 127 | 130.4 | 1.81 | 2 | 0.40 |
| 1992 | WA | 14 | 9.8 | 26 | 24.1 | 48 | 54.1 | | | |
| | SA | 14 | 18.2 | 43 | 44.9 | 107 | 100.9 | 4.1 | 2 | 0.13 |
| 1993 | WA | 30 | 24.3 | 49 | 49.4 | 52 | 57.3 | | | |
| | SA | 29 | 34.7 | 71 | 70.6 | 87 | 81.7 | 3.1 | 2 | 0.21 |
| **1994** | **WA** | **32** | **27.6** | **62** | **52.6** | **42** | **55.8** | | | |
| | **SA** | **21** | **25.4** | **39** | **48.4** | **65** | **51.2** | **12.0** | **2** | **0.002** |
| 2000 | WA | 8 | 7.6 | 19 | 18.9 | 2 | 2.5 | | | |
| | SA | 10 | 10.4 | 26 | 26.1 | 4 | 3.5 | 0.23 | 2 | 0.89$^\dagger$ |
| 2001 | WA | 23 | 24.9 | 82 | 77.7 | 4 | 6.4 | | | |
| | SA | 20 | 18.1 | 52 | 56.3 | 7 | 4.6 | 3.0 | 2 | 0.22$^\dagger$ |
| 2002 | WA | 16 | 18.3 | 75 | 70.2 | 6 | 8.6 | | | |
| | SA | 16 | 13.7 | 48 | 52.8 | 9 | 6.4 | 3.2 | 2 | 0.20 |
| 2003 | WA | 8 | 5.6 | 19 | 197.7 | 7 | 8.7 | | | |
| | SA | 29 | 31.4 | 111 | 110.3 | 50 | 48.3 | 1.6 | 2 | 0.45 |
| **2004** | **WA** | **20** | **13.5** | **23** | **19.1** | **9** | **19.4** | | | |
| | **SA** | **19** | **25.5** | **32** | **35.9** | **47** | **36.6** | **14.5** | **2** | **<0.001** |
| **2005** | **WA** | **12** | **6.3** | **18** | **19.4** | **5** | **9.3** | | | |
| | **SA** | **5** | **10.7** | **34** | **32.6** | **20** | **15.7** | **11.4** | **2** | **0.003** |
